# Differential RNA-seq, Multi-Network Analysis and Metabolic Regulation Analysis of *Kluyveromyces marxianus* Reveals a Compartmentalised Response to Xylose

**DOI:** 10.1371/journal.pone.0156242

**Published:** 2016-06-17

**Authors:** Du Toit W. P. Schabort, Precious K. Letebele, Laurinda Steyn, Stephanus G. Kilian, James C. du Preez

**Affiliations:** Department of Microbial, Biochemical and Food Biotechnology, University of the Free State, Bloemfontein, South Africa; University Paris South, FRANCE

## Abstract

We investigated the transcriptomic response of a new strain of the yeast *Kluyveromyces marxianus*, in glucose and xylose media using RNA-seq. The data were explored in a number of innovative ways using a variety of networks types, pathway maps, enrichment statistics, reporter metabolites and a flux simulation model, revealing different aspects of the genome-scale response in an integrative systems biology manner. The importance of the subcellular localisation in the transcriptomic response is emphasised here, revealing new insights. As was previously reported by others using a rich medium, we show that peroxisomal fatty acid catabolism was dramatically up-regulated in a defined xylose mineral medium without fatty acids, along with mechanisms to activate fatty acids and transfer products of β-oxidation to the mitochondria. Notably, we observed a strong up-regulation of the 2-methylcitrate pathway, supporting capacity for odd-chain fatty acid catabolism. Next we asked which pathways would respond to the additional requirement for NADPH for xylose utilisation, and rationalised the unexpected results using simulations with Flux Balance Analysis. On a fundamental level, we investigated the contribution of the hierarchical and metabolic regulation levels to the regulation of metabolic fluxes. Metabolic regulation analysis suggested that genetic level regulation plays a major role in regulating metabolic fluxes in adaptation to xylose, even for the high capacity reactions, which is unexpected. In addition, isozyme switching may play an important role in re-routing of metabolic fluxes in subcellular compartments in *K*. *marxianus*.

## Introduction

The yeast *Kluyveromyces marxianus* is emerging as a host for metabolic engineering and recombinant protein production, having a number of advantages over *Saccharomyces cerevisiae*. These characteristics include thermotolerance and the ability to utilise a wide variety of sugars, including the pentose xylose, that are abundant in lignocellulosic biomass. Moreover, it is probably the fastest growing eukaryote on Earth [1]. Previous studies of this yeast highlighted the large physiological variation among *K*. *marxianus* strains, in terms of their proneness to fermentation and ability to utilize various substrates, suggesting genetic diversity within the species [2]. Our strain UFS-Y2791 produces a significant amount of ethanol even under aerobic conditions, suggesting that this strain is not typically Crabtree negative. We recently assembled a draft genome of strain UFS-Y2791 from our University of the Free State MIRCEN yeast culture collection. The strain was originally isolated from juice prepared from the arid region succulent *Agave americana*. To enable metabolic engineering of *K*. *marxianus* and other yeasts, it is important to understand genetic responses to environmental factors and different substrates. Such an understanding requires both advanced high-throughput omics methods as well as various integrative computational methods. Thus far, only two studies of genome-scale transcript levels in *K*. *marxianus* have been published. Work on strain DMKU 3–1042 suggested that the yeast up-regulated β-oxidation in the absence of glucose repression in a complex xylose medium due to the use of lipids as additional carbon source in the absence of glucose, implying possible utilization of the small amounts of lipids present in the rich medium [3]. A question that may also arise here is that in case lipids were indeed present in the rich medium, whether the response in the β-oxidation was due to glucose de-repression or due to stimulation by lipids. Another study focussed on the response of strain Y179 to anaerobic versus micro-aerobic conditions, as well as to the concentration of inulin in the medium. This study focussed on highlighting differential expression in various stress-response genes, those involved in autophagy, and a number of individual key enzymes. Both of these recent studies used a complex medium containing peptone and yeast extract [4].

Next-generation sequencing (NGS) has become a popular method not only for sequencing genomes but also for various other experiments [5,6]. RNA-seq is a method in which RNA transcripts are reverse-transcribed and quantitatively sequenced [7]. This method can accurately quantify differential expression and the improved sensitivity and dynamic range, as well as the ability to elucidate splice variants, makes it superior to microarray technology. The combination of genome sequencing and RNA-seq of a new species or strain is a powerful approach to rapidly gain both a blueprint (genome) and a response (transcriptome) to some perturbations [3] or when comparing different species [8]. Innovative methods are now needed to effectively use both the blueprint and the response in a concise manner that is attractive to scientists, since a massive amount of data is generated in a single experiment. It is a major challenge to investigate and represent omics results at the genome scale. Gene set enrichment using Gene Ontology (GO) is an established method for microarrays and is now becoming established for RNA-seq (reviewed in [9]).

Analysis of metabolic pathways could be a sensible additional approach as it gives a sense of directionality to the response, and the scientist often associates a certain endpoint metabolite with a pathway, providing a means to simplify the understanding of the dataset. Painted renderings could be made of individual pathways, but often the number of pathways excludes a concise representation, and the integrated nature of metabolism is lost. Further, in metabolic pathways the interactions (reactions) consist of hypergraphs containing more than one substrate or product, complicating the rendering. Cellular overviews, as is available with software such as Pathway Tools, summarises metabolism into a single image [10]. However, the cellular overview method usually assumes a single master framework and inevitably ignores selected highly connected nodes. Cellular compartmentalisation is also often neglected in omics analysis due to the added complexity.

An approach that is complementary to the pathway-based understanding of metabolism is that of the study of metabolite levels. Metabolomics, which entails the characterisation and quantification of small compounds in the cell, is still technologically demanding and labour intensive, however (reviewed in [11,12]). A new approach in systems biology is to derive compounds that are likely differentially expressed or extensively homeostatically regulated, from a differential transcriptomics dataset. These are the reporter metabolites [13] and the method could be generalised and applied in many scenarios such as described elsewhere [14].

Models of biochemical pathways have much potential to reveal new insights that are not obvious from the exploration of datasets. The understanding of complex biochemical datasets using computational models is called systems biology. Some flux modelling approaches such as Flux Balance Analysis (FBA) simulations could be done even at the genome scale [15,16], as was recently reported for *K*. *lactis* [17]. A long-standing fundamental question is how the flux through a metabolic pathway is regulated. Is the change in a given flux achieved by changes in the concentrations of metabolites that affect that enzyme (metabolic level), or through changes in gene expression or post-translational modifications (hierarchical level)? By combining flux models with measured metabolite exchange fluxes, or through more complex ^13^C-Metabolic Flux Analysis, estimation of fluxes at different physiological states can be very informative. Metabolic Regulation Analysis (MRA) combines differential expression levels with differential flux estimates to reveal for each flux the contribution of the metabolic and hierarchical levels of regulation [18].

Here we report on a detailed RNA-seq transcriptomics study to explore the response of *K*. *marxianus* UFS-2791 to glucose and xylose in a chemically defined medium under aerobic conditions, including a number of different systemic analyses. Although a major potential future application of *K*. *marxianus* may be ethanol production from lignocellulosic biomass, which is an anaerobic or oxygen limited process where both glucose and xylose may be present, we chose to perform aerobic cultivations with glucose or xylose separately to remove any confounding effects. The strain also utilizes these sugars sequentially. The high cultivation temperature of 35°C was also chosen as this strain was determined to have a growth optimum close to 35°C. Our samples were also taken during mid-exponential phase to eliminate the effect of any possible ethanol stress that may occur later during the fermentation. To address the need for effective integrative exploration of omics data, including RNA-seq, and to combine these data with modelling and simulation, we developed the Reactomica software. We combined gene set enrichment of Gene Ontology (GO), reporter metabolites, metabolic pathway maps with a strong focus on subcellular compartmentalisation, and two new approaches of representation, namely pathway-to-pathway networks and reporter metabolite-enzyme networks.

The aims were to first effectively explore the key features of the differential response to xylose in a defined medium under aerobic conditions and determine whether the peroxisomal lipid catabolic response previously observed [3] was limited to a rich medium. Subsequently, the central carbon metabolism was investigated in detail, separating the response into subcellular compartments. It is known that in most yeasts that are able to utilize xylose, the two-step conversion via NADPH dependent xylose reductase and the NAD^+^ dependent xylitol dehydrogenase is present and that the co-factor independent isomerase reaction is absent, as in *K*. *marxianus* [3]. Under aerobic conditions, the additional NADH produced by xylitol dehydrogenase is easily oxidised by the electron transport chain. However, the yeast would require additional NADPH for xylose reductase. We investigated which of the key NADPH producing reactions would be up-regulated to support this proposed additional requirement for NADPH during xylose utilization. The somewhat unexpected results were rationalised by estimating fluxes in central metabolism for both conditions. Finally, MRA was used to answer the fundamental question of how changes in gene expression and in metabolite concentrations, respectively, contributed to the regulation of fluxes.

## Materials and Methods

### Genome sequencing and annotation

The genome of *K*. *marxianus* UFS-Y-2791 was sequenced on the Illumina HiSeq platform to 100-fold coverage at the Onderstepoort Biotechnology Platform, Pretoria, South Africa. Assembly was performed *de novo* using Abyss. Open reading frames were found by Augustus. Putative protein sequences were annotated using one of two methods. For annotation of enzymes (778 genes), creation of a pathway genome database (PGDB) and all subsequent analysis involving metabolic pathways, protein sequences were annotated against the Kegg-Kaas server with default settings on the server, resulting in a list of EC number annotations. Output was subsequently parsed and converted to input for the PathoLogic algorithm of Pathway Tools. For gene set enrichment using Gene Ontology (GO), sequences were additionally annotated against the UniProtKB database on a high-performance computing cluster, using BLASTP. An E-value cut-off of 1E-10 was used for gene set enrichment as was done by others [3]. An additional 73 genes with E-values between 1E-5 and 1E-10 were included in the list of annotated genes and flagged for further annotation. We preferred the rich manually curated SwissProt annotations over automated Trembl annotations, resulting in 68.3% of annotations from *S*. *cerevisiae*, 17.4% from *K*. *lactis*, 3.2% and 2.8% from two strains of *K*. *marxianus*, and the rest from other species. The draft genome, predicted open reading frames, predicted protein sequences and functional annotations are made available in the Supplementary materials (S1 Draft Genome, S1 ORF, S1 Proteins, S1 Table).

### Strains and cultivation

All chemicals and fermentation media used in cultivations were obtained from Sigma Aldrich, Seelze, Germany. *K*. *marxianus* UFS-Y2791 from our University of the Free State MIRCEN yeast culture collection was maintained on YPD agar slants at 4°C. Cultivation was carried out under aerobic conditions in 500 ml shake flasks with 30 ml working volume at 180 rpm. We chose the shake flask format to allow expensive ^13^C-isotopic tracer studies which would be cost prohibitive in bioreactors. All cultivations were carried out at 35°C. The pre-inoculum was incubated in YPD medium for 8 h. The inoculum was prepared by dilution of the pre-inoculum to an OD_690_ of 0.05 in a chemically defined medium and grown at 35°C for 16 h. The defined medium contained (g l^-1^): glucose or xylose, 5; (NH_4_)_2_SO_4_, 2.5; MgSO_4_·7H_2_O, 0.5; CaCl_2_·H_2_O, 0.03; NaCl, 0.1; citric acid, 0.25 and KH_2_PO_4_, 10. Filter-sterilised vitamins were added to the autoclaved medium at the following concentrations (mg l^-1^): biotin, 0.025; calcium pantothenate, 0.5; nicotinic acid, 0.5; *p*-aminobenzoic acid, 0.1; pyridoxine HCl, 0.5; thiamine HCl, 0.5 and *myo*-inositol, 12.5. Trace elements were added according to du Preez and van der Walt [19]. The pH of the medium was adjusted to 5.5. Glucose and xylose were quantified using HPLC. Acetate, ethanol and glycerol were the only fermentation metabolites secreted in significant amounts and were quantified using HPLC. All samples were taken before an OD_600_ of 0.8 was reached.

### RNA-seq data generation

Cells from duplicate cultivations were harvested in mid-exponential phase at an OD_690_ of 0.8 by centrifugation at 1 000 × g at 4°C for 5 min. RNA extraction was carried out according to the RNeasy yeast RNA kit (Qiagen) protocol, including a DNAase step. Ribosomal RNA was removed by the Ribo-Zero rRNA Removal Kit (Illumina) and remaining RNA was sequenced at the Onderstepoort Biotechnology platform in Pretoria, South Africa. Paired-end reads were generated using Illumina HiSeq Next Generation Sequencing. For each of duplicate samples, 3.75 million trimmed paired-end reads were mapped to the UFS-Y2791 genome. Processing of sequencing data was carried out in Galaxy. Reads were trimmed using Trimmomatic [20] and mapped to the genome using TopHat, while CuffDiff [20] was used to test for differential expression. CuffDiff reports p-values as the statistical significance and well as the q-value, which is the p-value after accounting for multiple comparisons [21]. Genes were only considered to be significantly differentially expressed when q-values were below 0.05.

### Gene set enrichment and reporter metabolites

For gene set enrichment and all other network constructions and renderings, programs were developed as part of a new software suite for integrative systems biology that we call Reactomica, implemented in the Wolfram Mathematica language. Gene set enrichment scores for GO terms and pathways were calculated similar to that described by Ideker [22] as follows: GO ontologies goslim_yeast.obo and go-basic.obo.txt.m were obtained from the Gene Ontology database [23]. The ontologies were converted to graphs using the ‘is_a’ child-parent mappings. For obtaining the gene set, a depth-first scan was performed from each GO term and all additional GO terms found from the node of interest were collected to ensure that highly specialised terms would find utility in GO_slim. Mappings from the annotated genes to the GO-terms in the GO ‘biological_process’, ‘molecular_function’ and ‘cellular_component’ attributes of a UniProt entry were used to map from GO terms to genes. Significance q-values could be converted to Z-scores as the negative of the inverse cumulative distribution function and then summed over all genes in the gene set to give the representative statistic for a group of genes as the total Z-score [22]. Random gene sets were generated with bootstrapping (1000 iterations) and total Z-scores calculated. The mean and standard deviation at a variety of gene set sizes were calculated and used to calculate the enrichment score S:

$$\text{S}=\frac{\text{Z}\left(\text{total},\text{Test}\right)-\text{Mean}\left(\text{Z},\text{Background}\right)}{\text{Standard deviation}\left(\text{Z},\text{Background}\right)}$$ (1)

For pathway gene set analysis, MetaCyc pathways were used from the BioCyc pathway genome database constructed for this strain using Pathway Tools, which is based on the MetaCyc database. Reporter metabolite enrichment scores were calculated in the same manner as described above and according to Patil and Nielsen [13]. For reporter metabolites, the background mean and standard deviation of random genes sets were rather generated by sampling enzyme-encoding genes only, as opposed to the complete gene set, since a large fraction of the differentially expressed genes were metabolic, generating a higher random background enrichment.

### Pathway maps

To explore the metabolic response, metabolic pathway maps were created from MetaCyc pathways and RNA-seq data were mapped using various colouring schemes with Reactomica, harnessing automated hypergraph map layout and manual override. The initial linkage between genes and reactions were made by the Pathway Tools algorithm PathoLogic, with genomic annotations from the Kegg-Kaas annotation server. For the Log_2_(fold change) colouring scheme, in the case of more than one enzyme that could perform the same function, the largest fold change in expression was used for the colour rendering. Additional gene-reaction linkages were made from a UniProt BLASTP annotation. Subsequent compartmentalisation made use of the GO ‘cellular_component’ ontology terms. For purpose, a mapping was built into Reactomica that maps GO ‘cellular_component’ ontology terms to subscellular compartments.

### Pathway-to-pathway networks

Pathways were clustered by the number of metabolites in common to generate a scoring matrix. The number of metabolites in common was normalised by the smaller of the two metabolite sets of a pathway and a threshold was selected for including a mapping that resulted in optimal rendering. Orphaned pathways were clustered together.

### Molecular networks

Molecules were clustered by the simple similarity criterion of string matching of SMILES strings of each compound in the PGDB using the edit distance. Only the closest match was included as a mapping. The method is similar to that done by Barupal et al. [24].

### Reporter metabolite-enzyme networks

The metabolic network was converted from a hypergraph into a graph in which only the interactions between differentially expressed enzyme genes and enriched reported metabolites were retained.

### Differential Flux Analysis

The aim was to approximate the fluxes on glucose and xylose, sufficient for approximation of MRA values. FBA was used which included optimisation of biomass formation, and measured specific sugar consumption rate and specific ethanol, acetate and were included to constrain the flux solution. The model was constructed in Reactomica using reactions from MetaCyc for which representative genes were found in the genome annotation of *K*. *marxianus* UFS-Y2791. Some additional reactions were defined such as the combined electron transport and oxidative phosphorylation reaction. The biomass reaction was adapted from Fischer et al. [25]. The flux model and parameters is provided in S5 Table. FBA was implemented in Reactomica and the method of FBA was reviewed elsewhere [15]. FBA uses linear optimisation, maximising the biomass formation reaction, constrained by the reaction stoichiometry, reversibility constraints, uptake rate of nutrients (glucose or xylose) and production rates (ethanol and acetate).

### Metabolic Regulation Analysis

The metabolic regulation of a flux can be separated into a metabolic component ρm and a hierarchical component ρh. The two levels of regulation are combined in the relation below [18].

1=ρh+ρm (2)

The metabolic component ρm models the contribution to regulation that changes in metabolite concentrations make and is described by

$$\mathrm{\rho m}=\sum_{X}\frac{d\;ln\left(v\right)}{d\;ln\left(X\right)}.\frac{d\;ln\left(X\right)}{d\;ln\left(J\right)}$$ (3)

where *J* is the flux though that enzyme, modelled by the rate equation *v*, and affected by changes in the concentration of metabolite *X*. The hierarchical component ρh models the effect of changes in maximal activity of the enzyme, which is usually linearly dependent on the enzyme concentration *e* and described by:

$$\mathrm{\rho h}=\frac{d\;ln\left(e\right)}{d\;ln\left(J\right)}$$ (4)

The hierarchical component could thus be experimentally determined by measuring a difference in maximal enzyme activity. By using Eq 2 and substituting with the experimentally determined *ρh*, *ρm* could also be calculated. Though maximal activity is affected not only by the protein concentration and post-translational modifications, it is reasonable and practical to use changes in transcript levels obtained from RNA-seq instead of maximal activities or protein levels for an estimated MRA. For MRA, gene expression changes were considered significant if at least one gene (among paralogs) was considered significant from q-values. Transcript fold changes were calculated from total transcript abundances for all genes mapping to a reaction, in case of isozymes resulting from paralogs or multi-functional proteins. In this manner, the method is robust to potential errors in annotation of paralogs since central metabolic genes are known to have on average higher transcript abundance in comparison with the vast majority of other genes. The fold changes of individual minor isozymes with very low expression levels also cannot dominate the analysis.

## Results

**Fig 1:**
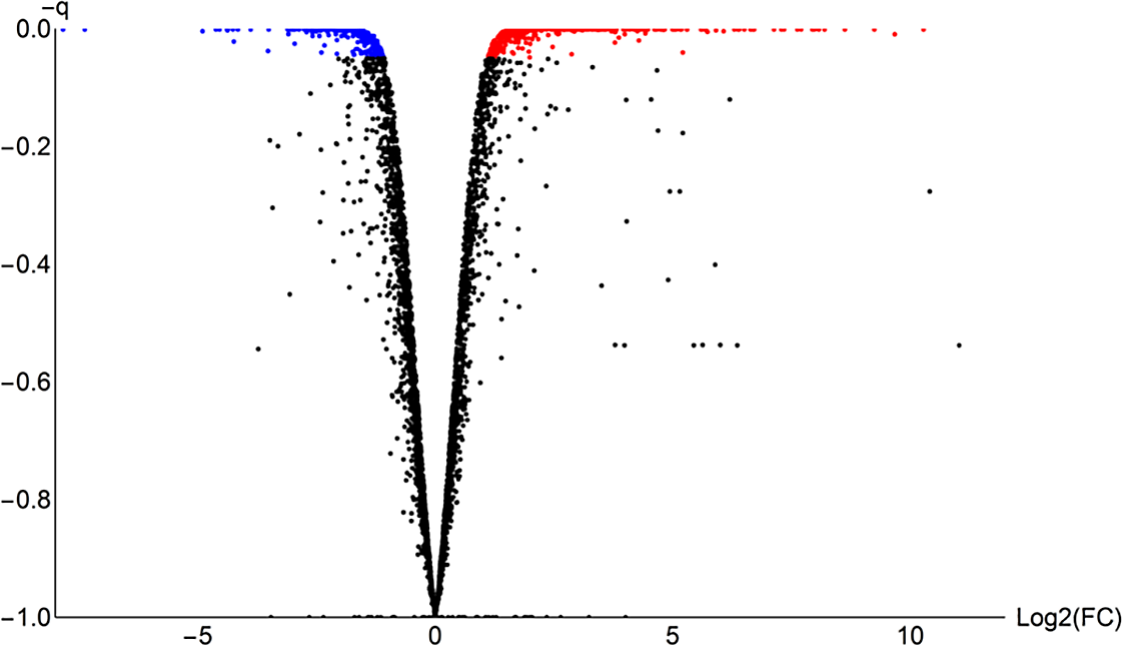
Volcano plot of RNA-seq data. FC: fold change.–q: the corrected p- values after taking multiple comparisons into account, as performed by CuffDiff. Red: up-regulated on xylose. Blue: down-regulated on xylose. Black: constitutively expressed. Statistical procedures were performed in CuffDiff.

**Fig 2:**
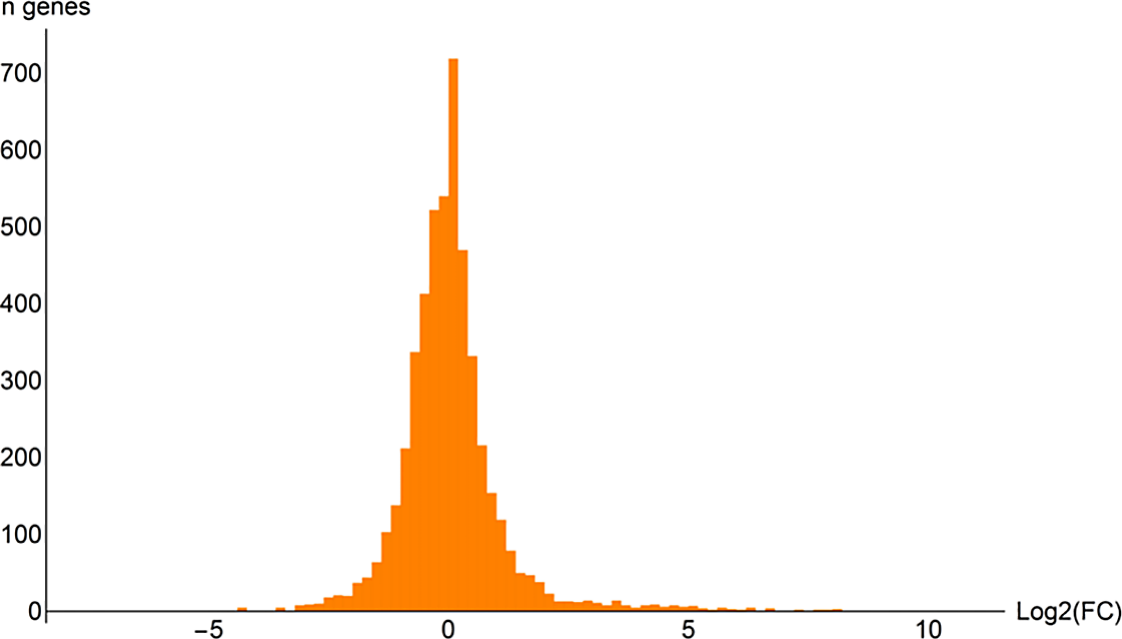
Histogram of RNA-seq log_2_(fold change) values. Statistical procedures were performed in CuffDiff.

In glucose medium, respiro-fermentative growth was observed, even though fully aerobic conditions were ensured by the small working volume in a large flask, vigorous shaking, and sampling at low OD_600_ values, with ethanol, glycerol and acetate as fermentation products. In xylose medium, fermentation products were absent, with an apparently purely respiratory metabolism. The maximum specific growth rate in glucose medium was approximately 0.8 h^-1^, while the maximum specific growth rate in xylose medium was approximately 0.35 h^-1^. All graphs and other renderings were generated using a new software suite for bioinformatics and integrated systems biology, termed Reactomica, which was developed in-house. Interactive datasets are provided in Computable Document Format (.CDF) as supplementary materials and are viewable using the free Wolfram CDF player, which can be downloaded from the Wolfram website [26], or by using Mathematica. High quality differential RNA-seq datasets were generated on the Illumina HiSeq platform to a high read depth. Figs 1 and 2 show the distribution of the data. Throughout, “up-regulated” refers to genes statistically up-regulated in a xylose medium compared to the condition with glucose as the carbon source, with a q-value below 0.05, as reported by CuffDiff (see ‘Materials and methods‘). Out of the total of 4 953 putative genes analysed, 329 were up-regulated and 251 down-regulated. Supplementary file S1 Table provides all expression values, differential expression statistics and UniProt annotations of all genes.

### The role of rich medium versus defined medium in the xylose response

We compared results from reference [3], which reported 36 genes in strain DMKU 3–1042 that were up-regulated only in the aerobic xylose-containing rich medium and not in any of the other three conditions tested (see S1 Table) (we refer to this gene set as the xylose-present/glucose absent unique gene set). Differential expression of 13 genes were correlated with the reference data (up-regulated in UFS-2791) and should be specific to the xylose response (or the absence of glucose), independent from the background medium, and present across strains. Notably, POT1, POX1 and FOX2 of peroxisomal β-oxidation were dramatically up-regulated in both datasets. Carnitine O-acetyltransferase (CAT2), associated with inter-compartmental transport of fatty acids, was also strongly up-regulated in both sets, supporting increased capacity for lipid catabolism (see a detailed discussion later). ICL2 and CIT3 of the 2-methylcitrate cycle was also strongly up-regulated in both experiments (see a detailed discussion later). Aldehyde dehydrogenase (ALD4), glucokinase 1 (GLK1) and dicarboxylic amino acid permease (DIP5) are the other enzymes functioning in central metabolism. Two regulatory proteins Ty transcription activator (TEC1) and the G2/mitotic-specific cyclin-4 (CLB4) were also among these. The rest of the genes in this list are not well-characterised. These include stationary phase protein 4 (SPG4), putative metabolite transport protein ywtG and uncharacterized membrane protein YMR155W.

Thirteen genes were uncorrelated with the reference data (constitutive in UFS-2791) (YPR011C, HSP12, YHL008C, POP6, NCE103, YLL032C, NCS2, GAS1, TOS1, PDR5, MYO1, EIS1, YDR134C) and are thus specific to either the strain or the rich medium. Most of these are uncharacterised proteins.

Finally, two genes were anti-correlated with the reference data (down-regulated in UFS-2791). These are the ammonia transport outward protein 3 (ATO3) and dihydroxyacetone kinase 1 (DAK1). The exact functions of ATO1, ATO2 and ATO3 are not currently known, apart from their possible involvement in mitochondrial retrograde signalling and ammonia production during starvation. It seems that ATO3 requires rich medium containing amino acids to be up-regulated, as was also observed in *S*. *cerevisiae* [27, 28]. In summary, several genes were highlighted as up-regulated both in our data and the xylose-present/glucose absent unique gene set from reference [3], in particular, the peroxisomal beta-oxidation and the supporting fatty acyl transporters. The roles of a number of other genes are unclear from this preliminary analysis, however.

### Gene Ontology

**Fig 3:**
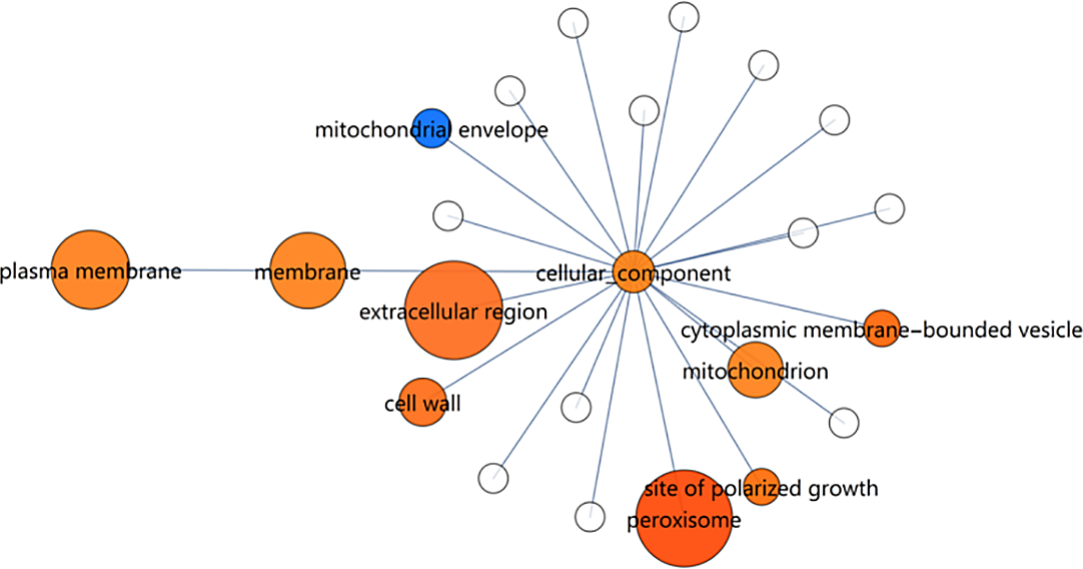
Gene set enrichment map of RNA-seq data using the ‘GO_slim’ yeast ‘cellular_component’ ontology. Size indicates the enrichment score of a gene set. Colour indicates the up/down direction of regulation: Red, up; blue, down. Brightness rises with the fraction of genes that are regulated in the dominant direction (up or down). Only nodes larger or equal than ‘cell wall’ are significant.

A total of 1 611 GO terms were included in the UniProt annotations of all proteins in the genome (see Materials and methods). To obtain an initial overview, the concise ‘GO_slim’ yeast from the Gene Ontology Consortium was used. GO term enrichment was performed separately for ‘cellular_component’, ‘molecular_function’ and ‘biological_process’ components of ‘GO_slim’ and the enrichment scores were used to render maps (see ‘Materials and methods‘). There is currently no generally accepted cutoff for interpreting significance of an enrichment value [14]. For the ‘cellular_component’ ontology, assuming a score cut-off at 1.64 (p = 0.05), five terms were considered to be significantly enriched (Fig 3 and S2 Table). These are ‘extracellular region’, ‘peroxisome’, ‘plasma membrane’, ‘membrane’, ‘mitochondrion’ and ‘cell wall’. ‘Plasma membrane’ is a subset of ‘membrane’ in the GO-slim ontology. It is striking that the peroxisomal genes were up-regulated (14 out of 32 genes) and only one down-regulated. The ‘extracellular region’ exhibited mostly up-regulation (22 out of 80 genes) with seven down-regulated genes. Of the 269 plasma membrane genes, 31 were up-regulated and 22 were down-regulated. In the mitochondrion the number of up and down-regulated genes were approximately equal (29 and 23, respectively out of 308 genes). The cell wall genes showed also mostly up-regulation, with 17 out of 80 genes up-regulated and four down-regulated.

**Fig 4:**
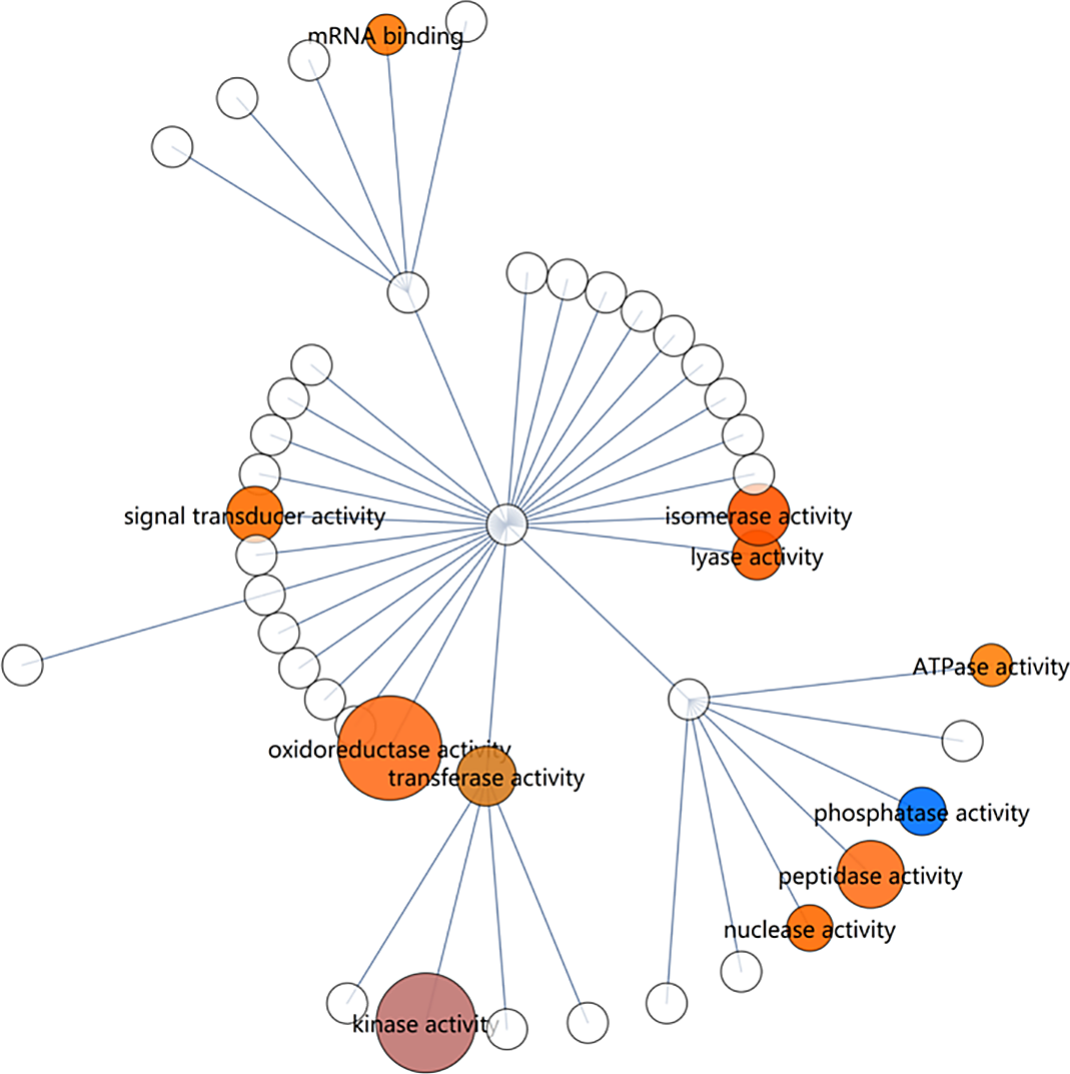
Gene set enrichment map of RNA-seq data using the ‘GO_slim’ yeast ‘molecular_function’ ontology. Size indicates the enrichment score of a gene set. Colour indicates the up/down direction of regulation: Red, up; blue, down. Brightness rises with the fraction of genes that are regulated in the dominant direction (up or down). Only nodes larger or equal than ‘peptidase activity’ are significant.

In the ‘molecular_function’ ontology, only three terms are regarded as significant. ‘Oxidoreductase activity’, ‘kinase activity’ and ‘peptidase activity’ (Fig 4 and S3 Table). ‘Oxidoreductase activity’ is the most highly enriched term in the ‘GO_slim’ gene sets (enrichment score = 4.49) and also in the complete GO enrichment (score = 13.2). It is notable that the redox balance, which is regulated by oxidoreductases, is one of the key considerations in the ability to utilize pentoses by yeasts, as it usually involves a requirement for NADPH and the additional generation of NADH, as should also be the case with *K*. *marxianus* since it does not possess a xylose isomerase.

**Fig 5:**
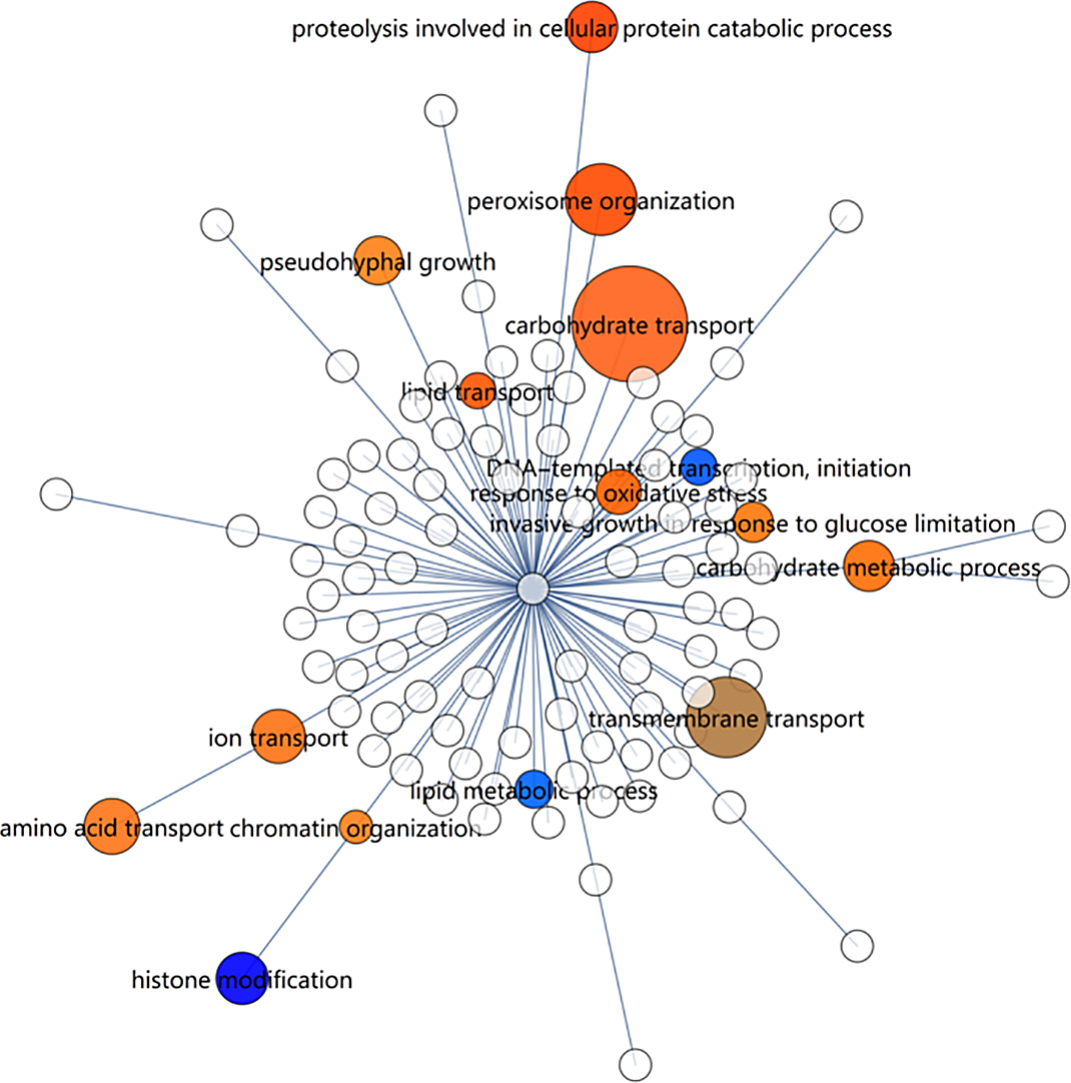
Gene set enrichment map of RNA-seq data using the ‘GO_slim’ yeast ‘biological_process’ ontology. Size indicates the enrichment score of a gene set. Colour indicates the up/down direction of regulation: Red, up; blue, down. Brightness rises with the fraction of genes that are regulated in the dominant direction (up or down). Only nodes larger or equal than ‘proteolysis involved in cellular protein catabolic process’ are significant.

The ‘biological_process’ ontology is rich in providing a framework for exploring the regulation of cellular processes in response to different carbon sources. The ‘carbohydrate transport’ gene set was the most significantly altered, with 21 out of 65 transporter genes up-regulated and 7 down-regulated (Fig 5 and S4 Table). The ‘transmembrane transport’ gene set displayed a more equal up vs down-regulation, whereas ‘peroxisomal organization’ genes had exclusively up-regulated genes. The latter feature is shared with the ‘peroxisome’ gene set in the ‘cellular_component’ ontology, indicative of a general up-regulation of peroxisomal components and activities. The ‘amino acid transport’ gene set also indicated differential expression. The gene set of ‘ion transport’ was also enriched, but is closely related to ‘amino acid transport’. Also significant is ‘carbohydrate metabolic process’, which is better interpreted in terms of metabolic pathways. Although ‘histone modification’ and ‘proteolysis involved in cellular protein catabolic process’ both have significant scores, they only have one and four genes, respectively, in the gene sets, and they were therefore not interpreted for further investigation.

In summary, peroxisomal organisation and metabolism were clearly and strongly up-regulated on xylose, consistent with a previous observation [3] that at least fatty acid β-oxidation was up-regulated in a complex medium that likely contained small amounts of lipids, which were absent in the present study where a chemically defined medium was used. Secondly, a strong effect was seen on carbohydrate transporters, which was consistent with the experimental setting. Some of these differentially expressed putative sugar transporter genes, such as the various *HGT1* homologs of *K*. *lactis*, were up-regulated as high as 1242-fold (see S4 Table). Some of these may encode for xylose transporters. As sugar transporters are highly similar, additional annotation of this group is required before more conclusions regarding sugar transport may be drawn. Thirdly, the extracellular region was affected. These are proteins that are secreted into the medium, such as lytic enzymes or receptors that sense a new environment, as well as the mating genes. Some of these gene sets are explored in more detail in the supplementary text S1 Text.

It is interesting to note that although many genes in the mitochondrion were differentially regulated, these genes are not the main enzymes of the TCA cycle, electron transport or oxidative phosphorylation. Thus, there is no response suggesting adaptation to a change in the internal energy charge. As the maximum specific growth rate in xylose medium was less than half of that in glucose medium, whereas the cell sizes were comparable, one might expect differential regulation of cell cycle progression genes, or even biomass formation-specific genes like those encoding for ribosomal proteins. There was a notable absence of such gene sets in the enrichment analysis. This is also reasonable since cell cycle control signalling proteins are mostly kinases, which are controlled by post-translational modifications. Most genetic responses observed in the data thus deal with utilisation of alternative substrates. Further, there was only a weak response in the oxidative stress genes, as was expected from aerobic cultivation. From the genes annotated with ‘response to oxidative stress’ (PRDX5, CTT1, CCP1, YFH1, DCS1, HMX1, SVF1, YDR222W), only peroxiredoxin-5 (PRDX5, mitochondrion, cytoplasm, peroxisome, 3.9-fold up-regulated) and catalase T (CTT1, cytoplasm, 2.4-fold up-regulated) were moderately differentially regulated, resulting in an enrichment score of 1.19, which was insignificant against the background enrichment.

The data indicated several interesting aspects regarding sugar utilisation, including up-regulation of pathways for utilisation of galactose, xylose and arabinose (see S5). Several enzymes with β-glucosidase activity were found in the annotation (see S1 Text), whereas only one enzyme was both extracellular and significantly up-regulated (48.8-fold), namely cellobiase (EC 3.2.1.21). Cellobiase is not a typical cellulase that can depolymerise cellulose, as it functions to hydrolyse the disaccharide cellobiose. *K*. *marxianus* UFS-Y2791 also does not possess typical secreted xylanases, proteases, peptidases or lipases, which would allow a microorganism to thrive on plant matter or even attack a live plant. Instead, the strain possesses the inulinase gene *INU1* which hydrolyses the fructan inulin or the disaccharide sucrose. The *INU1* gene was dramatically up-regulated 91-fold and abundantly expressed in xylose medium.

The pheromone signalling system involved in sexual reproduction, as well as several other genes involved in the conjugation process as well as in invasive growth, were also up-regulated (see ‘extracellular region’ in S2 Table). In the presence of xylose (or absence of glucose) there is thus a response to physiologically adapt to an invasive lifestyle and utilise other sources of nutrients (xylose, arabinose, inulin, cellobiose and amino acids), as would be found in the natural plant environment. A long-term survival strategy (sexual reproduction) is also activated in the more nutrient-poor condition.

Most metabolic pathways for amino acid synthesis were down-regulated, as was expected at the lower growth rate; μ_max_ values of 0.8 and 0.35 were recorded on glucose and xylose, respectively. The well-known importers of ammonia were constitutive (MEP3) or down-regulated (MEP2, 2.8-fold).

### Global metabolic response elucidated by pathway-to-pathway networks

**Fig 6:**
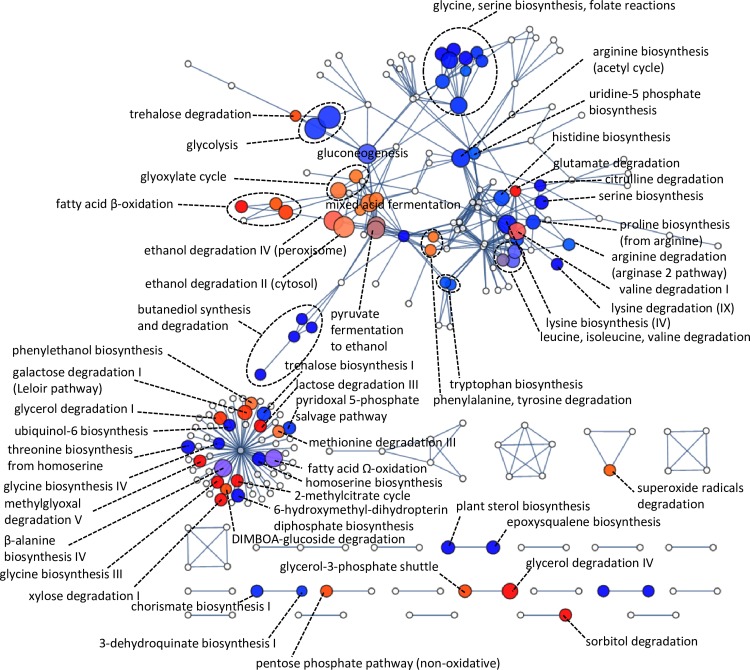
Gene set enrichment map of RNA-seq data using the pathway-to-pathway network. Size indicates the enrichment score of the gene set representing each pathway. Colour indicates the up/down direction of regulation: Red, up; blue, down. Brightness indicates uni-directionality of regulation. The ‘Self’ cluster on bottom-left includes pathways not mapped to other pathways since their intersections scores were below a threshold.

To capture the global metabolic response to growth on xylose in a single view, an innovative pathway-to-pathway network was constructed by clustering pathways together by their common metabolites (Fig 6, S1 Network and S1 Table). The wide down-regulatory profile of amino acid metabolic pathways is visible, where all amino acid biosynthetic pathways were shut down, with only glycine biosynthesis III having some element of up-regulation. Similarly, most amino acid catabolic pathways were shut down with only glutamate, valine, tyrosine, phenylalanine, tryptophan and methionine degradation III having some up-regulated genes.

Fatty acid oxidation, peroxisomal ethanol degradation and mixed acid fermentation, which cluster together, were mostly up-regulated. Close by in the network is glycolysis, which was down-regulated. A moderately increased capacity in the glycerol-3-phosphate shuttle was evident here, with the mitochondrial glycerol-3-phosphate dehydrogenase *GUT2* gene 4.4-fold up-regulated. Note that the up-regulated glycerol-3-phosphate shuttle functions independently from down-regulated glycerol production.

### Reporter metabolites

Reporter metabolite enrichment was performed to reveal those metabolites around which significant differential expression of enzymes took place. These compounds could be interpreted as those from which there was *(a)* a marked change in capacity for their utilisation or production between conditions, *(b)* to have required a significant degree of regulation by their neighbouring enzymes to establish homeostasis in the different environment, or *(c)* to have a different concentration predicted between conditions where it may be a candidate as a signalling molecule. The top eight reporter metabolites of highest enrichment were 3-phosphoglycerate, acetaldehyde, NADH, glyceraldehyde-3-phosphate, β-alanine, NAD^+^, glycerate and ethanol, in this order (S1 Table). NADH, NAD^+^, acetaldehyde and ethanol are involved in redox metabolism and thus support the high gene set enrichment score of the GO term ‘oxidoreductase activity’ (GO:0016491). It was interesting to note that the enrichment score of ATP was among the lowest against the background and considered insignificant. Also, glutathione and its reduced form, which are known to be involved in redox metabolism, were not significantly enriched, suggesting no severe oxidative stress. Oxidative stress in *K*. *marxianus* is seemingly more important under oxygen limiting conditions, as imposed by static and high-temperature conditions [3].

### Molecular networks

**Fig 7:**
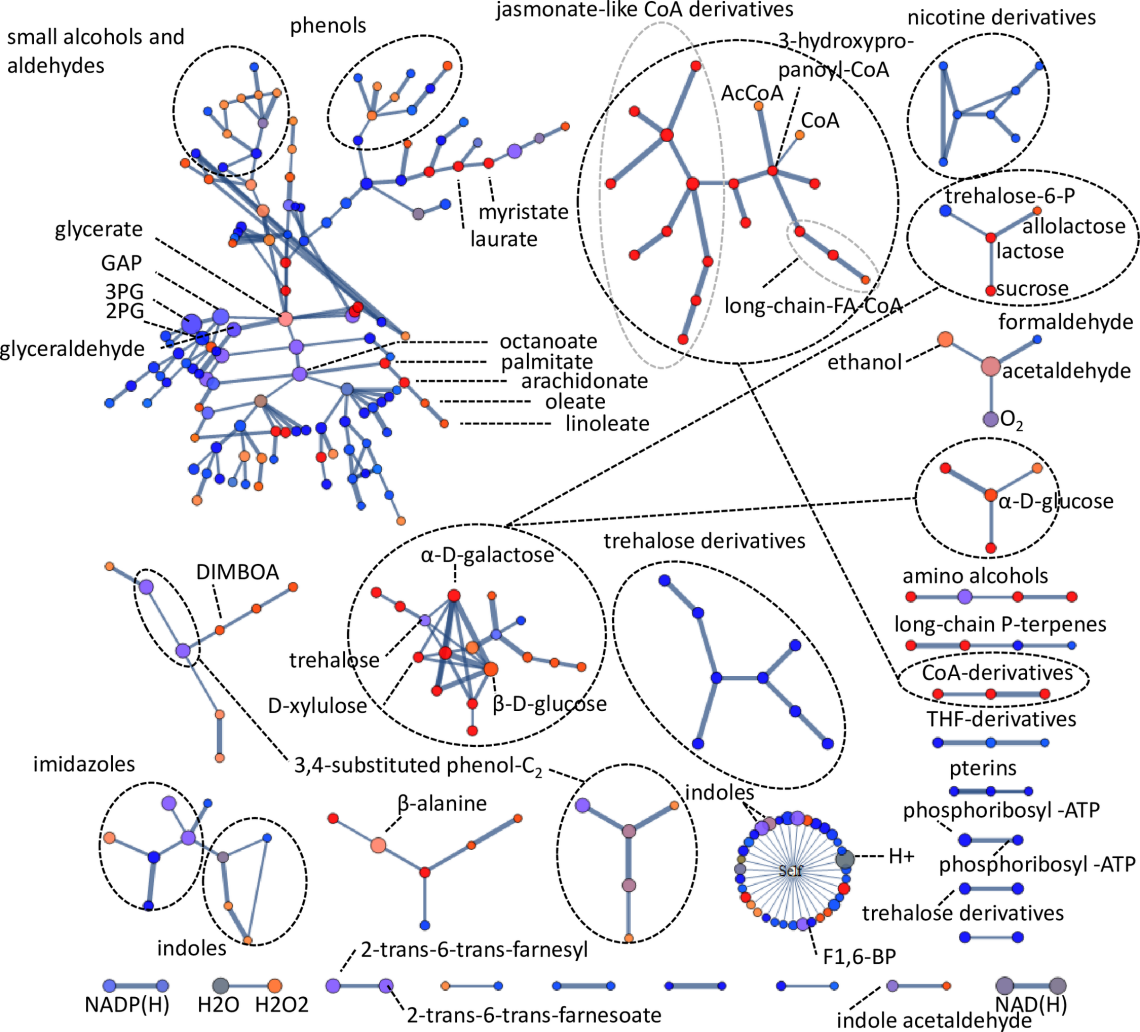
A molecular network of all reporter metabolites enriched at or above enrichment score > = 1.64 (p = 0.05). Mapping between compounds was performed with a local string matching procedure based on the SMILES string of each compound. Scores were normalised to the string size for the longest of the two molecules in a pairwise comparison. Edge weights represent normalised similarity scores. The “Self” node maps all compounds with an insufficient normalised similarity score to other compounds (<0.4). Size indicates the enrichment score of a gene set. Colour indicates the up/down direction of regulation: Red, up; blue, down. Brightness indicates uni-directionality of regulation.

To determine whether the reporter metabolites represented any distinct molecular structural groups, a molecular network of the reporter metabolites was reconstructed using a simple molecular structure similarity matching protocol. This approach could identify some co-regulated groups of structurally related molecules that were not evident from pathways-based analyses. Fig 7 shows a number of clusters of enriched reporter metabolites grouped by their molecular structures (see interactive file S2 Network for molecular structures and annotations).

Groupings of CoA-conjugates, long-chain and short-chain fatty acids are representative of increased catabolic activity of β-oxidation and activation steps. Up-regulated sugar clusters corresponded to those found in reporter metabolite-enzyme networks, but were represented in a clearer fashion. Noteworthy is that effectors of trehalose containing sugars and sugar lipids were down-regulated. The three-carbon molecules in central carbon metabolism, which are close together in the network including 3-phosphoglycerate, 2-phosphoglycerate, glyceraldehyde-3-phosphate and glyceraldehyde, were strongly enriched and their effectors down-regulated. This mapping is useful as a concise representation of potentially all metabolites in a cell, grouped by their structures. The method would especially be useful for visualising metabolomics datasets of which the link to reactions and pathways is not yet clear. A better separation of clusters is obtained in mapping larger metabolites as found in secondary metabolism.

### Key enzymes that may affect metabolite pools

**Fig 8:**
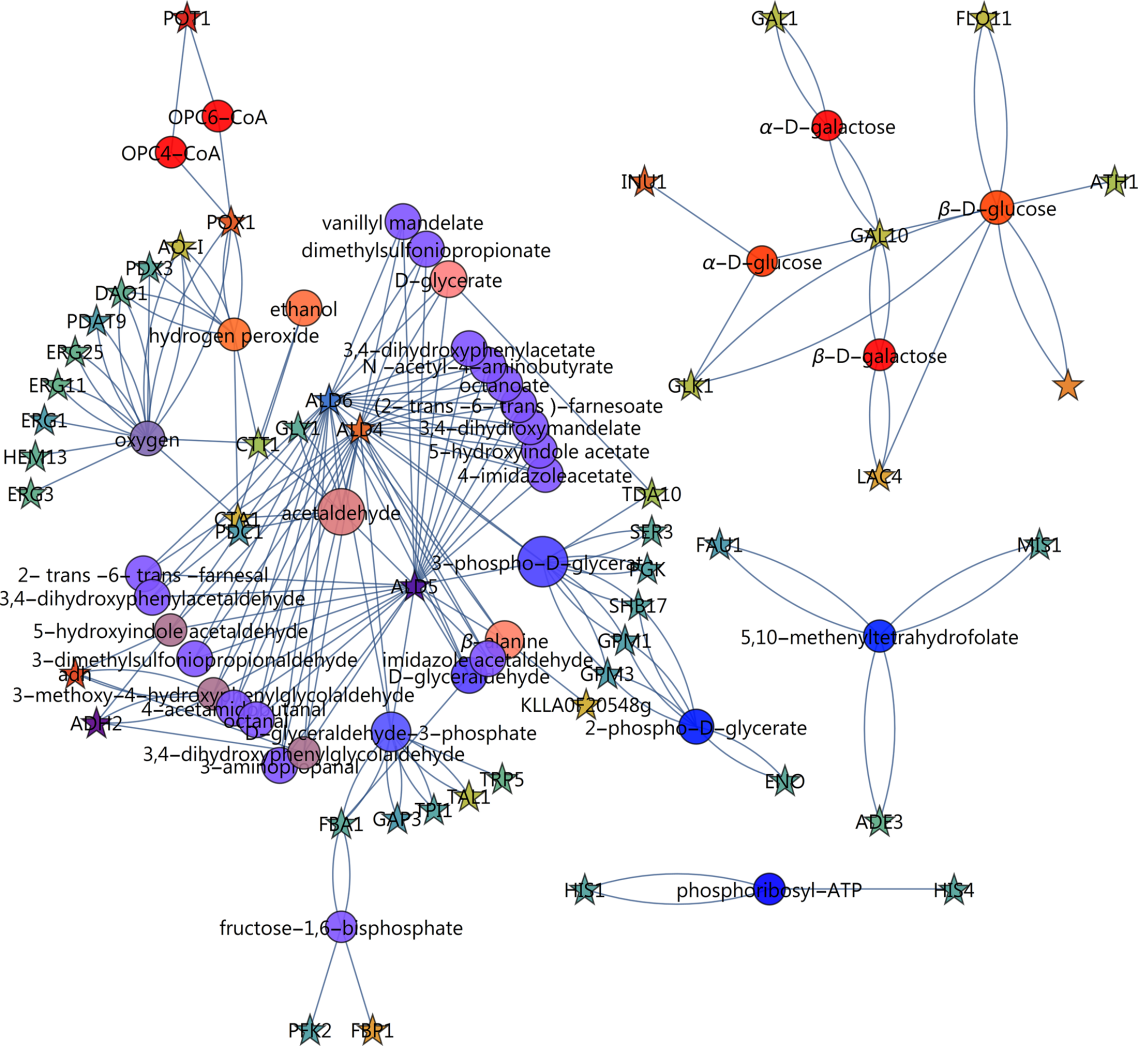
Reporter metabolite-enzyme network, capturing enriched reporter metabolites and the enzymes that affect them. Only reporter metabolites with enrichment values above 3.0 were included and only the differentially expressed genes from RNA-seq with q-values (corrected p-values) below 0.05. For reporter metabolites (circles), size indicates the enrichment score of a gene set and colour indicates the up/down direction of regulation. Brightness indicates uni-directionality of regulation. For enzymes (stars), colour indicates the up/down direction of regulation based on the log_2_(fold change) scheme. Reporter metabolite enrichment values are represented in S1 Table. (For full information on gene names, the interactive file S3 Network or the corresponding annotations in S1 Table, using the “Gene names (primary)” column, may be consulted.)

Combining both the reporter metabolites and the enzymes by which they are regulated into an enzyme-reporter metabolite network, effectively reveals the hotspots of metabolic regulation as well as the key players in regulation. Including all interactions with enriched compounds (enrichment score > 1.64, q < 0.05) was not feasible for a detailed investigation (1352 interactions). Instead, we extracted the interactions containing nodes with enrichment scores above 3.0 (Fig 8, see interactive file S3 Network).

**Fig 9:**
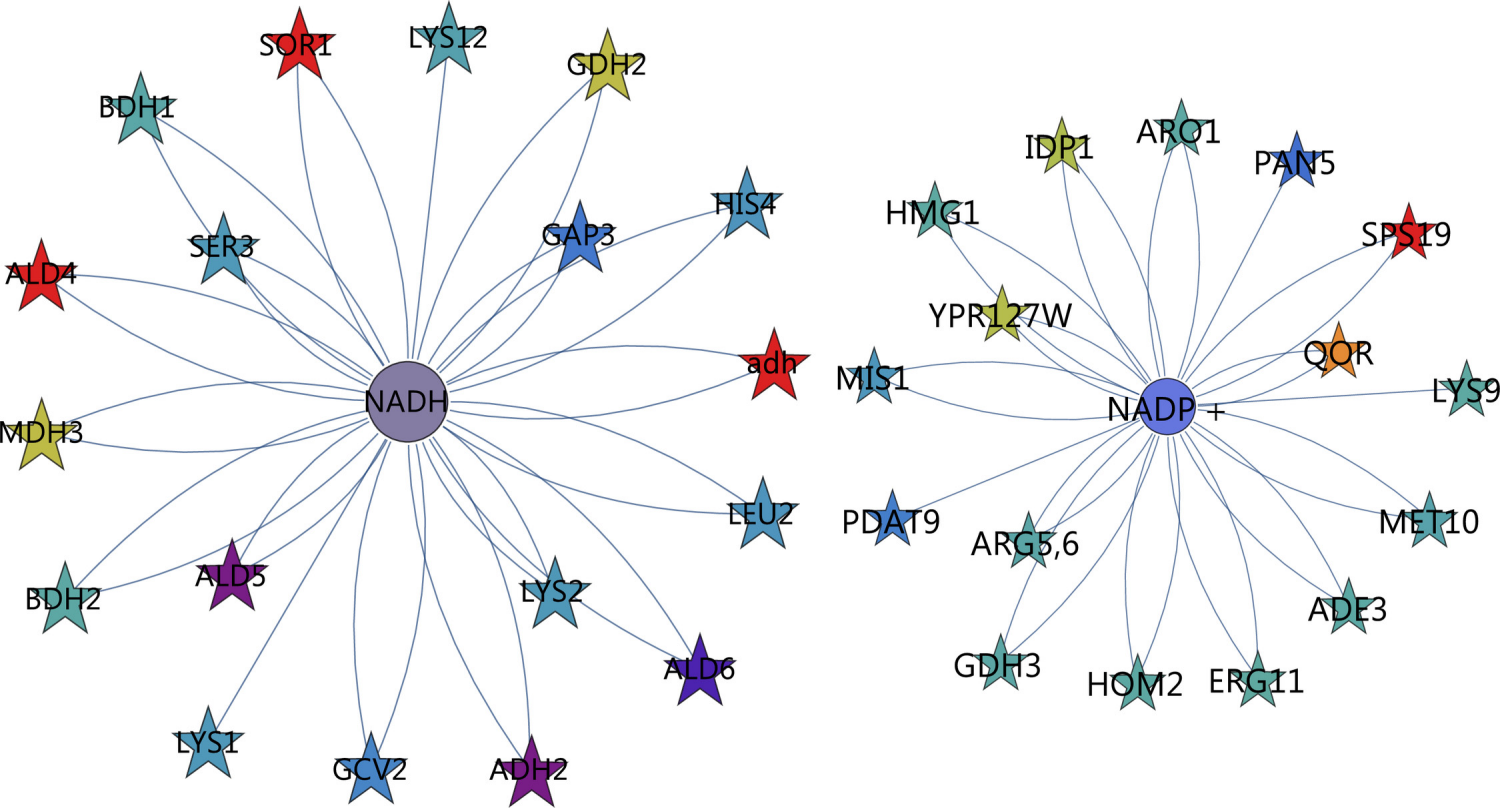
Enzyme-metabolite interaction network around redox cofactors. Left: NADH; Right: NADP^+^. For reporter metabolites (circles), size indicates the enrichment score of a gene set and colour indicates the up/down direction of regulation. Brightness indicates uni-directionality of regulation. For enzymes (stars), colour indicates the up/down direction of regulation based on the log_2_(fold change) scheme. (For full information on gene names, the interactive file S4 Network or the corresponding annotations in S1 Table, using the “Gene names (primary)” column, may be consulted.)

A major network is evident in which NAD^+^/NADH, oxygen and the aldehyde dehydrogenase have a strong involvement. A second subnetwork involves sugar metabolism and glycosylation. A third revolves around one-carbon metabolism. The subnetwork of NAD^+^ (enrichment score = 4.2) was extracted (Fig 9, left, see interactive file S4 Network), which reveals a number of genes involved with biosynthesis being down-regulated. In addition, a large contribution to the enrichment score is made by the aldehyde dehydrogenases *ALD4*, *ALD5* and *ALD6*, by the alcohol dehydrogenases annotated as *ADH2* and *ald*, and by sorbitol dehydrogenase *SOR1*.

Since *K*. *marxianus* relies on an NADPH dependent xylose reductase, it was expected that NADPH would be enriched (enrichment score = 2.6). The enzymes directly affecting NADPH are explored in Fig 9 (right, see interactive file S5 Network). We anticipated up-regulation of major NADPH producing enzymes to supply reducing power for xylose utilisation by xylose reductase. Instead, only three enzymes directly involved with NADP(H) were up-regulated. *IDP1* (mitochondrial NADP-specific isocitrate dehydrogenase) was only moderately up-regulated (2.2-fold). A more significantly up-regulated enzyme, mitochondrial quinone oxidoreductase (*QOR*, 9.9-fold up-regulated) reduces 1,4-benzoquinone, as was shown by cloning this gene from *K*. *marxianus* in *E*. *coli* [29]. It is not part of the major catabolic processes in central metabolism, however. Another is *YPR127W*, a putative pyridoxal reductase that functions to degrade vitamin B6 and involved with multidrug resistance and not central metabolism [30]. In the oxidative pentose phosphate pathway, which is assumed to be the main generator of NADPH in most species, *GND1* (6-phosphogluconate dehydrogenase) and *SOL1* (6-phosphogluconolactonase) were, however, constitutively expressed. Another enzyme, *SPS19* (peroxisomal 2,4-dienoyl-CoA reductase), was strongly up-regulated at 269-fold. It functions to reduce double bonds to facilitate β-oxidation of unsaturated fatty acids in the peroxisome [31], another indication that peroxisomal metabolism was strongly differentially regulated.

### Central carbon metabolism

**Fig 10:**
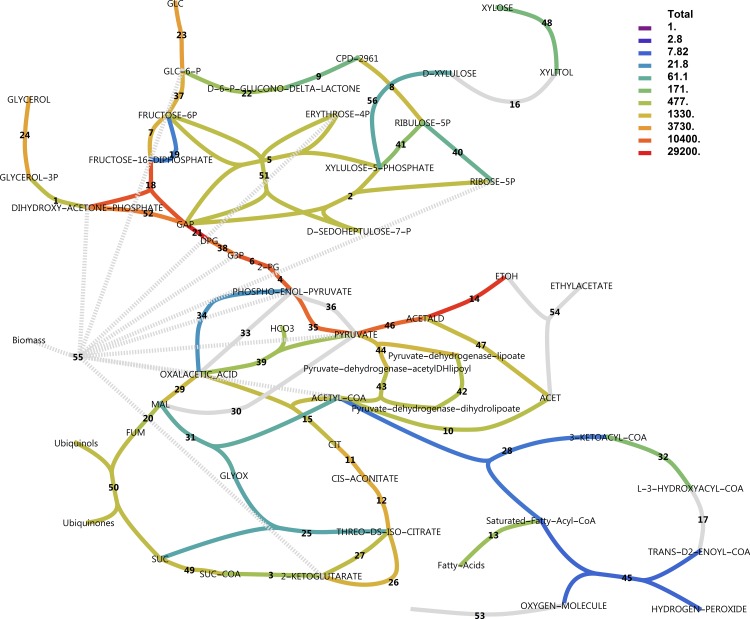
Total transcript levels in central metabolic pathways with glucose as the carbon source. Transcript levels for all genes catalysing a reaction were summed. Note the logarithmic scale. Dark grey indicates genes present and constitutively expressed. Light grey indicates genes not found in annotation or combined reactions. For reaction names, see S1 Pathway.

**Fig 11:**
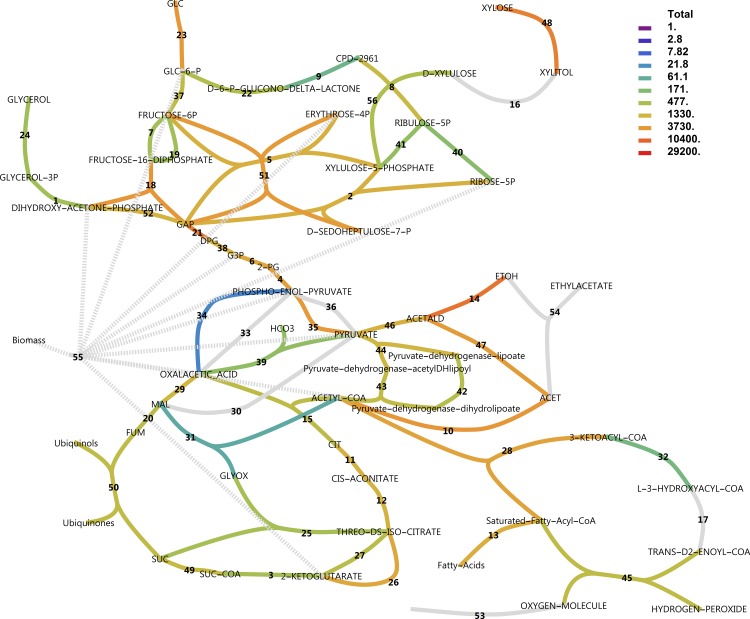
Total transcript levels in central metabolic pathways with xylose as the carbon source. For reaction names, see S1 Pathway.

To explore the metabolic response of the central carbon metabolism, metabolic pathway maps were created from MetaCyc pathways and RNA-seq data were mapped using various colouring schemes. Figs 10 and 11 show total transcript abundances mapped to reactions (see also S1 Pathway). It is evident that on glucose, the central glycolytic route from glucose to ethanol is highly expressed. In xylose medium, transcript abundance is less pronounced in glycolysis and ethanol production, whereas PPP, the pyruvate dehydrogenase bypass and β-oxidation enzymes increase in transcript abundance.

**Fig 12:**
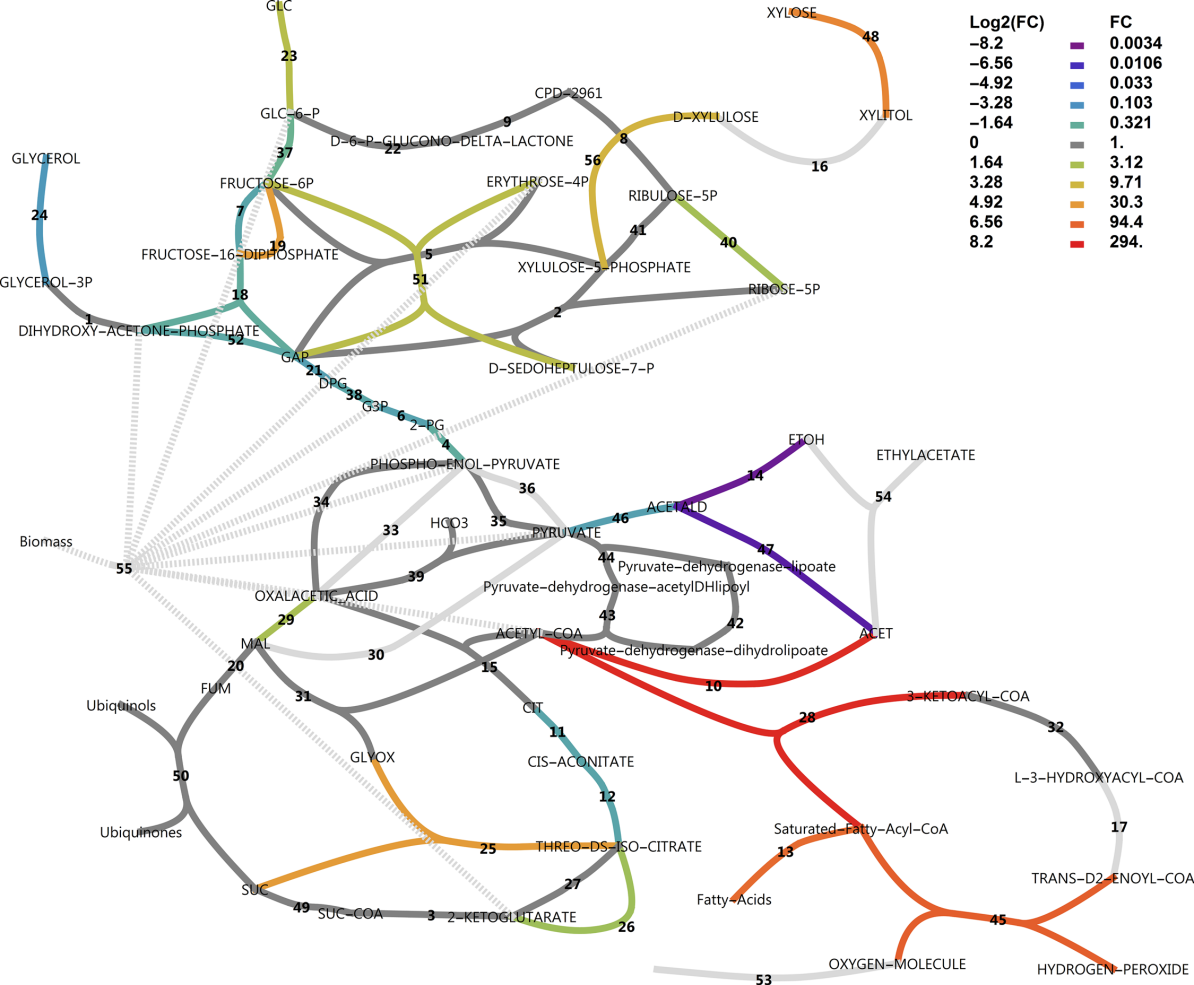
Uncompartmentalised response to xylose in central carbon metabolism in a log_2_(fold change) scheme. A log_2_(fold change) is defined as the log_2_ ratio of transcripts on xylose divided by that on glucose, as reported by CuffDiff. Reactions vETC, vEthylAcetate and vGrowth were manually added to the model. In the case of more than one enzyme that could perform the same function, the largest fold change in expression was used for the colour rendering. For reaction names, see S1 Pathway.

An interesting differential expression pattern is evident in Fig 12. Consistent with the experimental setting, the NADPH-dependent D-xylose reductase gene *XYL1* was drastically up-regulated on xylose (48.8-fold) with very high gene expression levels on xylose. Xylitol dehydrogenase was absent from the annotation. However, is has been demonstrated that sorbitol dehydrogenase *SOR1* can act as a xylitol dehydrogenase in *S*. *cerevisiae* [32]. Significant up-regulation of *SOR1* by 208-fold supports this function. Xylulokinase *XKS1* was also 11.3-fold up-regulated. Transaldolase *TAL1* of the non-oxidative pentose phosphate pathway (PPP) and ribose-5-phosphate isomerase *RKI1* were moderately up-regulated (4.8 and 2.6-fold). We did, however, not observe up-regulation of any enzymes in the oxidative branch to support additional NAPDH production for xylose utilisation, or to combat oxidative stress by charging of the glutathione system. We found a remarkably clear down-regulation of glycolytic genes on xylose with a moderate fold change. The gluconeogenesis-associated gene fructose-1,6-bisphosphatase *FBP1* was also sharply up-regulated by 27-fold, which was also observed in a microarray experiment of a xylose utilising recombinant *S*. *cerevisiae* strain [33]. β-Oxidation reactions and fatty acid activation reactions were clearly up-regulated.

**Fig 13:**
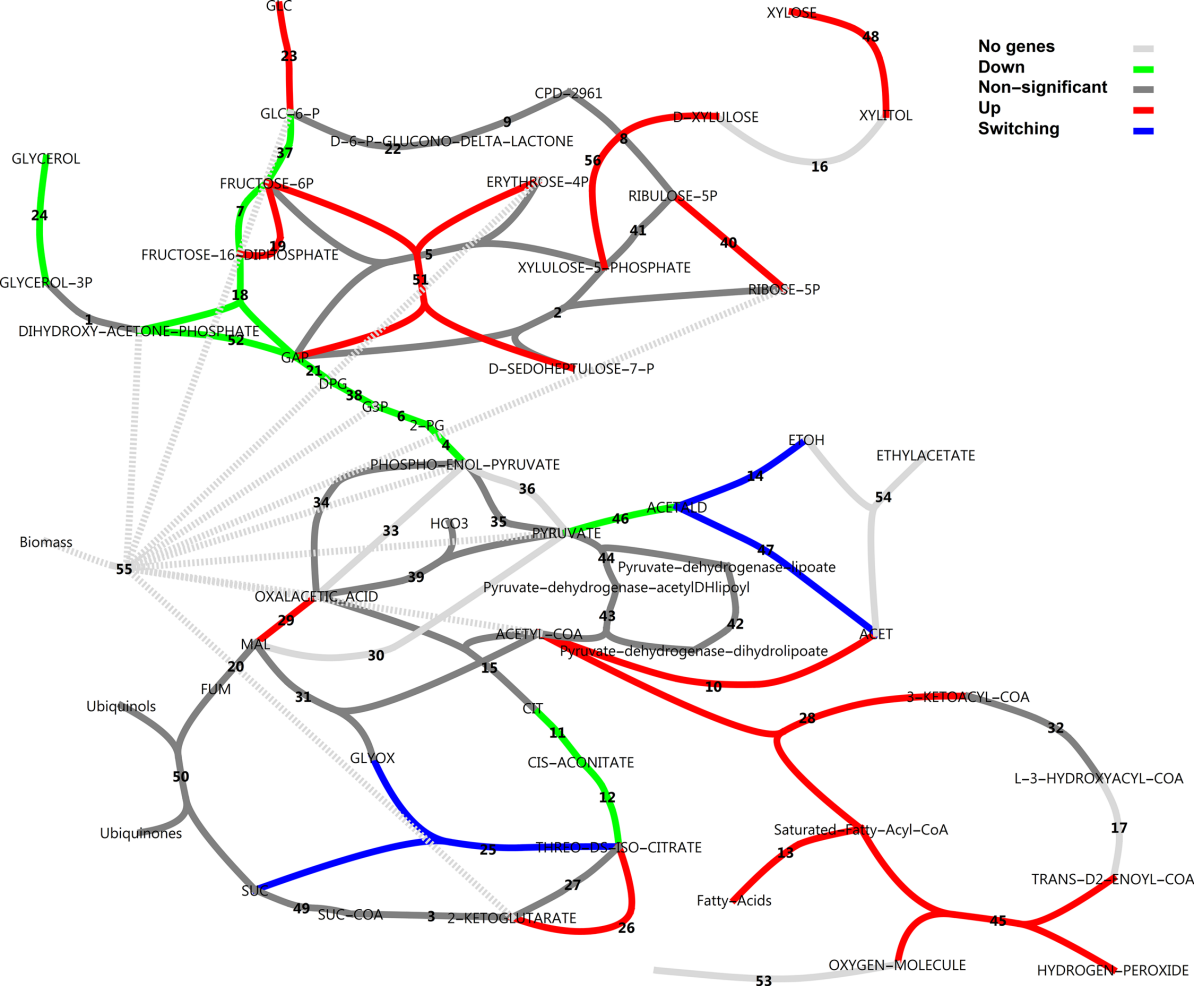
Uncompartmentalised response to xylose in central carbon metabolism as a classification scheme. Blue reactions represent those for which more than one enzyme gene has been assigned and for which some were up-regulated and some down-regulated, referred to as isozyme switching. For reaction names, see S1 Pathway.

Further, there was also a strong apparent down-regulatory effect on alcohol dehydrogenases and aldehyde dehydrogenases, and both the citrate synthase of the TCA cycle and the isocitrate lyase of the glyoxylate cycle were seemingly dramatically up-regulated. The latter two observations in this analysis, however, are misguided by the lack of compartmentalisation of the response. It should be noted that many metabolic reactions could be catalysed by more than one enzyme, where for some reactions like those catalysed by alcohol dehydrogenase (ADH), there are at least five gene products that could potentially catalyse the same reaction. Fig 12 used only the most extremely altered differentially expressed gene for rendering. Fig 13 provides a different perspective, however, where reactions that have more than one associated gene and which were regulated in opposite directions are identified. These are isocitrate lyase, aldehyde dehydrogenase and alcohol dehydrogenase.

**Fig 14:**
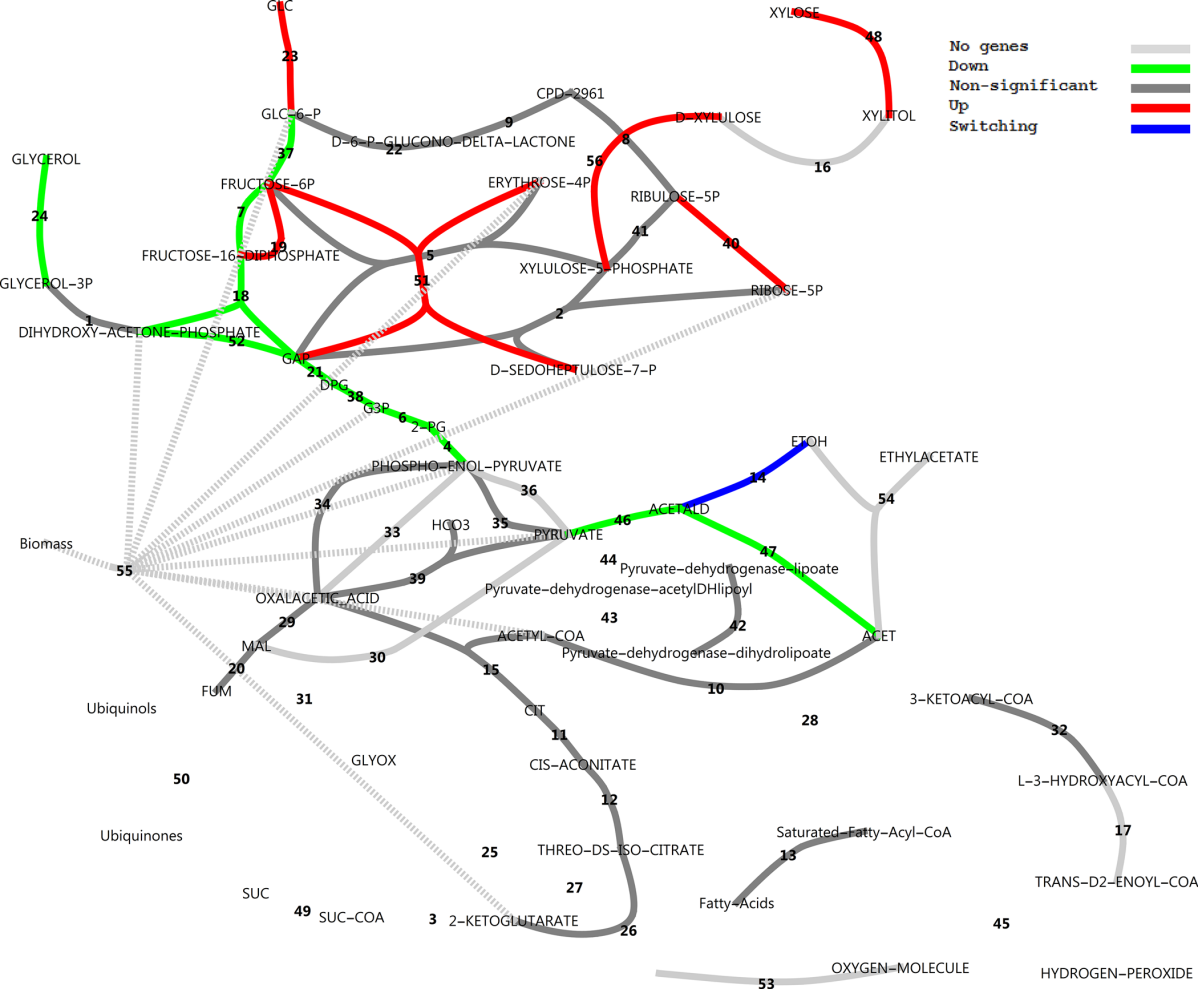
Compartmentalised response to xylose in central metabolism in the cytoplasm using the classification scheme. Blue reactions represent those for which more than one enzyme gene has been assigned and for which some were up-regulated and some down-regulated, referred to as isozyme switching. For reaction names, see S1 Pathway.

Subsequently, the GO ‘cellular_component’ terms from the UniProt_SwissProt_fungi were used to render compartmentalised maps reconstructed from MetaCyc pathways. Fig 14 shows that in the cytosol xylose utilisation reactions were up-regulated, whereas glycolysis, together with pyruvate decarboxylase, NAD^+^-specific acetaldehyde dehydrogenase (ALD) and glycerol production were down-regulated. Further, a number of reactions usually associated with the TCA cycle were also present in the cytosol and were constitutively expressed. More than one type of ADH with opposite regulatory direction is present in the cytosol. In the peroxisomal compartment (see S1 Pathway), β-oxidation of lipids, which in *S*. *cerevisiae* is performed exclusively in the peroxisomes [34], is clearly visible with only the 3-hydroxyacyl-CoA dehydrogenase (OHACYL-DEHYDROG-RXN) gene missing from the annotation. In mitochondria (see S1 Pathway) the reactions catalysed by the ALDs (*ALD4*, *ALD5*, *ALD6*) and ADHs were still isozyme switching reactions. Both *ALD4* (up-regulated 83-fold) and *ALD5* (down-regulated 167-fold) were strongly differentially expressed. Several ADH genes were found in *K*. *marxianus*. Two of these were annotated as *ADH3* and *ADH4*, both mitochondrial, whereas five (*ADH1*, *ADH2*, *ADH6*, *SFA1* and *adh*) were taken to be cytoplasmic. As previously reported [35], ADH2 was only expressed significantly in the presence of glucose, and was in fact the most significantly down-regulated gene in our dataset (229-fold down-regulated). This corresponds to the regulation of the ADH2 orthologs in *S*. *cerevisiae* and *K*. *lactis* [35]. Conversely, ADH1 was constitutively expressed, again as previously reported [35], which differs from the regulation in *S*. *cerevisiae* and *K*. *lactis* where glucose stimulated ADH1 expression. Transcriptional rewiring of ADH isozymes is thus present in *K*. *marxianus* compared to its relatives.

**Fig 15:**
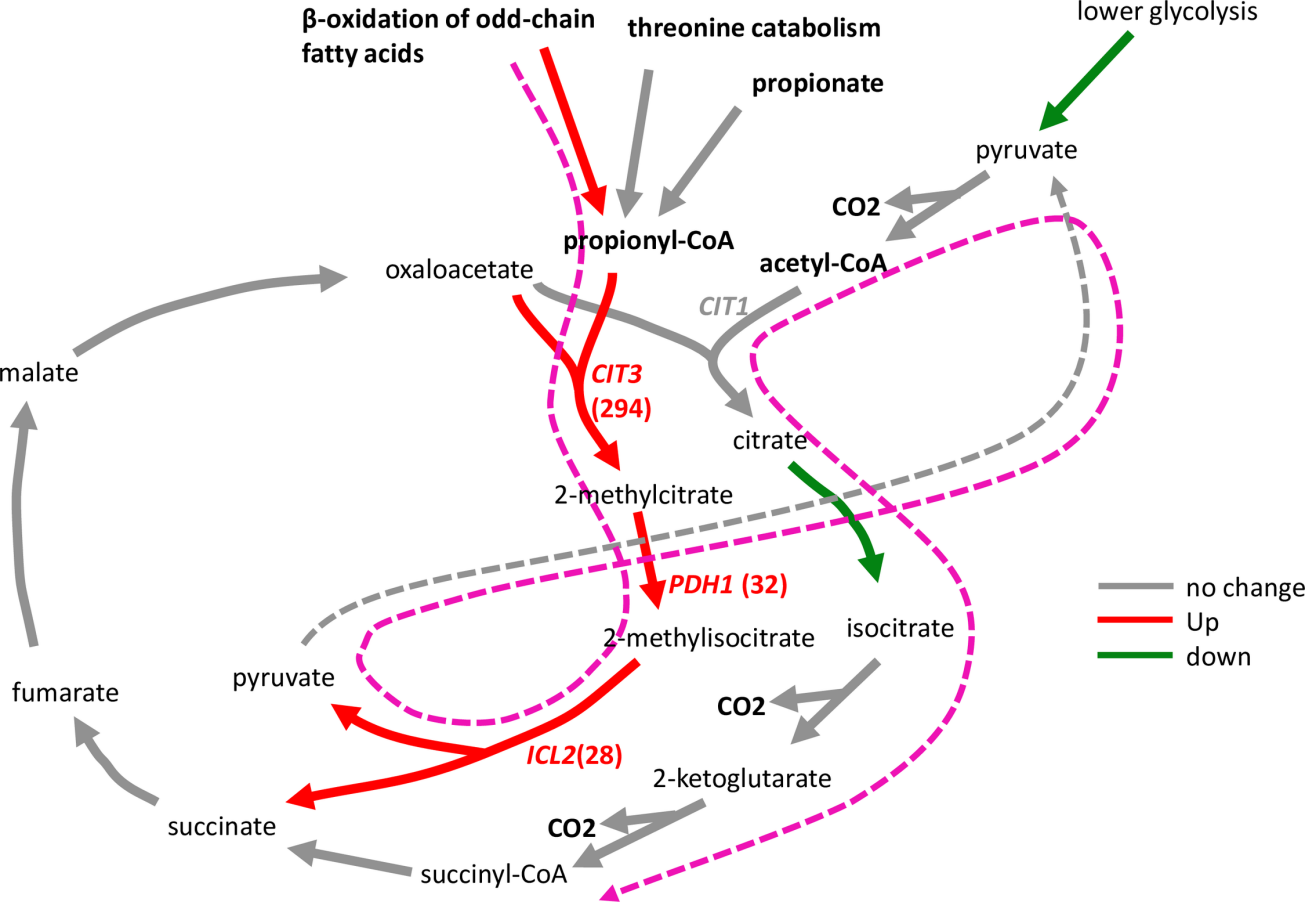
2-Methylcitrate pathway. The suggested route of three-carbon units is indicated in magenta.

In the mitochondrial map, the NADPH specific isocitrate dehydrogenase gene was up-regulated, whereas citrate synthase of the TCA cycle and isocitrate lyase were seemingly up-regulated. Surprisingly, the glyoxysome map revealed that the glyoxylate cycle specific isocitrate lyase (*ICL1*) was down-regulated. Upon further investigation, the isozyme present in the mitochondrion, which was mapped to the TCA cycle by the PathoLogic algorithm using Kegg-Kaas annotations, was in fact *ICL2*, the 2-methylisocitrate lyase, perhaps confusingly termed *ICL2*. Although *ICL1* and *ICL2* share a high sequence similarity, the latter does not use isocitrate as a substrate and does not produce glyoxylate but uses 2-methylisocitrate instead to produce succinate and pyruvate [36]. The gene product from *ICL2* is part of the propionyl-coezyme A pathway, otherwise known as the 2-methylcitrate pathway [37]. The pathway starts with *CIT3* (*YNR001C*), the mitochondrial enzyme that condenses oxaloacetate with propionyl coenzyme A to form 2-methylcitrate. Indeed, the citrate synthase that was up-regulated (Fig 12) was not the *CIT1* gene (*YPR001W*) that is both cytosolic and mitochondrial and associated with the TCA cycle, but instead the mitochondrial *CIT3*. Further inspection of the full GO ontology enrichment set revealed that all three key genes in the 2-methylcitrate pathway were significantly up-regulated. The strength of the response in the 2-methylcitrate pathway is given in Table 1, where *CIT3*, *PDH1* and *ICL2* were up-regulated 294, 32 and 28-fold, respectively, and Fig 15 shows the connection of this pathway with the TCA cycle. The GO term ‘propionate catabolic process’ (GO:0019543) was found to be significantly enriched with an enrichment score of 5.2. Apart from the last cycle of β-oxidation of odd-chain fatty acids in the peroxisomes as the source of propionyl-CoA, the latter could be derived from propionate or even from threonine breakdown [36]. In the gene set for ‘threonine metabolic process’ (GO:0006566), only one gene was up-regulated, namely the low specificity L-threonine aldolase (*GLY1*). Another gene was also annotated as *GLY1* and down-regulated. Thus, there is no conclusive evidence that threonine catabolism was up-regulated. The up-regulation of the 2-methylcitrate pathway is, therefore, more likely for the catabolism of short-chain fatty acids and not threonine.

**Table 1:** Differential expression of the constituent genes mapped to the GO term ‘propionate catabolic process’ (GO:0019543) on glucose and xylose, respectively.

Id	Value (Glc)	Value (Xyl)	log2(FC)	qvalue	signt	Entry	Protein names	Gene names
GK5S-1542	5.2	1512.1	8.2	0.001	yes	P43635	Citrate synthase 3 (EC 2.3.3.16)	CIT3 YPR001W YP9723.01
GK5S-1543	27.5	901.5	5.0	0.001	yes	Q12428	Probable 2-methylcitrate dehydratase (EC 4.2.1.79)	PDH1 YPR002W LPZ2W YP9723.02
GK5S-2306	15.5	436.9	4.8	0.001	yes	Q12031	Mitochondrial 2-methylisocitrate lyase (EC 4.1.3.30)	ICL2 YPR006C LPZ6C YP9723.06C

Painted differential expression pathway maps were generated for all of 235 metabolic pathways found in the annotation. They can be explored in see S2 Pathway.

### Metabolic Regulation Analysis to dissect hierarchical and metabolic levels of regulation

**Fig 16:**
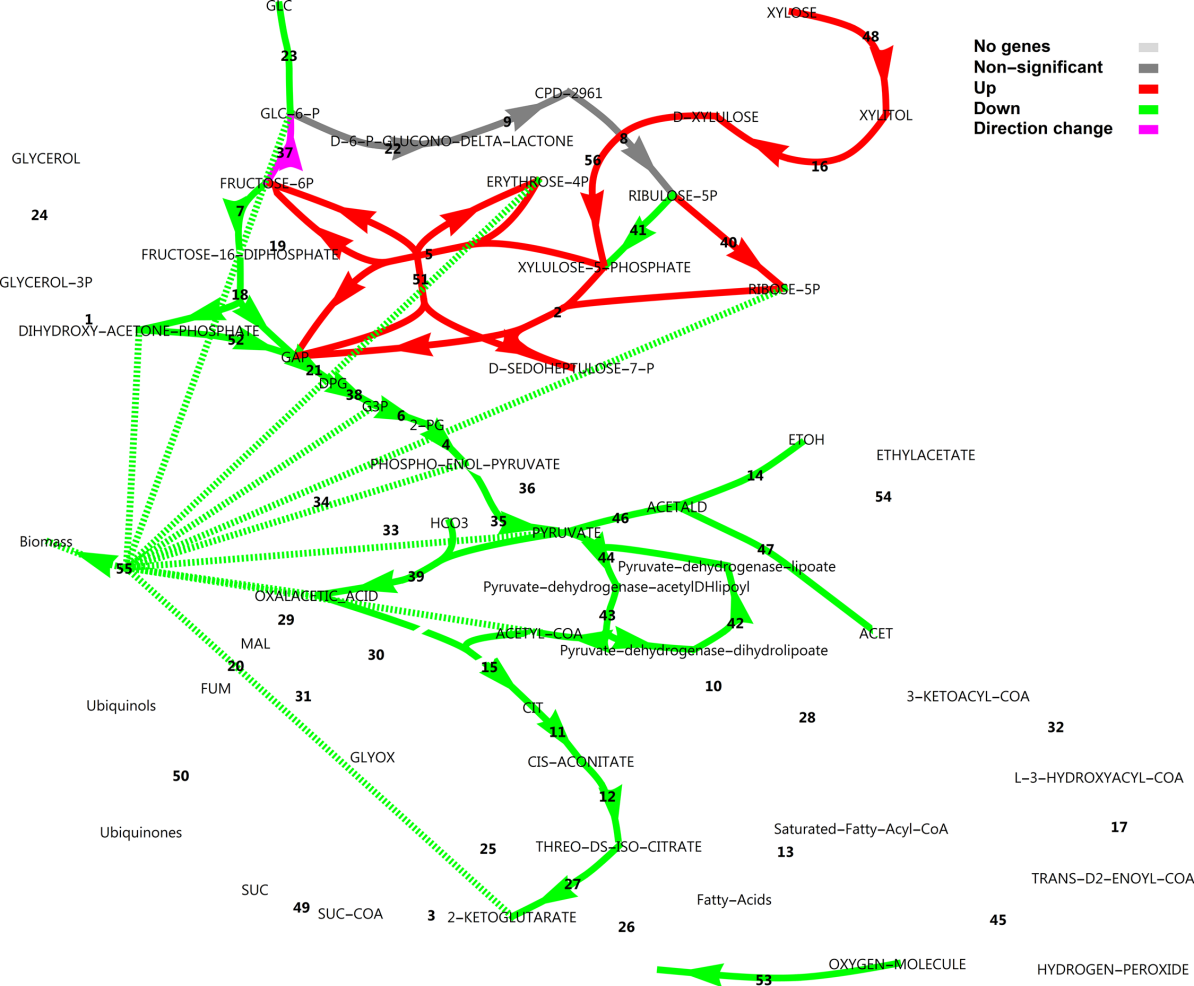
Differential flux analysis of central carbon metabolism. Fluxes which differed by less than 10% between conditions were considered constitutive. For reaction names, see S3 Pathway.

Differential metabolic flux analysis can be combined with differential gene expression data in the framework of MRA. From Figs 10 and 11 it is evident that in anaplerosis, phosphoenolpyruvate carboxykinase had very low transcript levels, while phosphoenolpyruvate carboxylase was absent from the annotation. Only the pyruvate carboxylase showed substantial transcript levels and was constitutively expressed. Hence, only pyruvate carboxylase was used in the model for flux estimations. In glucose medium, respiro-fermentative metabolism was observed. The specific uptake rate of glucose (on a dry cell weight basis) was 9.4 mmol h^-1^ mg^-1^ with acetate and ethanol produced at 2.0 and 2.7 mmol h^-1^ mg^-1^, respectively with no glycerol formation. In xylose medium, acetate, ethanol and glycerol were absent and the specific xylose uptake rate was estimated at approximately 5 mmol h^-1^ mg^-1^. The ethanol flux calculated could be considered as a conservative estimate, since some evaporation of ethanol could be expected from the aerobic condition. Fig 16 shows the differential flux outputs, as approximated by combining FBA with the consumption rates of sugars and production rates of ethanol and acetate (see S5 Table and S3 Pathway for the FBA model, parameters and MRA outputs). The xylose utilisation pathways as well as the transaldolases and transketolase fluxes of the non-oxidative PPP were up-regulated, while the direction of the glucose-6-phosphate isomerase was changed towards the direction of glucose-6-phosphate. Notably, the difference in oxidative pentose phosphate pathway flux was only 5% between glucose and xylose utilisation modes and was thus considered constitutive, as was found in the transcript levels. The rest of central metabolic fluxes were down-regulated. (See S3 Pathway for a more detailed log_2_(fold change) scheme). Although transcript levels do not generally correspond well to protein levels and hence to fluxes according to reports using model organisms [38], the overall patterns of expression in central carbon metabolism in our RNA-seq analysis corresponded well with the predicted flux patterns.

**Fig 17:**
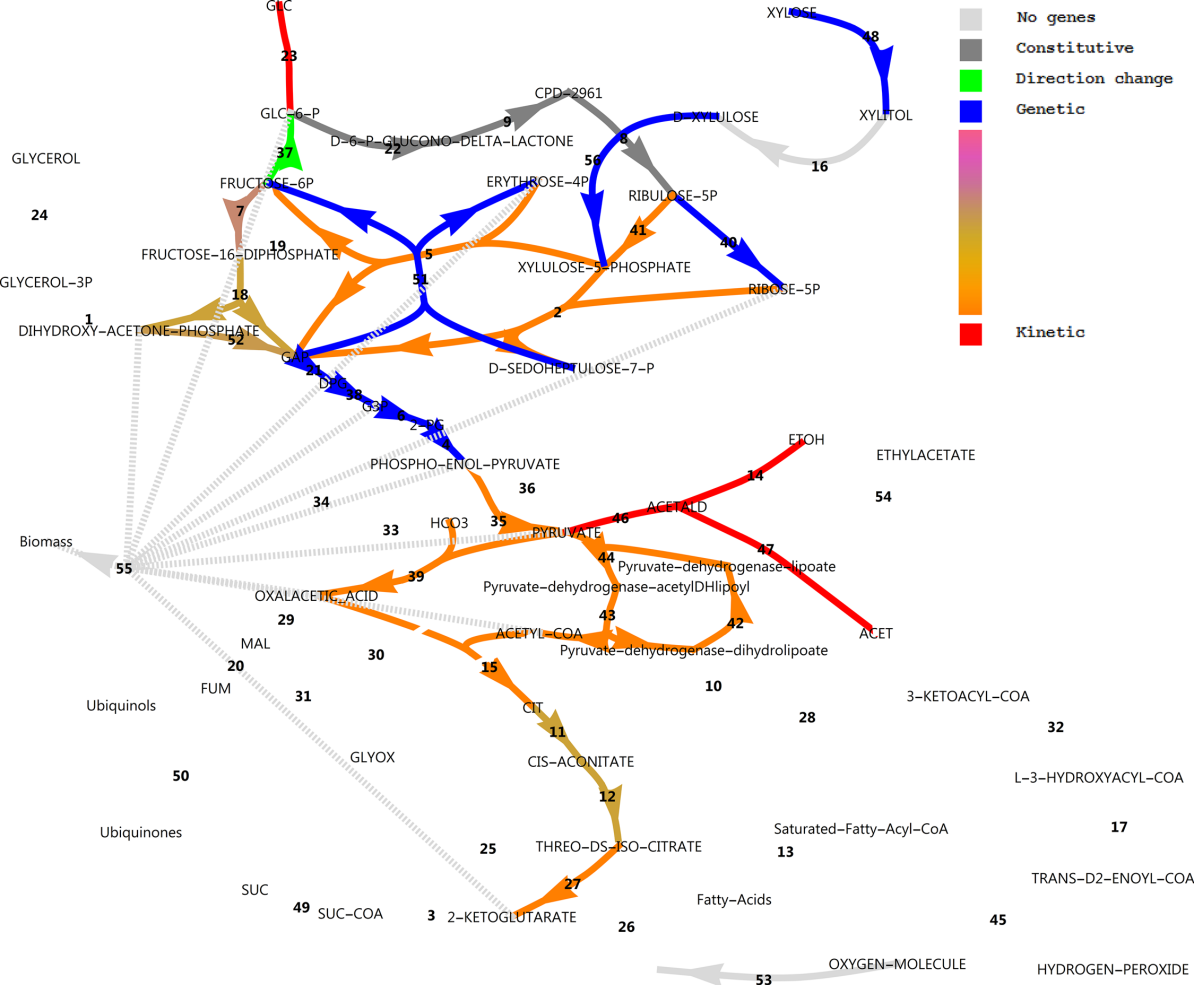
Metabolic Regulation Analysis of central carbon metabolism. For reaction names, see S3 Pathway.

Although there is not always a linear correlation between transcript level, protein concentrations and maximal activities of an enzyme, we assume differential transcript expression to provide a sufficient approximation to hierarchical regulation, as was done by others [39]. Fig 17 shows the separation of metabolic regulation into hierarchical and metabolic level regulation. It is evident that the genetic level up-regulation of xylose reductase, sorbitol dehydrogenase, xylulose kinase, transaldolase and ribose-5-phosphate isomerase rendered the regulation as purely hierarchical. Of these, sorbitol dehydrogenase acting as a xylitol dehydrogenase would be the most extreme example, as it showed a 208-fold genetic up-regulation. These four reactions interact with neighbouring reactions via the metabolites xylulose-5-phosphate, erythrose-4-phosphate, fructose-6-phosphate and ribose-5-phosphate to stimulate fluxes through the transketolases, and to reverse the flux through glucose-6-phosphate isomerase, whereas ribose-5-phsophate isomerase may lower the flux through ribulose-phosphate 3-epimerase by lowering the concentration of ribulose-5-phosphate. Notably, regulation of fluxes in lower glycolysis is dominated by the genetic component, whereas upper glycolysis is regulated approximately equally by the metabolic and genetic regulation levels. Downward from pyruvate kinase, regulation is dominated by changes in metabolite levels, while some contribution of the genetic regulation level is evident for the fluxes through the aconitate hydratases in the TCA cycle. Due to the absence of measured ethanol and acetate in the xylose medium, the disappearance of fluxes through pyruvate decarboxylase, alcohol dehydrogenase and aldehyde dehydrogenase rendered classification of regulation in these reactions as purely metabolic.

## Discussion

Modelling of bioprocesses has been attempted for a long time, in many different forms. It was realised in the second half of the twentieth century that several confounding factors had to be eliminated in the study of metabolism and its gene regulation, arguably with the growth rate as the most important confounding factor, since the expression level of many genes are correlated with the growth rate. Chemostat cultivation is a useful tool, since the growth rate can be easily controlled. However, for the vast majority of industrial applications, chemostats are unrealistic as industrial applications typically involve batch cultivation, or fed-batch cultivation to avoid catabolite repression, improve volumetric productivity and allow sufficient aeration by controlling the growth rate. Moreover, systems biology has become increasingly data-driven with the availability of high-throughput methods to measure large numbers of intracellular metabolites (metabolomics), proteins (proteomics) and especially RNAs (RNA-seq and microarray) from a single experiment. It is proposed here that much more could be learned in terms of genetic regulation in microorganisms by testing a variety of different cultivation conditions, especially substrates, in small batch experiments as compared to the same amount of work in a more sophisticated, labour intensive chemostat setup, or at least, complementary information. We showed that in a simple and cost-effective batch setup, a large amount of rich RNA-seq data could be generated and fruitfully explored. This small working volume also makes expensive isotope labelling studies feasible. In batch experiments, however, special care needs to be taken in terms of the timing of sampling, as, for instance, fermentation products would accumulate over time and a transcriptome sample late in the fermentation may be more reflective of chemical stress than of the metabolic mode. This may especially be a confounding factor when comparing the effects of the concentration of substrate. Our experimental design alleviated this effect and allowed us to focus on the effects of alternative substrates only.

Previously, exploration of omics datasets mostly focused on only the lists of the most significantly up-regulated or down-regulated genes separately, or on one or two more advanced types of analyses such as GO enrichment, and occasionally on the extraction of active networks. Here we presented one of the first examples that explore high-quality RNA-seq data from various perspectives, using different types of enrichments and networks, and rationalisation of the response with FBA and MRA as theoretical frameworks. Furthermore, combining information from databases such as GO and MetaCyc in the same problem and with subcellular compartmentalisation revealed several important features that would have been missed using either on its own.

The vastness of the xylose response under aerobic conditions indicates a different, opportunistic lifestyle that this yeast apparently adapts to when cultivated on xylose, which may be reminiscent of its natural environment where a variety of plant-derived substrates may be utilised. Down-regulation of many biosynthetic pathways is concordant with a lower growth rate, although there is a notable absence of growth rate specific gene sets in the enrichment statistics. Although many mitochondrial genes were differentially expressed, these are not the major enzymes in energy production in mitochondria. Oxidative stress also seems minimal in the aerobic xylose medium. Instead, a strong response is seen in the up-regulation of alternative sugar utilisation machinery, including inulinase, sugar transporters and catabolic routes for alternative sugars. Inulin is a fructan stored in large amounts in some plants, including *A*. *americana* [40]. Our strain was indeed isolated from an *A*. *americana* sample.

*K*. *marxianus* lacks enzymes such as secreted proteases, lipases and carbohydrate hydrolases. This yeast may thus be dependent on other fungi in the environment for these functions, or its natural habitat may be some commensal niche where these monomers are supplied by the plant. The transcriptional regulatory basis for this response is likely to be glucose repression by transcription factors such as *MIG1* binding to carbon source response elements, as was suggested to be the case for the inulinase gene *KmINU1* [4, 41].

It is interesting to find that the majority of genes for a complete organelle like the peroxisome, which is dedicated to lipid oxidation, was dramatically up-regulated in the xylose medium, yet without apparent function in the experimental setting. Thus, glucose de-repression is likely sufficient to activate most of the response to enable lipid catabolism, and stimulation by lipids may play a smaller role. The mitochondrial 2-methylcitrate pathway for the degradation of three-carbon molecules was also strongly up-regulated, suggesting that a variety of odd-chain fatty acids originating from peroxisomes may be oxidised.

FBA simulations predicted no significant up-regulation of the oxidative PPP flux, but a more intense up-regulation of the non-oxidative PPP flux and a down-regulation of glycolysis, which were consistent with RNA-seq data. Since biomass formation, the major sink for NADPH, is down-regulated in xylose medium, and another NADPH sink, xylose reductase, is up-regulated, it thus makes sense that the major source flux of NADPH (oxidative PPP) may be similar in both conditions (considering absolute fluxes normalised by the biomass concentration). Normalising fluxes to the uptake rate of a carbon source or to the biomass formation rate could thus be misleading when interpreting expression data. FBA simulations here thus shed light on what could be expected in terms of gene regulation.

MRA was used to separate metabolic regulation into hierarchical and metabolic levels. Reactions in lower glycolysis are known to a have high flux capacity due to high enzyme concentrations, and our transcript data also indicated this feature on glucose. Chemostat studies of *S*. *cerevisiae* classified these high-capacity reactions as well as the non-oxidative PPP reactions as pseudo-equilibrium or near-equilibrium reactions, suggesting that they can be sufficiently described by simple equations making use of thermodynamics and empirical studies at various dilution rates [42]; hence both detailed enzyme kinetic expressions and genetic regulation could be ignored. Reversible high capacity reactions like these should have low metabolic control coefficients over the flux and are not likely to be regulated at the genetic level—at least over small changes in the flux, close to a reference steady state. However, our transcript level data for *K*. *marxianus* showed that the transcript levels in lower glycolysis were substantially lower on xylose, and MRA showed a dominating hierarchical (genetic) level regulation. Flux through transaldolase was also dominated by hierarchical regulation. The contribution of hierarchical and metabolic level regulation of a reaction would differ between substrates utilised, and may also differ between species.

Reporter metabolite networking suggested that NAD(H), acetaldehyde, ethanol, glyceraldehyde-3-phosphate, 3-phosphoglycerate and hydrogen peroxide formed a strongly interconnected redox active system, with aldehyde dehydrogenases and alcohol dehydrogenases being the main players. This system may work across membranes, since acetaldehyde, ethanol and hydrogen peroxide can cross membranes, shuttles connect NAD(H) across compartments, and glyceraldehyde-3-phosphate and 3-phosphoglycerate are closely connected to NADH. At this stage it is not possible to resolve the fluxes and MRA through the acetaldehyde-dependent pyruvate dehydrogenase bypass. It was, however, evident that dramatic changes occurred in the transcript levels of the alcohol dehydrogenases and aldehyde dehydrogenases, including compartment-specific isozyme switching. As also suggested by Lertwattanasakul et al. [3], ethanol may be catabolised in the mitochondria as fast as it is produced. However, it has to be emphasised that care needs to be taken in extrapolating gene expression data between studies carried out under aerobic and anaerobic conditions. Oxygen limitation results in differential expression of many genes in yeasts.

## Conclusions

We believe to have captured in a unique manner, and from a number of perspectives, a complex transcriptional pattern telling an interesting story about how the cell ‘explores its options’ when the nutrient availability changes under aerobic conditions. Strong up-regulation of transporters and pathways for utilisation of alternative carbohydrates was evident. In addition, the more opportunistic lifestyle was supported by invasive growth, and sexual reproduction was activated as a long-term survival strategy. The strong peroxisomal fatty acid catabolic response accompanied by the mitochondrial 2-methylcitrate pathway is likely explained by glucose de-repression, similar to that seen for carbohydrate utilisation. As *K*. *marxianus* seemingly lacks the secreted enzymes required for depolymerisation of biopolymers, the species is probably dependent on other species for supply of monomers such as sugars, amino acids and free fatty acids, whereas inulinase is a specialist feature enabling this species to utilise this plant storage oligosaccharide. It would be interesting to see whether xylose may have a stimulatory role as suggested recently for *Saccharomyces cerevisiae* [43] and through which signaling pathways this may take place.

MRA was demonstrated here as an informative method to dissect the regulation of fluxes into the metabolic and hierarchical levels. It is evident that the genetic level plays a dominating role in the regulation of fluxes in central carbon metabolism, not only in the early enabling steps of utilisation of xylose as the carbon source, but also in the high capacity reactions of lower glycolysis. In kinetic modelling of metabolism, emphasis should thus be placed on genetic regulation, which is currently very challenging. Multiple omics would need to be combined to predict such regulatory networks and build realistic models. However, in order to resolve fluxes originating from pyruvate, isotopic tracer studies would be required, which currently is under investigation. In addition, the isozyme switching observed with alcohol dehydrogenase, aldehyde dehydrogenase and acetate-CoA ligase calls for a detailed investigation into the compartmentalisation and kinetics of these enzymes, and detailed bottom-up kinetic modelling to understand the role of this interesting behaviour.

## Supporting Information

S1 Draft Genome Draft genome for *K*. *marxianus* UFS-Y2791 reconstructed *de novo*. (FA)

S1 Network Pathway-to-pathway Network. (PDF)

S2 Network Molecule-to-molecule network. (CDF)

S3 Network Enzyme-Reporter metabolite network. (CDF)

S4 Network Enzyme-reporter metabolite network (NAD). (CDF)

S5 Network Enzyme-reporter metabolite network (NADP). (CDF)

S1 ORF Putative open reading frames found in the genome. (GFF)

S1 Pathway RNA-seq data mapping to central metabolism. (PPTX)

S2 Pathway Pathway multi-map. (PDF)

S3 Pathway Estimated metabolic flux maps. (PPTX)

S1 Proteins Amino acid sequences. (AA)

S1 Table Main data tables. (XLSX)

S2 Table Enrichment statistics of GO cellular_component. (XLSX)

S3 Table Enrichment statistics of GO molecular_function. (XLSX)

S4 Table Enrichment statistics of GO biochemical_process. (XLSX)

S5 Table Metabolic flux model and Metabolic Regulation Analysis. (XLSX)

S1 Text Supplementary text on gene set enrichment and extracellular enzymes. (PDF)
