# Supplementary material for: Differential RNA-seq, Multi-Network Analysis and Metabolic Regulation Analysis of Kluyveromyces marxianus Reveals a Compartmentalised Response to Xylose
Source: PLoS One. 2016 Jun 17;11(6):e0156242. doi: 10.1371/journal.pone.0156242 (PMC4912071; doi:10.1371/journal.pone.0156242)
Supplement: S1 Pathway — (PPTX) [file pone.0156242.s008.pptx]

## Slide 1
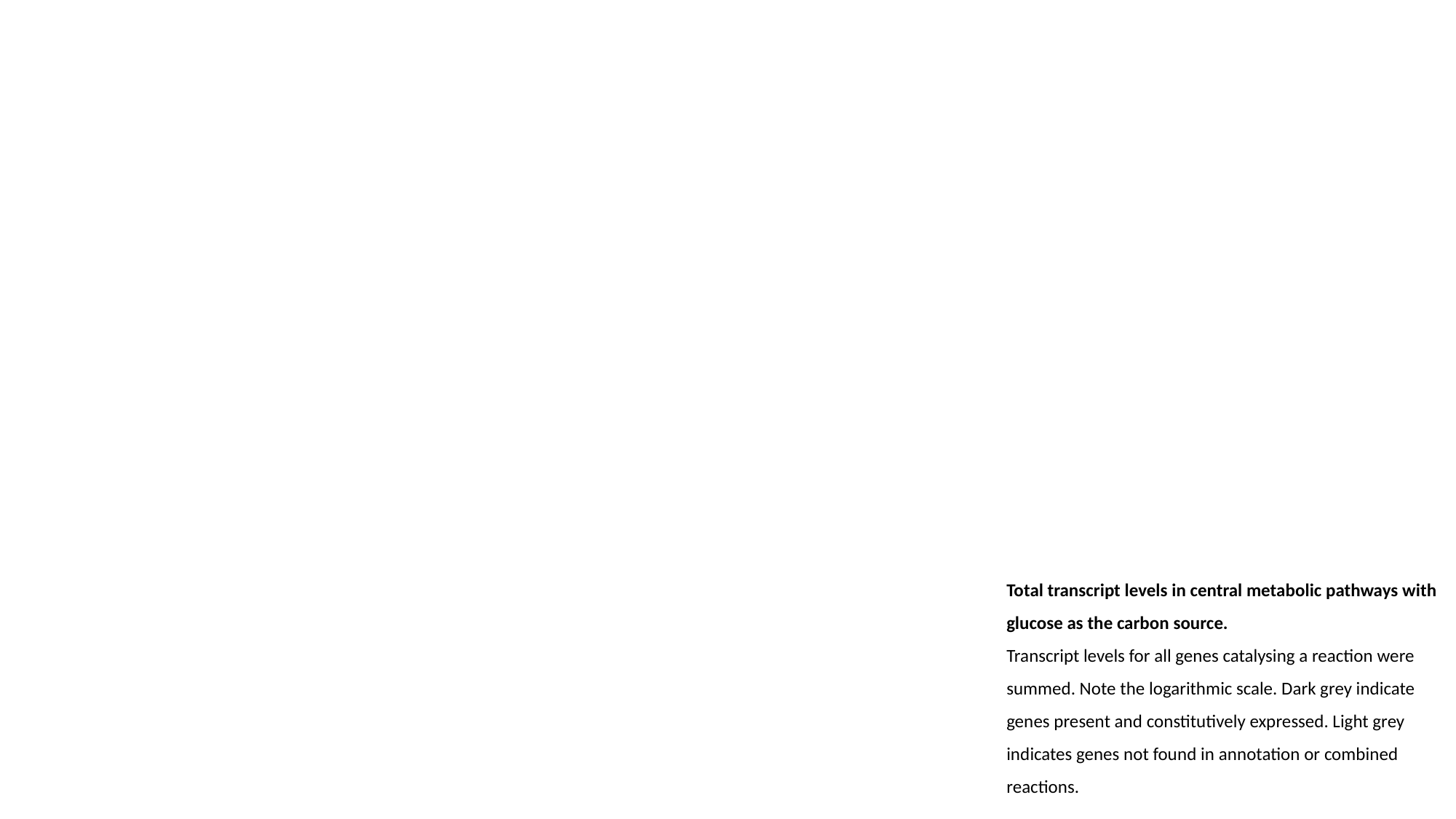

Total transcript levels in central metabolic pathways with glucose as the carbon source.
Transcript levels for all genes catalysing a reaction were summed. Note the logarithmic scale. Dark grey indicate genes present and constitutively expressed. Light grey indicates genes not found in annotation or combined reactions.

## Slide 2
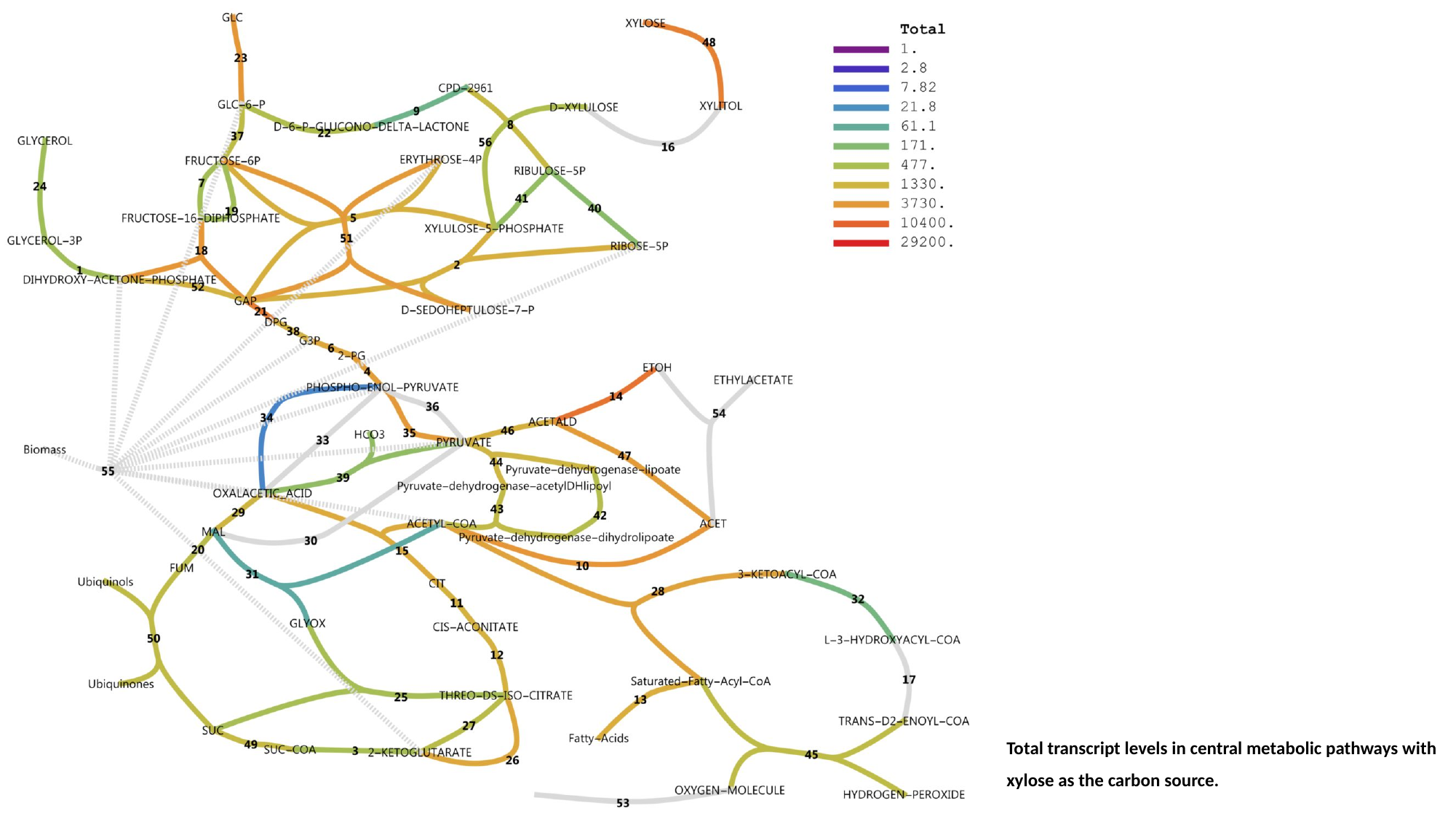

Total transcript levels in central metabolic pathways with xylose as the carbon source.

## Slide 3
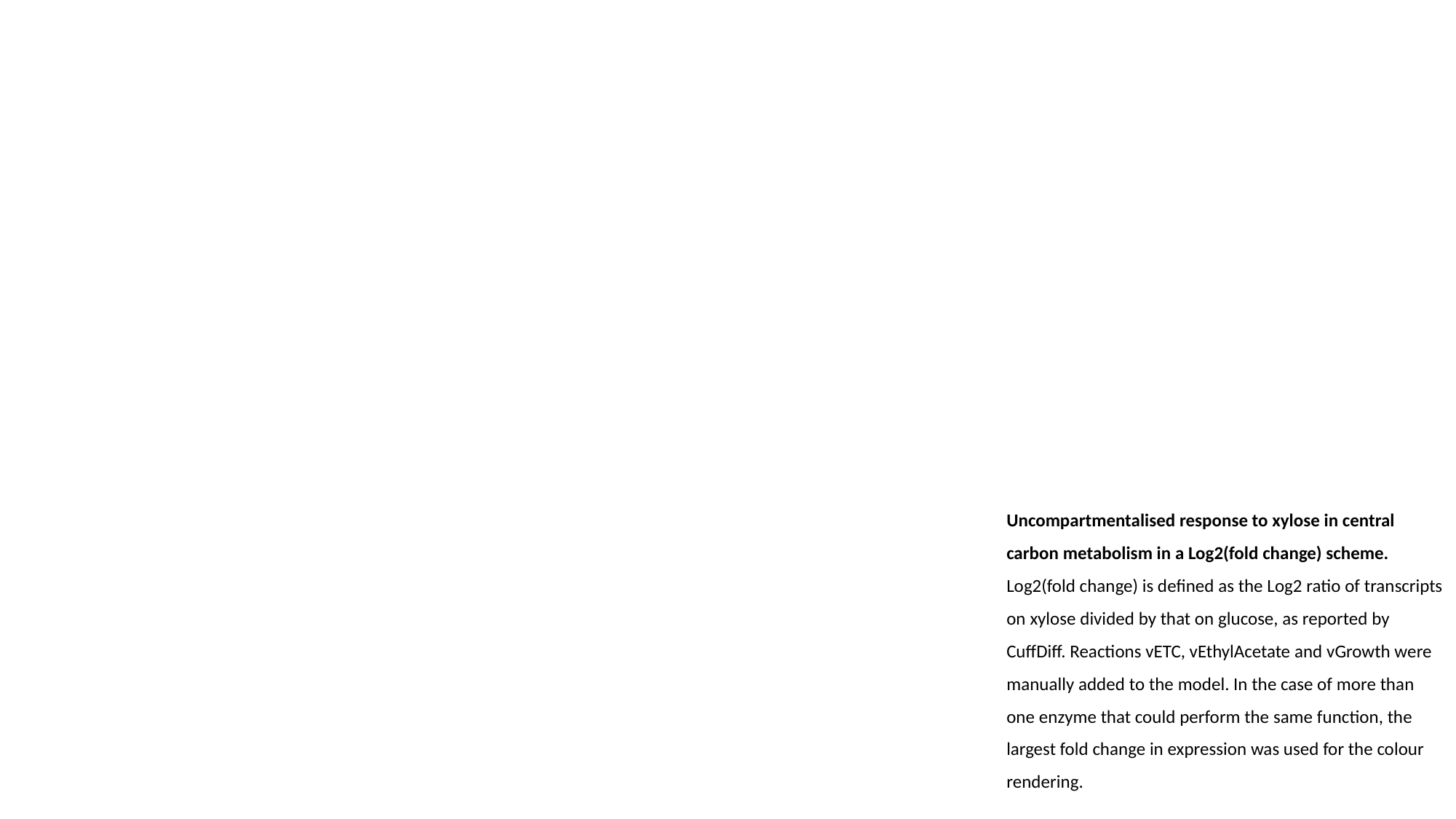

Uncompartmentalised response to xylose in central carbon metabolism in a Log2(fold change) scheme.
Log2(fold change) is defined as the Log2 ratio of transcripts on xylose divided by that on glucose, as reported by CuffDiff. Reactions vETC, vEthylAcetate and vGrowth were manually added to the model. In the case of more than one enzyme that could perform the same function, the largest fold change in expression was used for the colour rendering.

## Slide 4
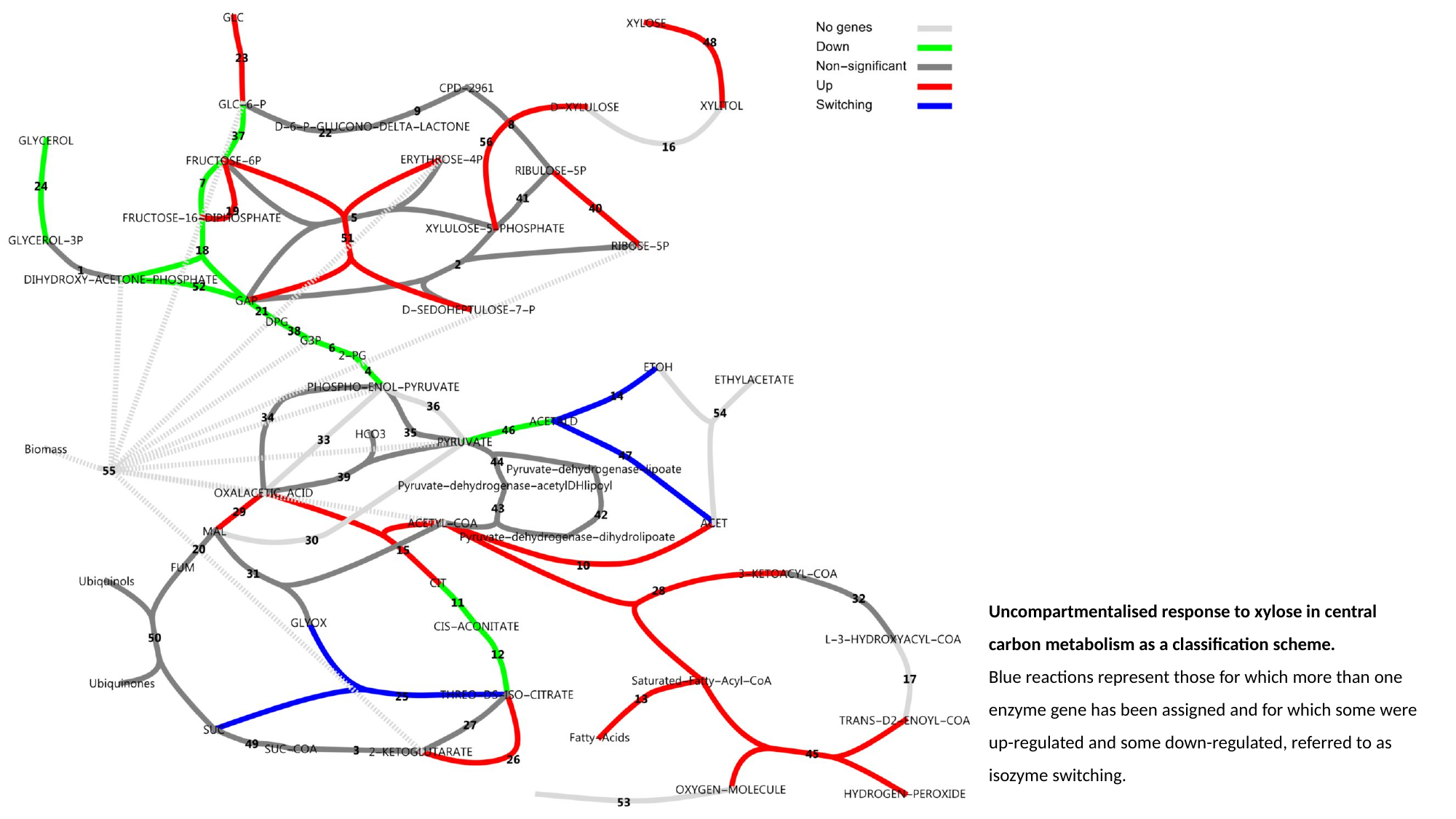

Uncompartmentalised response to xylose in central carbon metabolism as a classification scheme.
Blue reactions represent those for which more than one enzyme gene has been assigned and for which some were up-regulated and some down-regulated, referred to as isozyme switching.

## Slide 5
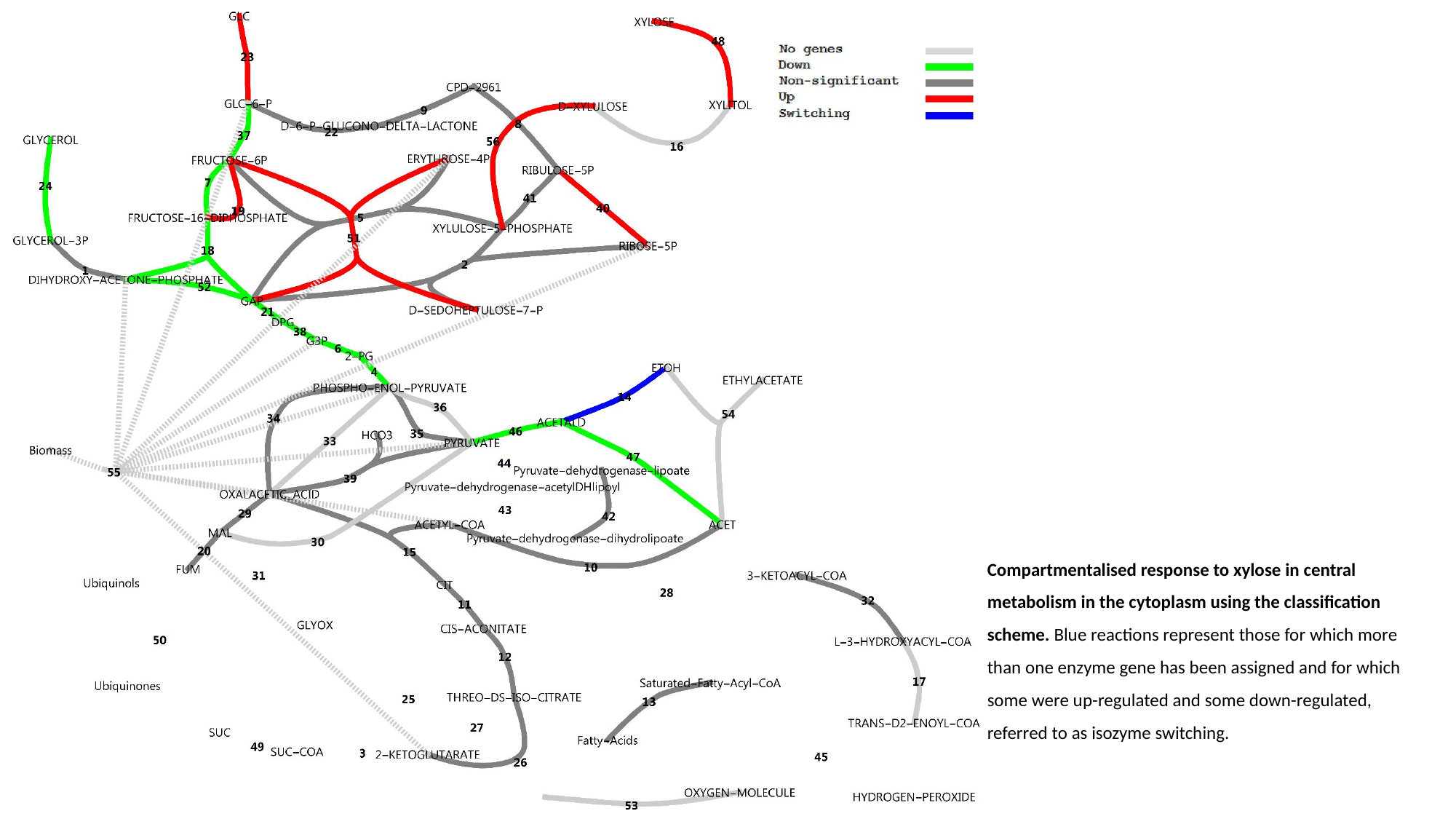

Compartmentalised response to xylose in central metabolism in the cytoplasm using the classification scheme. Blue reactions represent those for which more than one enzyme gene has been assigned and for which some were up-regulated and some down-regulated, referred to as isozyme switching.

## Slide 6
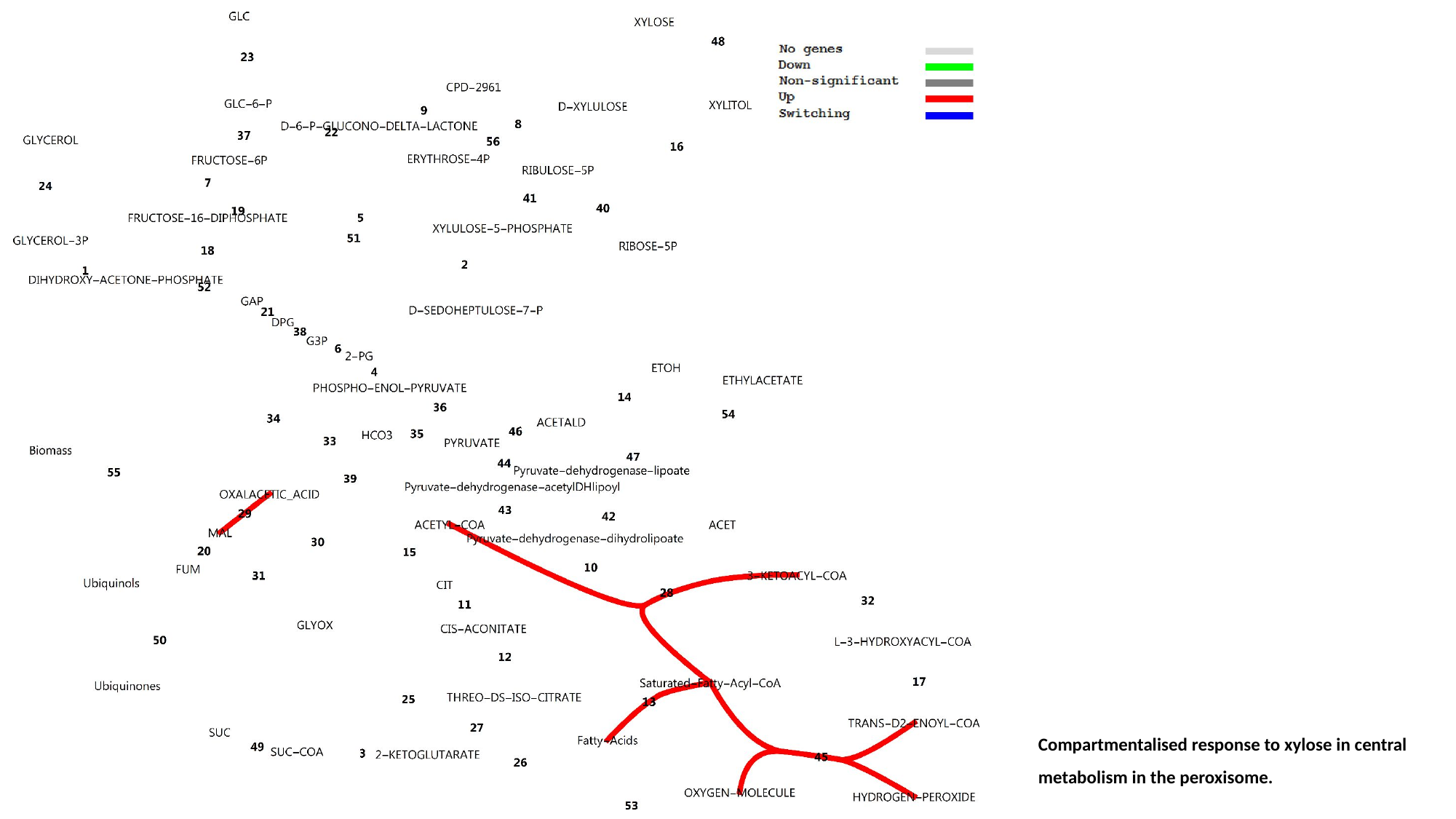

Compartmentalised response to xylose in central metabolism in the peroxisome.

## Slide 7
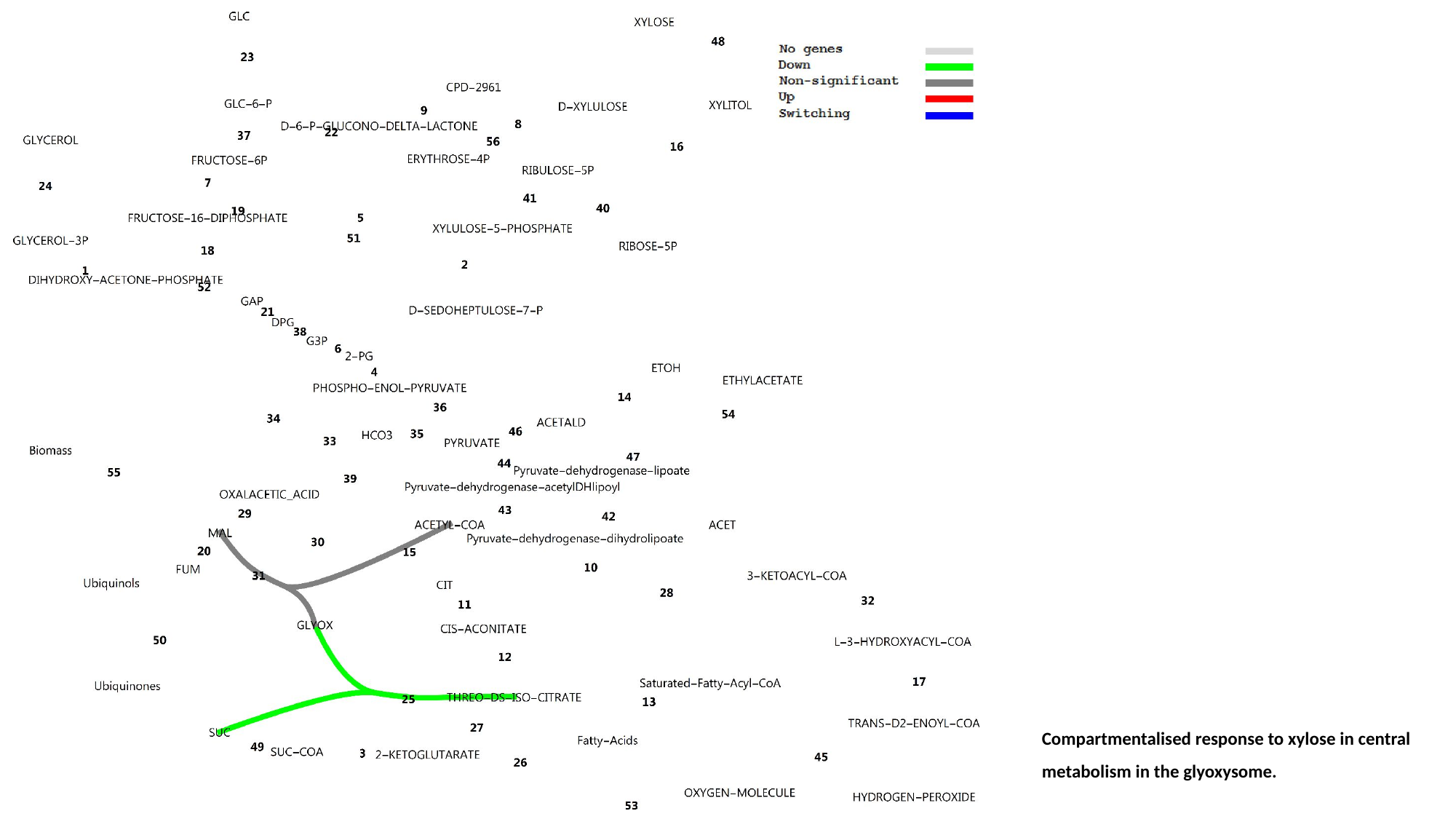

Compartmentalised response to xylose in central metabolism in the glyoxysome.

## Slide 8
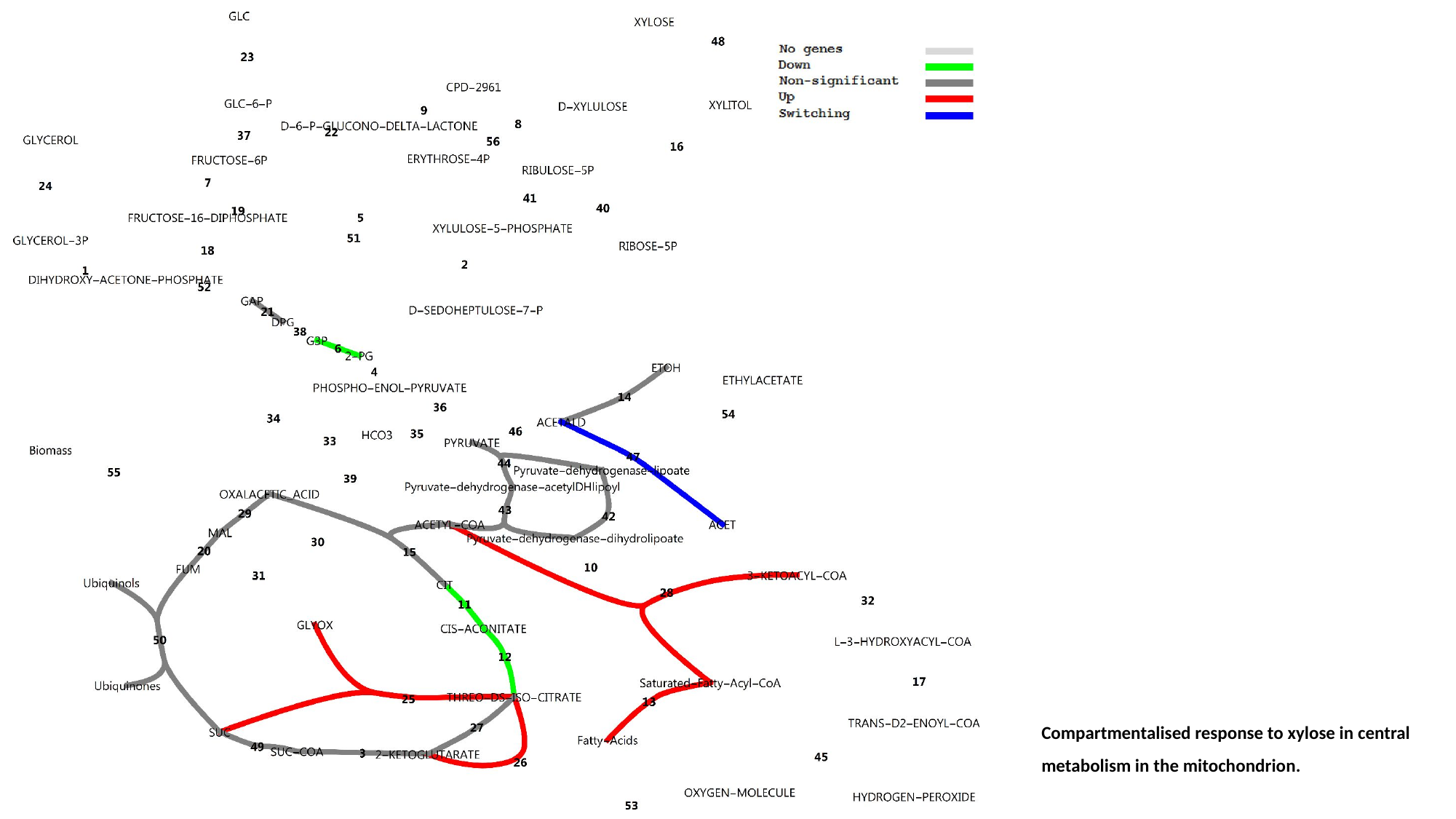

Compartmentalised response to xylose in central metabolism in the mitochondrion.

## Slide 9
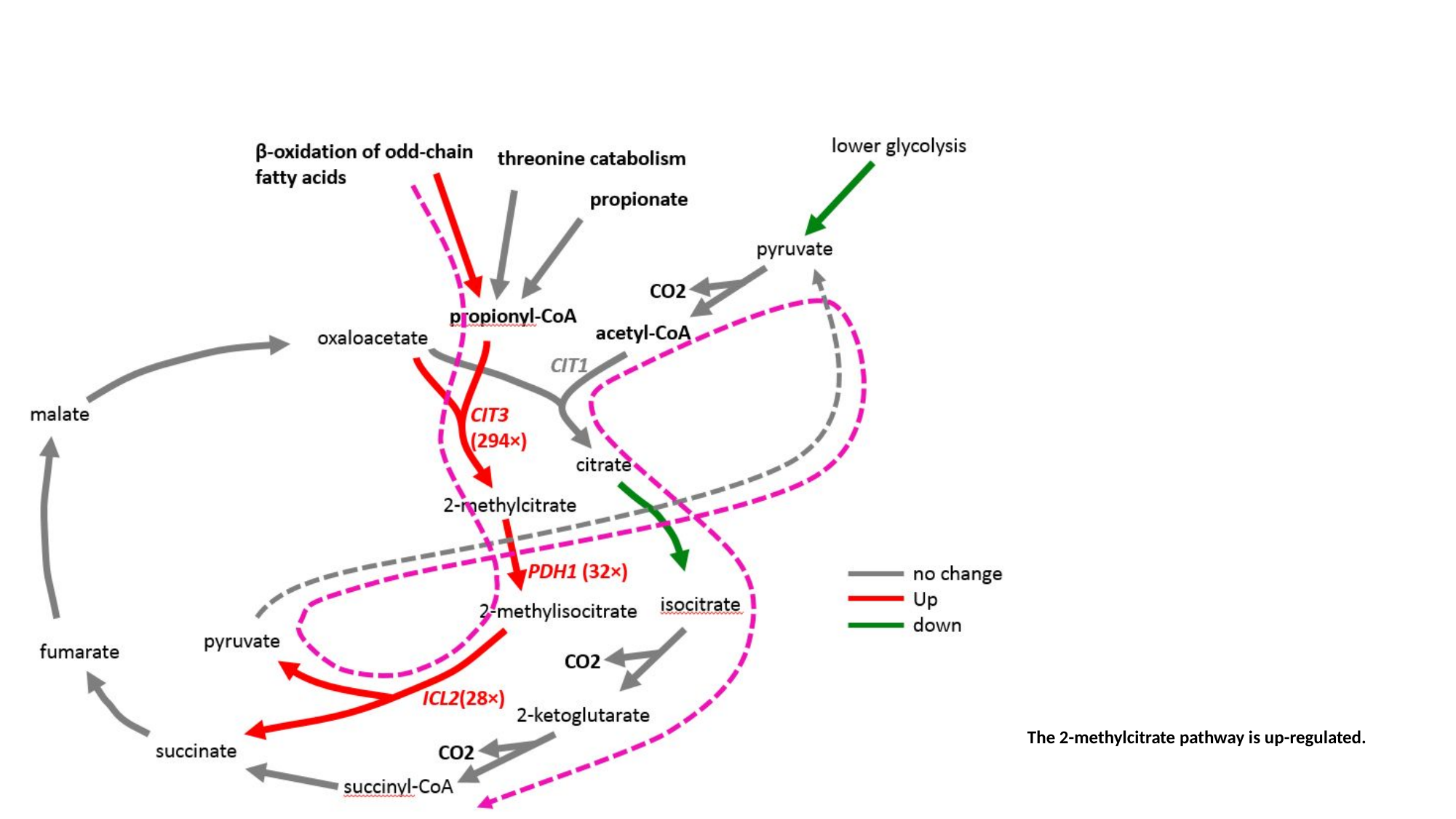

The 2-methylcitrate pathway is up-regulated.

## Slide 10
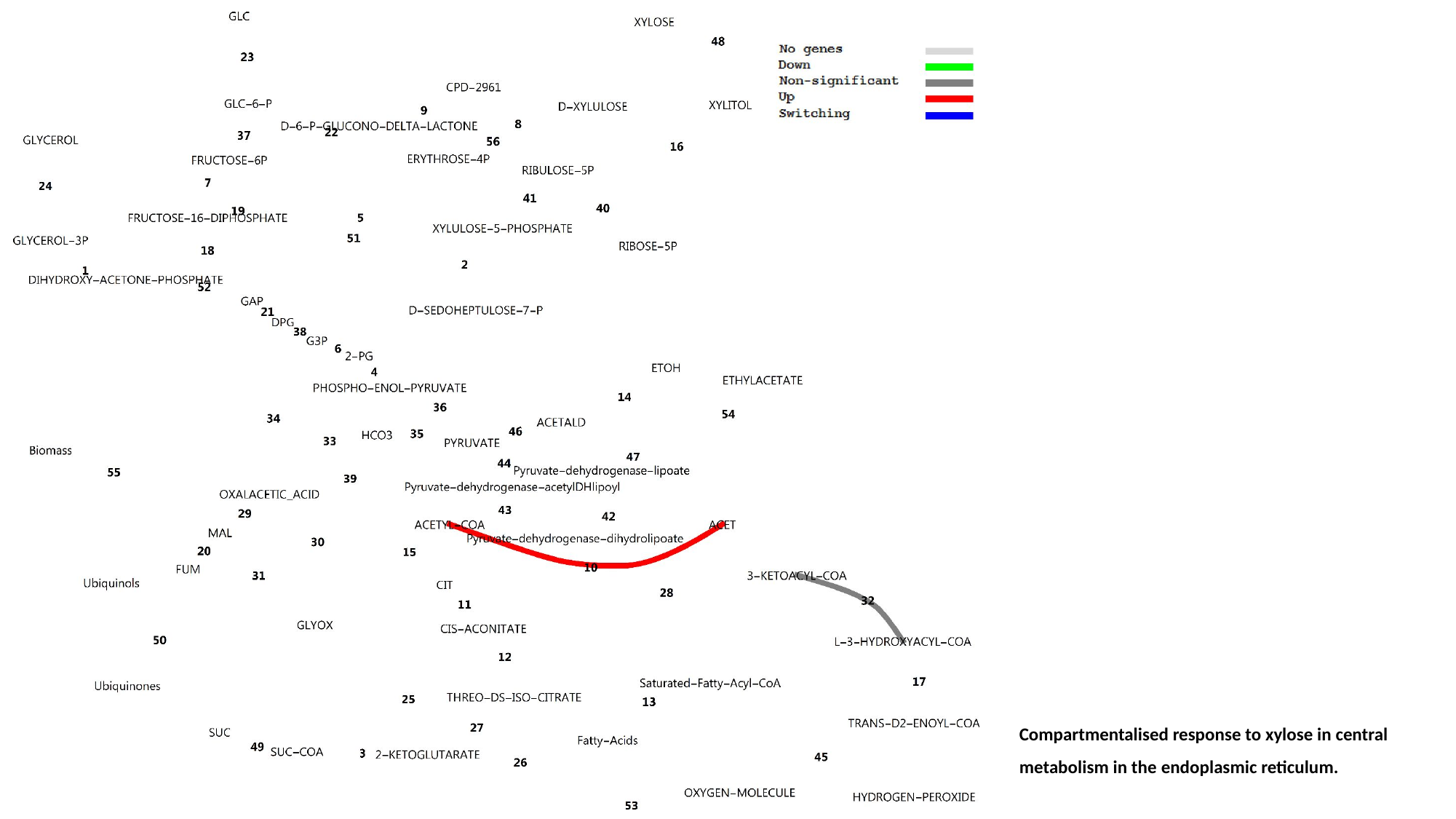

Compartmentalised response to xylose in central metabolism in the endoplasmic reticulum.

## Slide 11
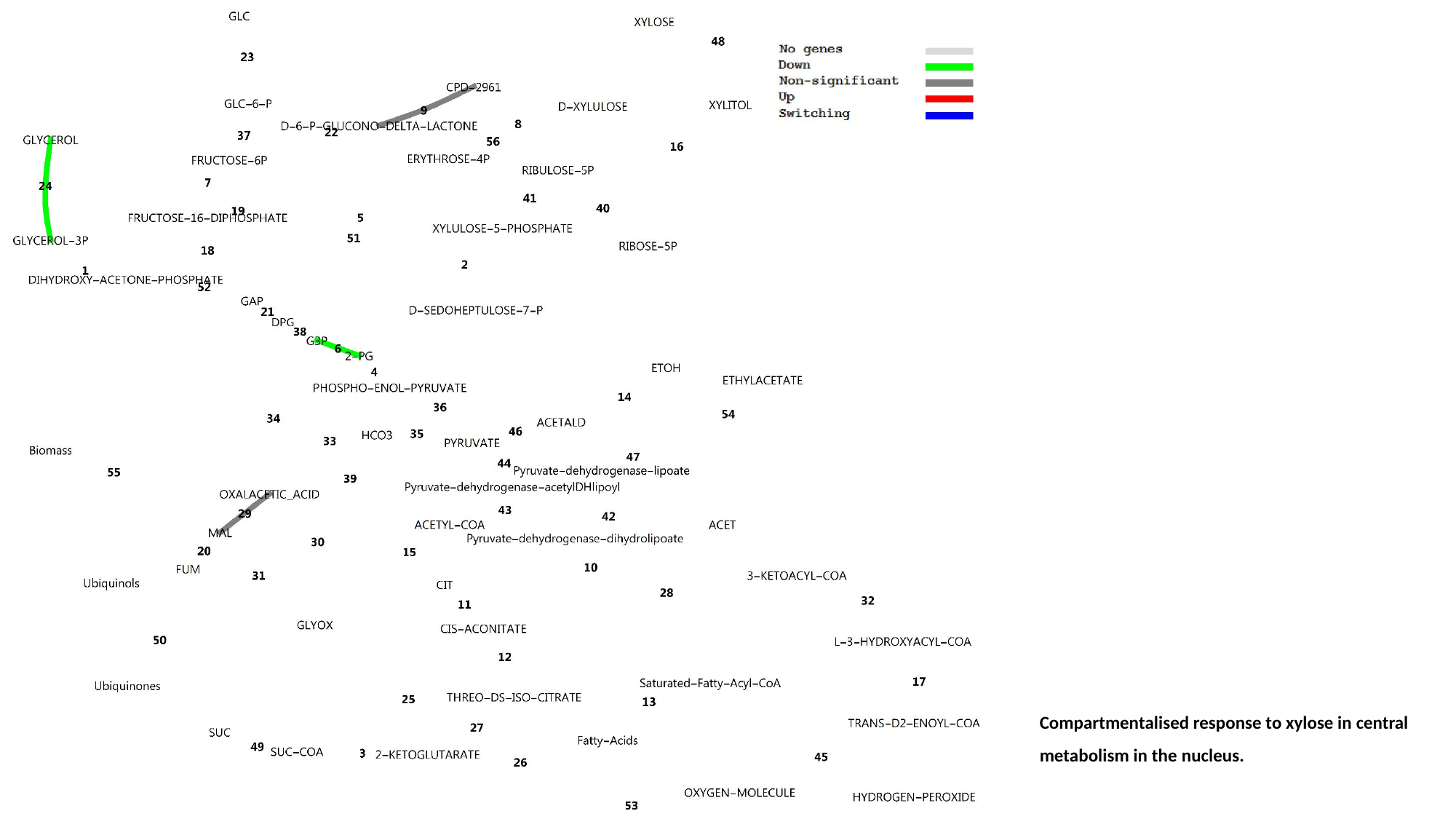

Compartmentalised response to xylose in central metabolism in the nucleus.

## Slide 12
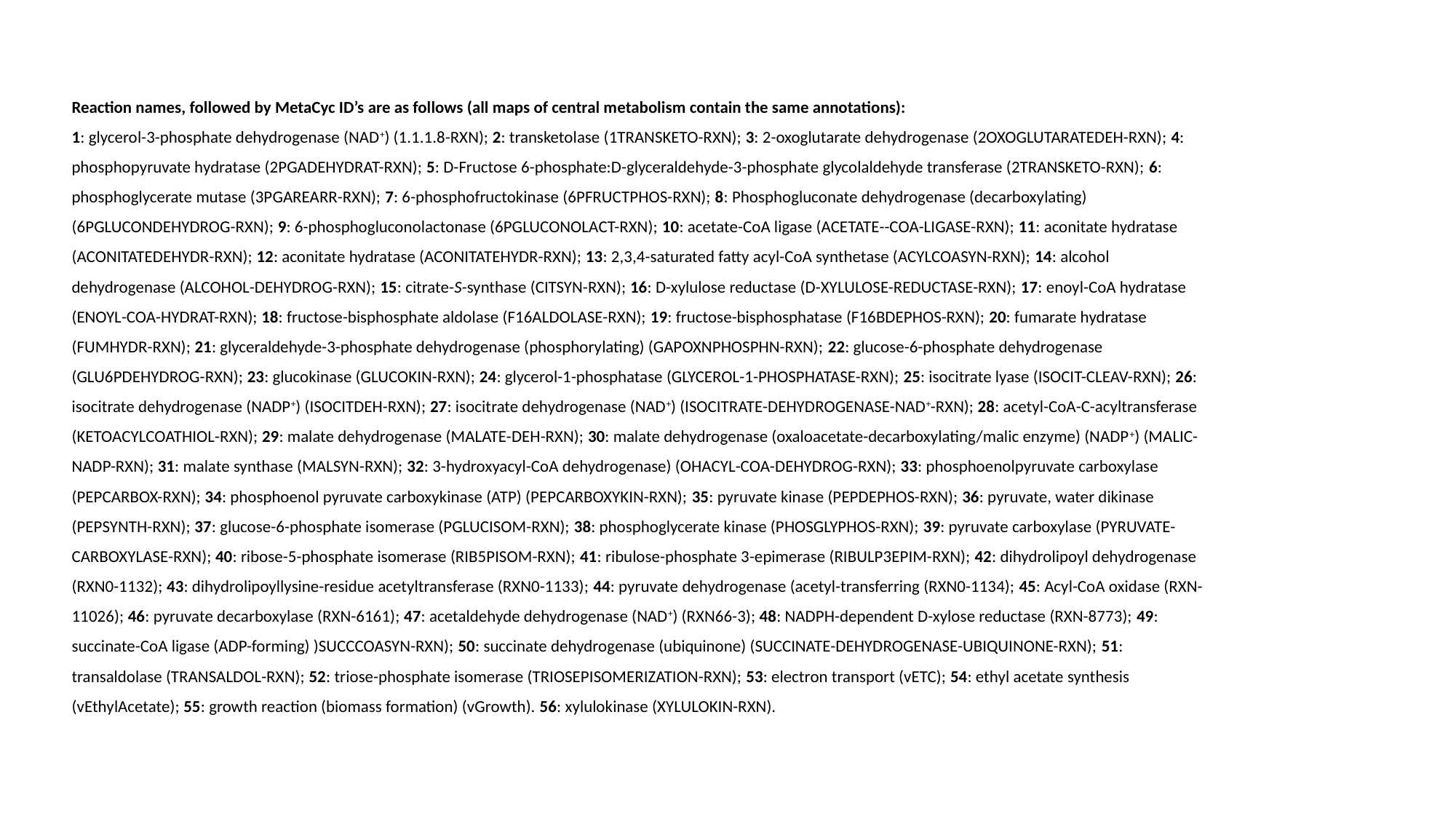

Reaction names, followed by MetaCyc ID’s are as follows (all maps of central metabolism contain the same annotations):
1: glycerol-3-phosphate dehydrogenase (NAD+) (1.1.1.8-RXN); 2: transketolase (1TRANSKETO-RXN); 3: 2-oxoglutarate dehydrogenase (2OXOGLUTARATEDEH-RXN); 4: phosphopyruvate hydratase (2PGADEHYDRAT-RXN); 5: D-Fructose 6-phosphate:D-glyceraldehyde-3-phosphate glycolaldehyde transferase (2TRANSKETO-RXN); 6: phosphoglycerate mutase (3PGAREARR-RXN); 7: 6-phosphofructokinase (6PFRUCTPHOS-RXN); 8: Phosphogluconate dehydrogenase (decarboxylating) (6PGLUCONDEHYDROG-RXN); 9: 6-phosphogluconolactonase (6PGLUCONOLACT-RXN); 10: acetate-CoA ligase (ACETATE--COA-LIGASE-RXN); 11: aconitate hydratase (ACONITATEDEHYDR-RXN); 12: aconitate hydratase (ACONITATEHYDR-RXN); 13: 2,3,4-saturated fatty acyl-CoA synthetase (ACYLCOASYN-RXN); 14: alcohol dehydrogenase (ALCOHOL-DEHYDROG-RXN); 15: citrate-S-synthase (CITSYN-RXN); 16: D-xylulose reductase (D-XYLULOSE-REDUCTASE-RXN); 17: enoyl-CoA hydratase (ENOYL-COA-HYDRAT-RXN); 18: fructose-bisphosphate aldolase (F16ALDOLASE-RXN); 19: fructose-bisphosphatase (F16BDEPHOS-RXN); 20: fumarate hydratase (FUMHYDR-RXN); 21: glyceraldehyde-3-phosphate dehydrogenase (phosphorylating) (GAPOXNPHOSPHN-RXN); 22: glucose-6-phosphate dehydrogenase (GLU6PDEHYDROG-RXN); 23: glucokinase (GLUCOKIN-RXN); 24: glycerol-1-phosphatase (GLYCEROL-1-PHOSPHATASE-RXN); 25: isocitrate lyase (ISOCIT-CLEAV-RXN); 26: isocitrate dehydrogenase (NADP+) (ISOCITDEH-RXN); 27: isocitrate dehydrogenase (NAD+) (ISOCITRATE-DEHYDROGENASE-NAD+-RXN); 28: acetyl-CoA-C-acyltransferase (KETOACYLCOATHIOL-RXN); 29: malate dehydrogenase (MALATE-DEH-RXN); 30: malate dehydrogenase (oxaloacetate-decarboxylating/malic enzyme) (NADP+) (MALIC-NADP-RXN); 31: malate synthase (MALSYN-RXN); 32: 3-hydroxyacyl-CoA dehydrogenase) (OHACYL-COA-DEHYDROG-RXN); 33: phosphoenolpyruvate carboxylase (PEPCARBOX-RXN); 34: phosphoenol pyruvate carboxykinase (ATP) (PEPCARBOXYKIN-RXN); 35: pyruvate kinase (PEPDEPHOS-RXN); 36: pyruvate, water dikinase (PEPSYNTH-RXN); 37: glucose-6-phosphate isomerase (PGLUCISOM-RXN); 38: phosphoglycerate kinase (PHOSGLYPHOS-RXN); 39: pyruvate carboxylase (PYRUVATE-CARBOXYLASE-RXN); 40: ribose-5-phosphate isomerase (RIB5PISOM-RXN); 41: ribulose-phosphate 3-epimerase (RIBULP3EPIM-RXN); 42: dihydrolipoyl dehydrogenase (RXN0-1132); 43: dihydrolipoyllysine-residue acetyltransferase (RXN0-1133); 44: pyruvate dehydrogenase (acetyl-transferring (RXN0-1134); 45: Acyl-CoA oxidase (RXN-11026); 46: pyruvate decarboxylase (RXN-6161); 47: acetaldehyde dehydrogenase (NAD+) (RXN66-3); 48: NADPH-dependent D-xylose reductase (RXN-8773); 49: succinate-CoA ligase (ADP-forming) )SUCCCOASYN-RXN); 50: succinate dehydrogenase (ubiquinone) (SUCCINATE-DEHYDROGENASE-UBIQUINONE-RXN); 51: transaldolase (TRANSALDOL-RXN); 52: triose-phosphate isomerase (TRIOSEPISOMERIZATION-RXN); 53: electron transport (vETC); 54: ethyl acetate synthesis (vEthylAcetate); 55: growth reaction (biomass formation) (vGrowth). 56: xylulokinase (XYLULOKIN-RXN).
