# Supplementary material for: Differential RNA-seq, Multi-Network Analysis and Metabolic Regulation Analysis of Kluyveromyces marxianus Reveals a Compartmentalised Response to Xylose
Source: PLoS One. 2016 Jun 17;11(6):e0156242. doi: 10.1371/journal.pone.0156242 (PMC4912071; doi:10.1371/journal.pone.0156242)
Supplement: S2 Pathway — (PDF) [file pone.0156242.s009.pdf]

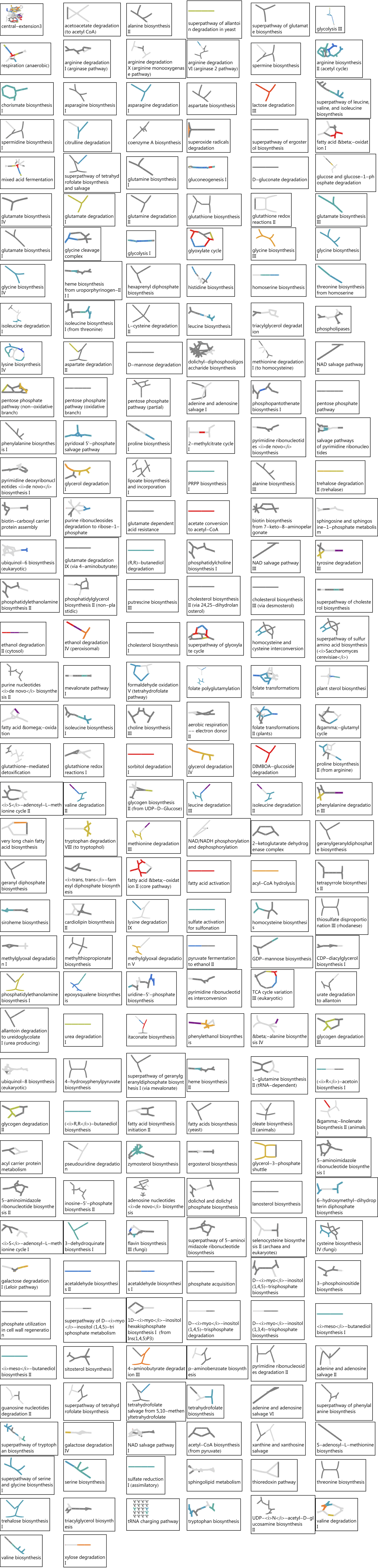
central−extension3
acetoacetate degradation (to acetyl CoA)
alanine biosynthesis II
superpathway of allantoin degradation in yeast
superpathway of glutamate biosynthesis
glycolysis III
respiration (anaerobic)
arginine degradation I (arginase pathway)
arginine degradation X (arginine monooxygenase pathway)
arginine degradation VI (arginase 2 pathway)
spermine biosynthesis
arginine biosynthesis II (acetyl cycle)
chorismate biosynthesis I
asparagine biosynthesis I
asparagine degradation I
aspartate biosynthesis
lactose degradation III
superpathway of leucine, valine, and isoleucine biosynthesis
spermidine biosynthesis I
citrulline degradation
coenzyme A biosynthesis
superoxide radicals degradation
superpathway of ergosterol biosynthesis
fatty acid β−oxidation I
mixed acid fermentation
superpathway of tetrahydrofolate biosynthesis and salvage
glutamine biosynthesis II
gluconeogenesis I
D−gluconate degradation
glucose and glucose−1−phosphate degradation
glutamate biosynthesis IV
glutamate degradation I
glutamine degradation II
glutathione biosynthesis
glutathione redox reactions II
glutamate biosynthesis III
glutamate biosynthesis I
glycine cleavage complex
glycolysis I
glyoxylate cycle
glycine biosynthesis III
glycine biosynthesis I
glycine biosynthesis IV
heme biosynthesis from uroporphyrinogen−III I
hexaprenyl diphosphate biosynthesis
histidine biosynthesis
homoserine biosynthesis
threonine biosynthesis from homoserine
isoleucine degradation I
isoleucine biosynthesis I (from threonine)
L−cysteine degradation II
leucine biosynthesis
triacylglycerol degradation
phospholipases
lysine biosynthesis IV
aspartate degradation II
D−mannose degradation
dolichyl−diphosphooligosaccharide biosynthesis
methionine degradation I (to homocysteine)
NAD salvage pathway II
pentose phosphate pathway (non−oxidative branch)
pentose phosphate pathway (oxidative branch)
pentose phosphate pathway (partial)
adenine and adenosine salvage I
phosphopantothenate biosynthesis I
pentose phosphate pathway
phenylalanine biosynthesis I
pyridoxal 5'−phosphate salvage pathway
proline biosynthesis I
2−methylcitrate cycle I
pyrimidine ribonucleotides <i>de novo</i> biosynthesis
salvage pathways of pyrimidine ribonucleotides
pyrimidine deoxyribonucleotides <i>de novo</i> biosynthesis I
glycerol degradation I
lipoate biosynthesis and incorporation I
PRPP biosynthesis I
alanine biosynthesis III
trehalose degradation II (trehalase)
biotin−carboxyl carrier protein assembly
purine ribonucleosides degradation to ribose−1−phosphate
glutamate dependent acid resistance
acetate conversion to acetyl−CoA
biotin biosynthesis from 7−keto−8−aminopelargonate
sphingosine and sphingosine−1−phosphate metabolism
ubiquinol−6 biosynthesis (eukaryotic)
glutamate degradation IX (via 4−aminobutyrate)
(R,R)−butanediol degradation
phosphatidylcholine biosynthesis I
NAD salvage pathway III
tyrosine degradation I
phosphatidylethanolamine biosynthesis II
phosphatidylglycerol biosynthesis II (non−plastidic)
putrescine biosynthesis III
cholesterol biosynthesis II (via 24,25−dihydrolanosterol)
cholesterol biosynthesis III (via desmosterol)
superpathway of cholesterol biosynthesis
ethanol degradation II (cytosol)
ethanol degradation IV (peroxisomal)
cholesterol biosynthesis I
superpathway of glyoxylate cycle
homocysteine and cysteine interconversion
superpathway of sulfur amino acid biosynthesis (<i>Saccharomyces cerevisiae</i>)
purine nucleotides <i>de novo</i> biosynthesis II
mevalonate pathway I
formaldehyde oxidation V (tetrahydrofolate pathway)
folate polyglutamylation
folate transformations I
plant sterol biosynthesis
fatty acid ω−oxidation
isoleucine biosynthesis I
choline biosynthesis III
aerobic respiration −− electron donor II
folate transformations II (plants)
γ−glutamyl cycle
glutathione−mediated detoxification
glutathione redox reactions I
sorbitol degradation I
glycerol degradation IV
DIMBOA−glucoside degradation
proline biosynthesis II (from arginine)
<i>S</i>−adenosyl−L−methionine cycle II
valine degradation I
glycogen biosynthesis II (from UDP−D−Glucose)
leucine degradation III
isoleucine degradation II
phenylalanine degradation III
very long chain fatty acid biosynthesis
tryptophan degradation VIII (to tryptophol)
methionine degradation III
NAD/NADH phosphorylation and dephosphorylation
2−ketoglutarate dehydrogenase complex
geranylgeranyldiphosphate biosynthesis
geranyl diphosphate biosynthesis
<i>trans, trans</i>−farnesyl diphosphate biosynthesis
fatty acid β−oxidation II (core pathway)
fatty acid activation
acyl−CoA hydrolysis
tetrapyrrole biosynthesis II
siroheme biosynthesis
cardiolipin biosynthesis II
lysine degradation IX
sulfate activation for sulfonation
homocysteine biosynthesis
thiosulfate disproportionation III (rhodanese)
methylglyoxal degradation I
methylthiopropionate biosynthesis
methylglyoxal degradation V
pyruvate fermentation to ethanol II
GDP−mannose biosynthesis
CDP−diacylglycerol biosynthesis I
phosphatidylethanolamine biosynthesis I
epoxysqualene biosynthesis
uridine−5'−phosphate biosynthesis
pyrimidine ribonucleotides interconversion
TCA cycle variation III (eukaryotic)
urate degradation to allantoin
allantoin degradation to ureidoglycolate I (urea producing)
urea degradation I
itaconate biosynthesis
phenylethanol biosynthesis
β−alanine biosynthesis IV
glycogen degradation III
ubiquinol−8 biosynthesis (eukaryotic)
4−hydroxyphenylpyruvate biosynthesis
superpathway of geranylgeranyldiphosphate biosynthesis I (via mevalonate)
heme biosynthesis II
L−glutamine biosynthesis II (tRNA−dependent)
(<i>R</i>)−acetoin biosynthesis I
glycogen degradation II
(<i>R,R</i>)−butanediol biosynthesis
fatty acid biosynthesis initiation II
fatty acids biosynthesis (yeast)
oleate biosynthesis II (animals)
γ−linolenate biosynthesis II (animals)
acyl carrier protein metabolism
pseudouridine degradation
zymosterol biosynthesis
ergosterol biosynthesis
glycerol−3−phosphate shuttle
5−aminoimidazole ribonucleotide biosynthesis I
5−aminoimidazole ribonucleotide biosynthesis II
inosine−5'−phosphate biosynthesis II
adenosine nucleotides <i>de novo</i> biosynthesis
dolichol and dolichyl phosphate biosynthesis
lanosterol biosynthesis
6−hydroxymethyl−dihydropterin diphosphate biosynthesis
<i>S</i>−adenosyl−L−methionine cycle I
3−dehydroquinate biosynthesis I
flavin biosynthesis III (fungi)
superpathway of 5−aminoimidazole ribonucleotide biosynthesis
selenocysteine biosynthesis II (archaea and eukaryotes)
cysteine biosynthesis IV (fungi)
galactose degradation I (Leloir pathway)
acetaldehyde biosynthesis II
acetaldehyde biosynthesis I
phosphate acquisition
D−<i>myo</i>−inositol (1,4,5)−trisphosphate biosynthesis
3−phosphoinositide biosynthesis
phosphate utilization in cell wall regeneration
superpathway of D−<i>myo</i>−inositol (1,4,5)−trisphosphate metabolism
1D−<i>myo</i>−inositol hexakisphosphate biosynthesis I (from Ins(1,4,5)P3)
D−<i>myo</i>−inositol (1,4,5)−trisphosphate degradation
D−<i>myo</i>−inositol (1,3,4)−trisphosphate biosynthesis
<i>meso</i>−butanediol biosynthesis I
<i>meso</i>−butanediol biosynthesis II
sitosterol biosynthesis
4−aminobutyrate degradation III
p−aminobenzoate biosynthesis
pyrimidine ribonucleosides degradation II
adenine and adenosine salvage II
guanosine nucleotides degradation II
superpathway of tetrahydrofolate biosynthesis
tetrahydrofolate salvage from 5,10−methenyltetrahydrofolate
tetrahydrofolate biosynthesis
adenine and adenosine salvage VI
superpathway of phenylalanine biosynthesis
superpathway of tryptophan biosynthesis
galactose degradation IV
NAD salvage pathway I
acetyl−CoA biosynthesis (from pyruvate)
xanthine and xanthosine salvage
S−adenosyl−L−methionine biosynthesis
superpathway of serine and glycine biosynthesis I
serine biosynthesis
sulfate reduction I (assimilatory)
sphingolipid metabolism
thioredoxin pathway
threonine biosynthesis
trehalose biosynthesis I
triacylglycerol biosynthesis
tRNA charging pathway
tryptophan biosynthesis
UDP−<i>N</i>−acetyl−D−glucosamine biosynthesis II
valine degradation I
valine biosynthesis
xylose degradation I
