# Supplementary material for: Differential RNA-seq, Multi-Network Analysis and Metabolic Regulation Analysis of Kluyveromyces marxianus Reveals a Compartmentalised Response to Xylose
Source: PLoS One. 2016 Jun 17;11(6):e0156242. doi: 10.1371/journal.pone.0156242 (PMC4912071; doi:10.1371/journal.pone.0156242)
Supplement: S3 Pathway — (PPTX) [file pone.0156242.s010.pptx]

## Slide 1
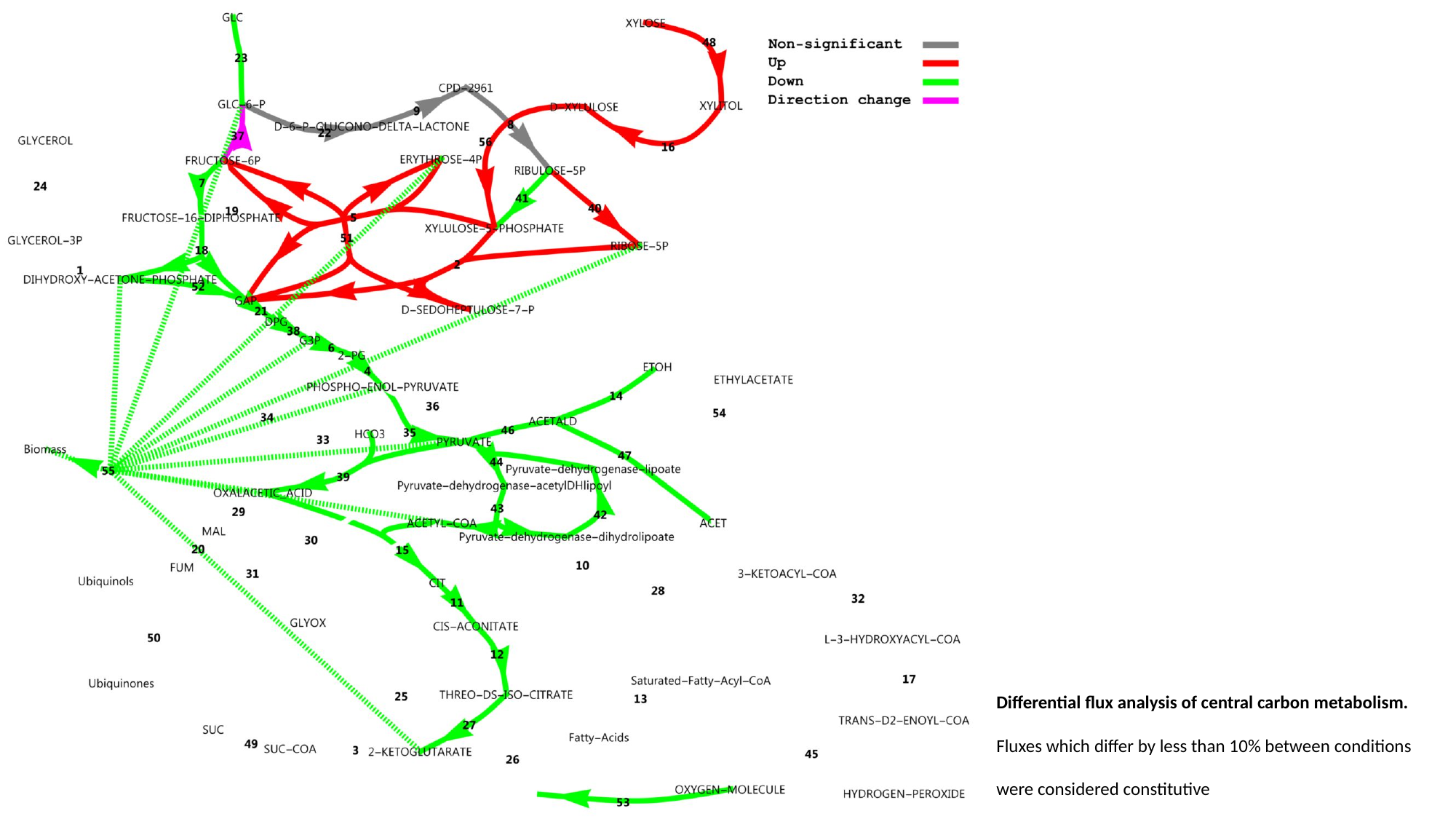

Differential flux analysis of central carbon metabolism.
Fluxes which differ by less than 10% between conditions were considered constitutive

## Slide 2
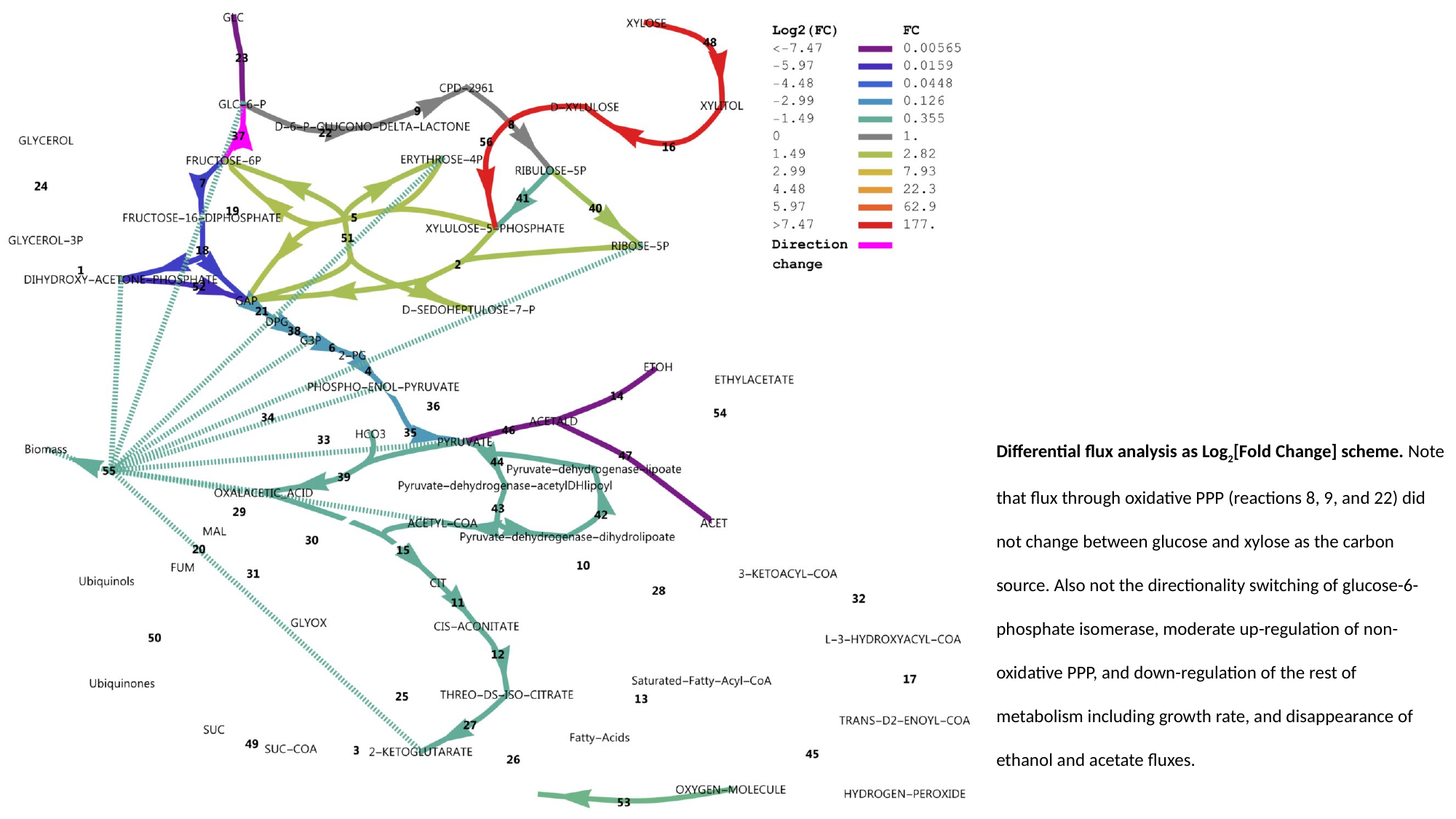

Differential flux analysis as Log2[Fold Change] scheme. Note that flux through oxidative PPP (reactions 8, 9, and 22) did not change between glucose and xylose as the carbon source. Also not the directionality switching of glucose-6-phosphate isomerase, moderate up-regulation of non-oxidative PPP, and down-regulation of the rest of metabolism including growth rate, and disappearance of ethanol and acetate fluxes.

## Slide 3
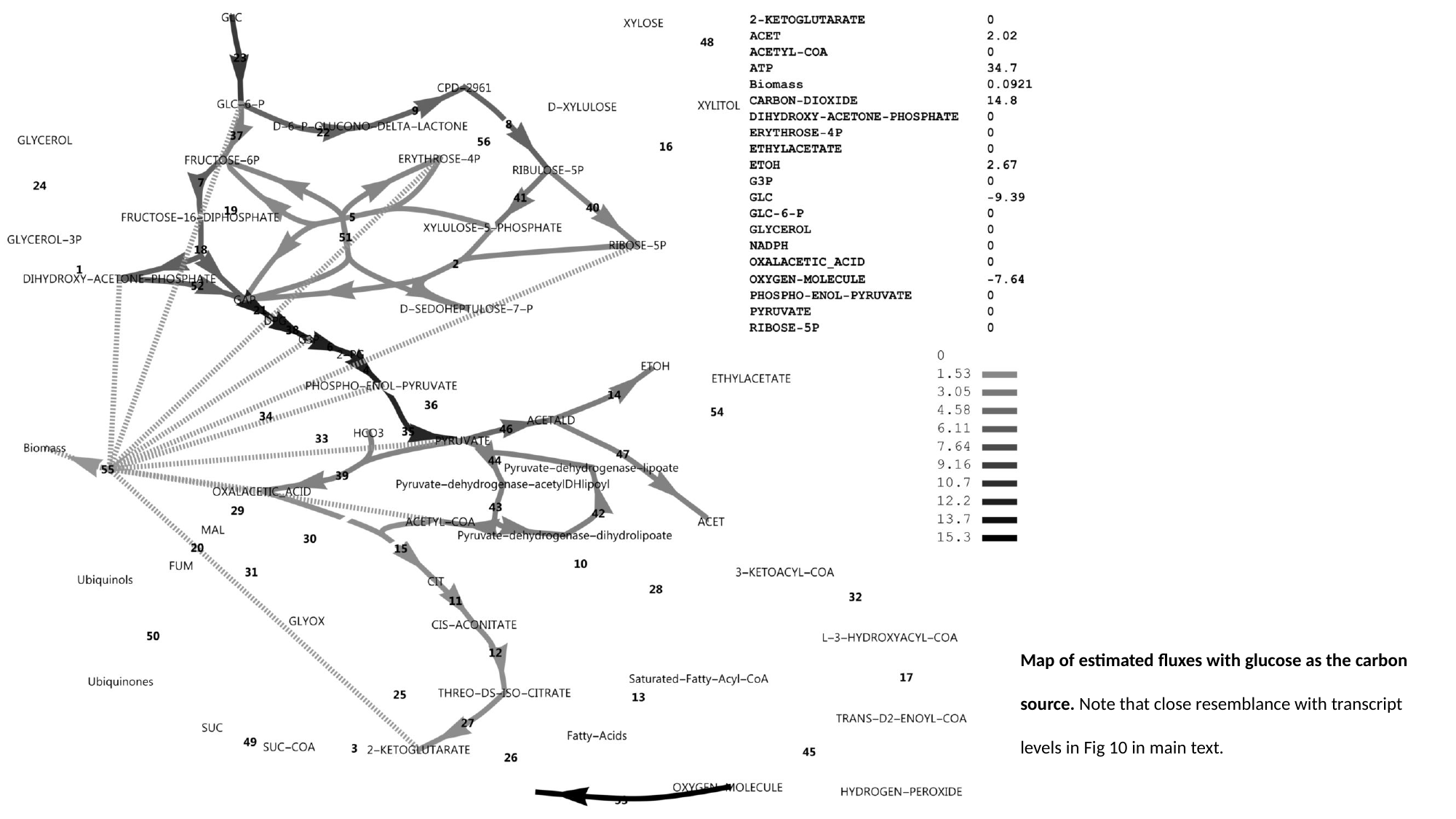

Map of estimated fluxes with glucose as the carbon source. Note that close resemblance with transcript levels in Fig 10 in main text.

## Slide 4
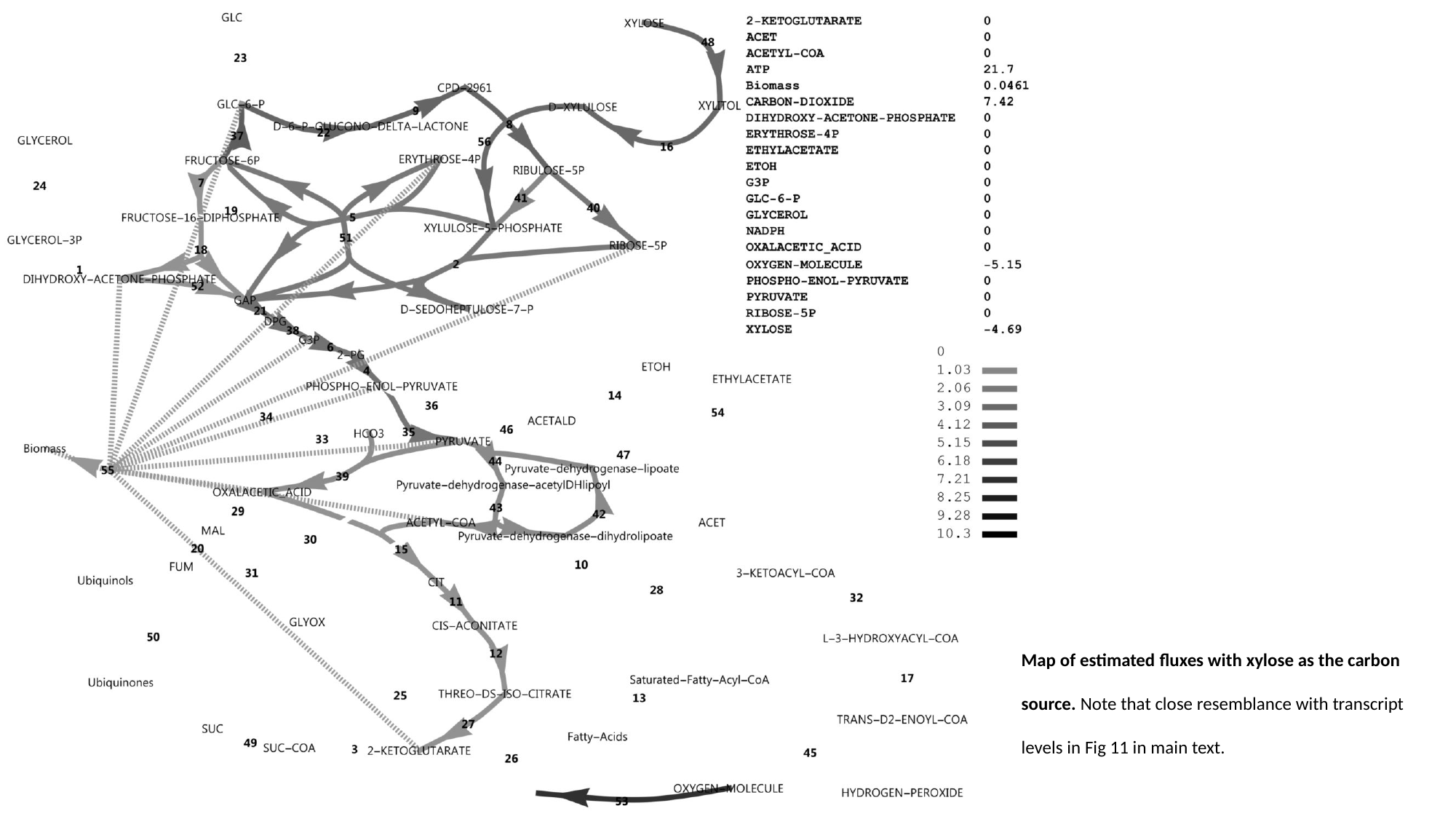

Map of estimated fluxes with xylose as the carbon source. Note that close resemblance with transcript levels in Fig 11 in main text.

## Slide 5
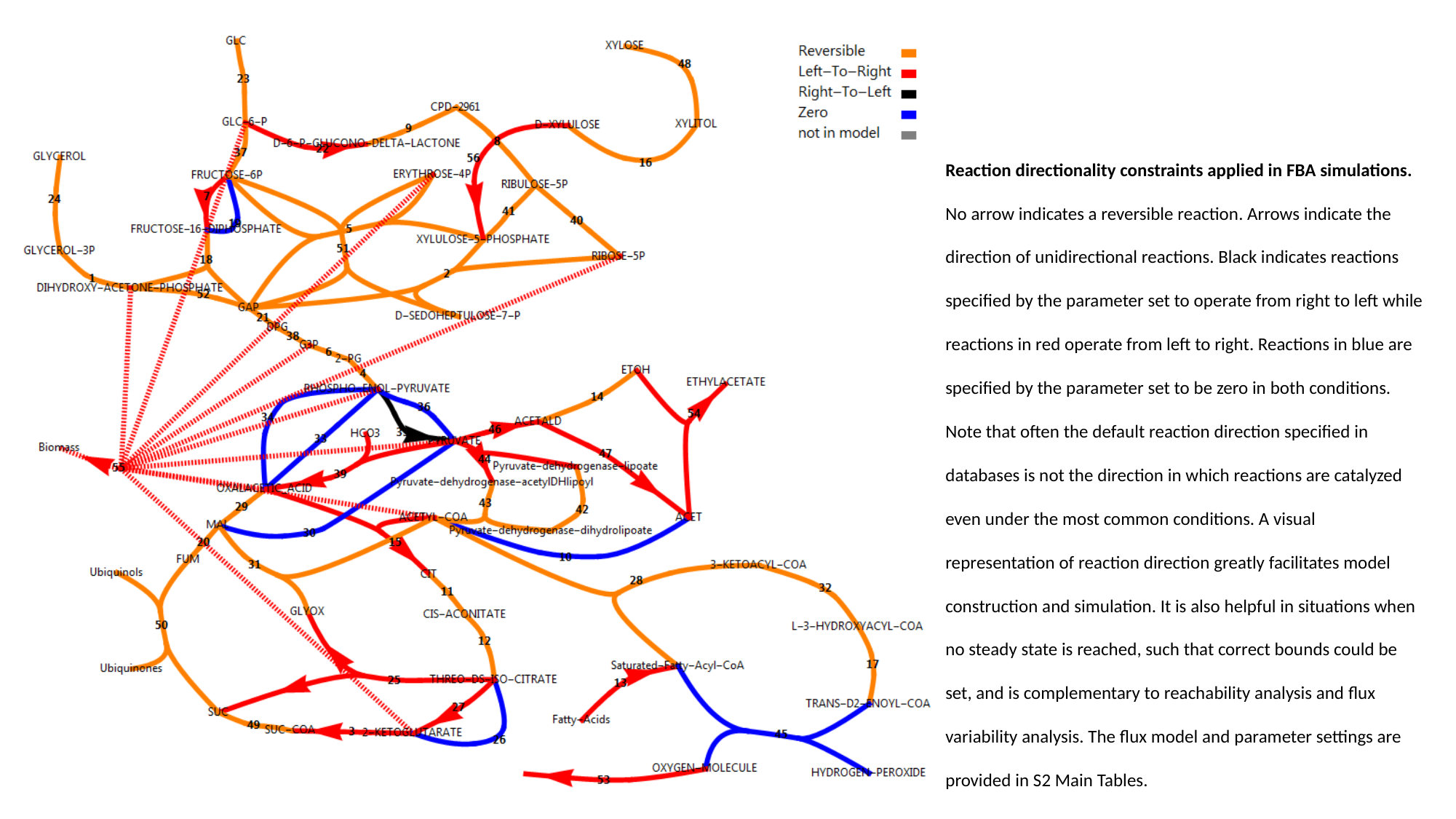

Reaction directionality constraints applied in FBA simulations. No arrow indicates a reversible reaction. Arrows indicate the direction of unidirectional reactions. Black indicates reactions specified by the parameter set to operate from right to left while reactions in red operate from left to right. Reactions in blue are specified by the parameter set to be zero in both conditions. Note that often the default reaction direction specified in databases is not the direction in which reactions are catalyzed even under the most common conditions. A visual representation of reaction direction greatly facilitates model construction and simulation. It is also helpful in situations when no steady state is reached, such that correct bounds could be set, and is complementary to reachability analysis and flux variability analysis. The flux model and parameter settings are provided in S2 Main Tables.

## Slide 6
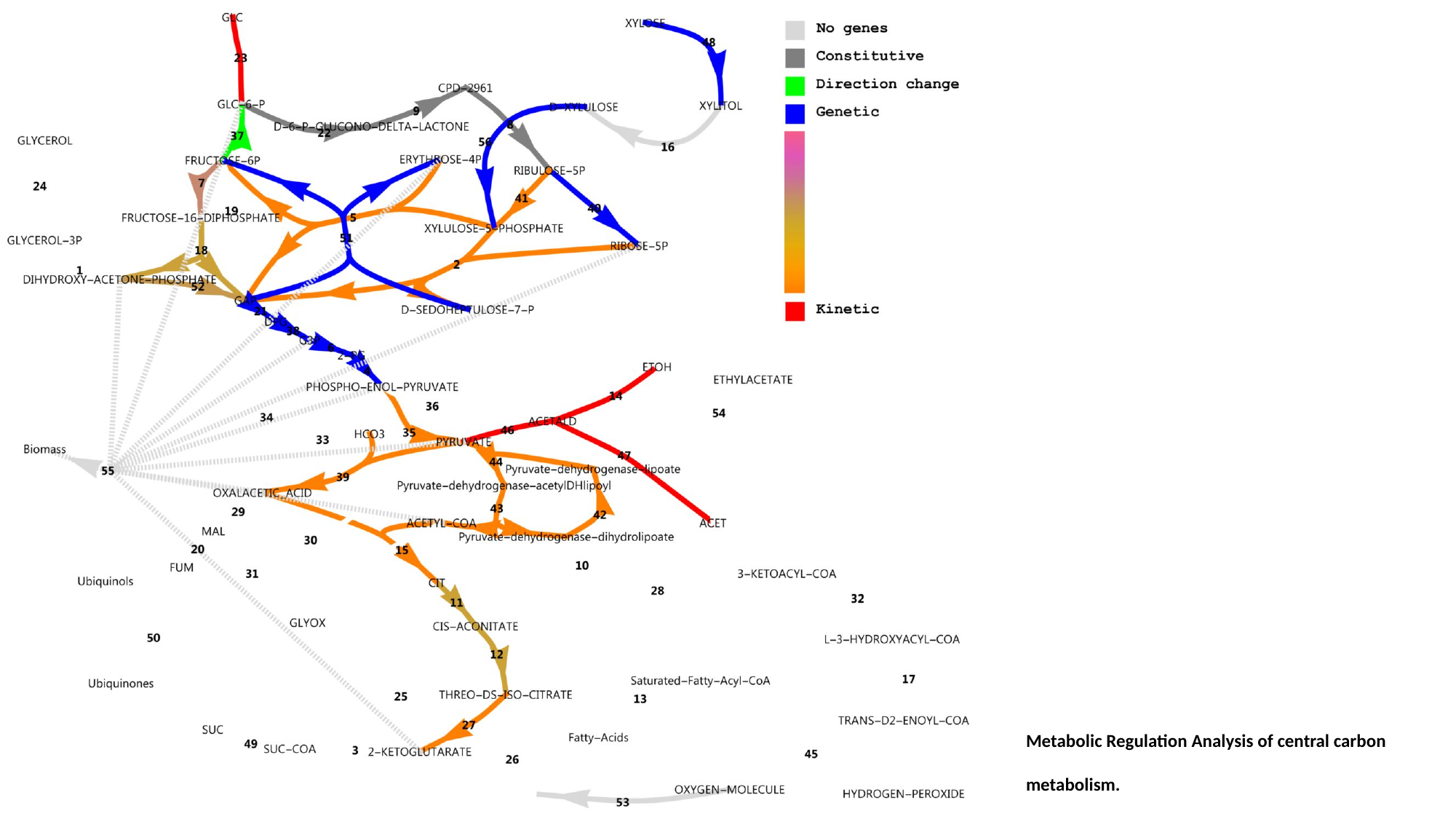

Metabolic Regulation Analysis of central carbon metabolism.

## Slide 7
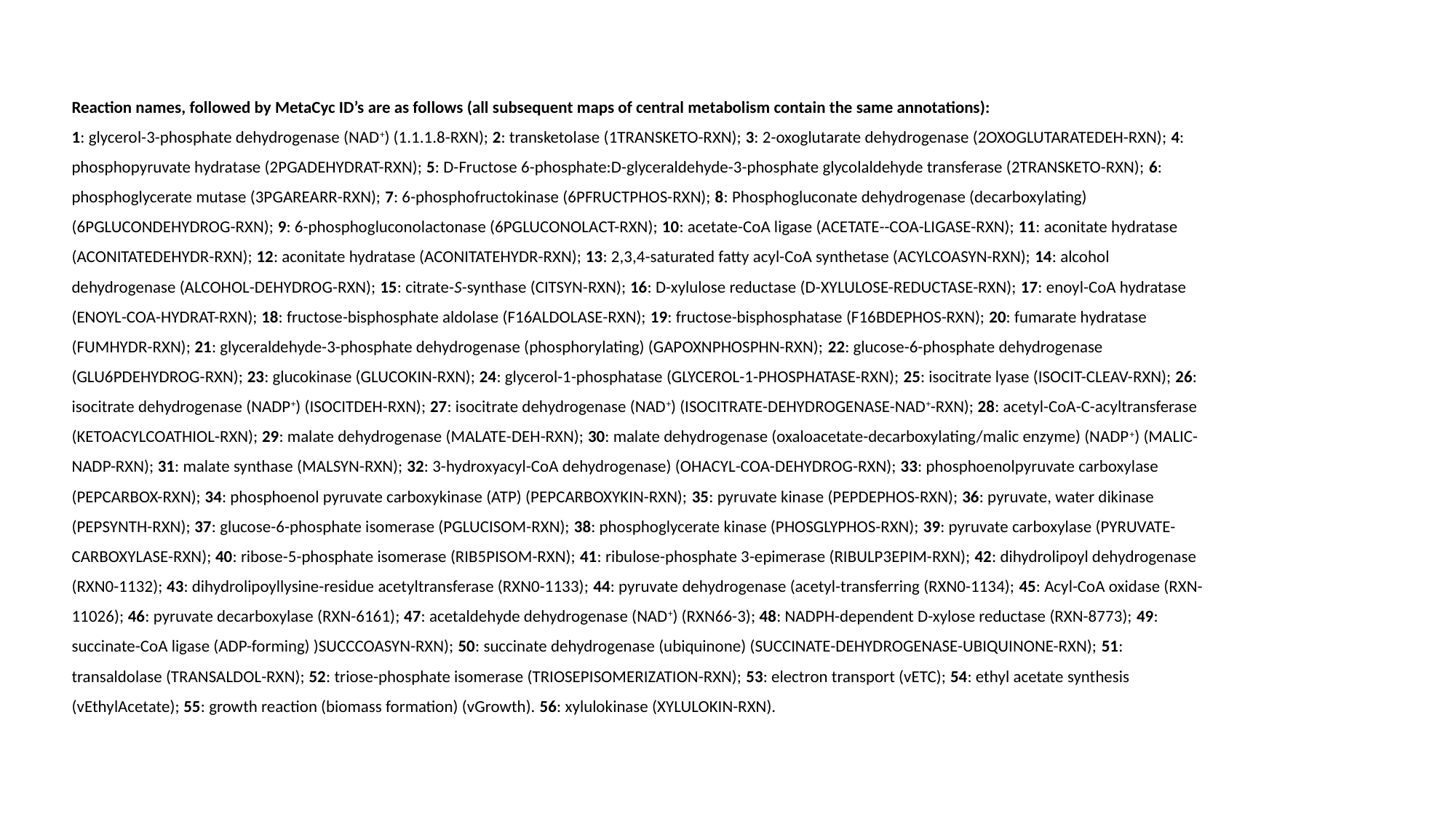

Reaction names, followed by MetaCyc ID’s are as follows (all subsequent maps of central metabolism contain the same annotations):
1: glycerol-3-phosphate dehydrogenase (NAD+) (1.1.1.8-RXN); 2: transketolase (1TRANSKETO-RXN); 3: 2-oxoglutarate dehydrogenase (2OXOGLUTARATEDEH-RXN); 4: phosphopyruvate hydratase (2PGADEHYDRAT-RXN); 5: D-Fructose 6-phosphate:D-glyceraldehyde-3-phosphate glycolaldehyde transferase (2TRANSKETO-RXN); 6: phosphoglycerate mutase (3PGAREARR-RXN); 7: 6-phosphofructokinase (6PFRUCTPHOS-RXN); 8: Phosphogluconate dehydrogenase (decarboxylating) (6PGLUCONDEHYDROG-RXN); 9: 6-phosphogluconolactonase (6PGLUCONOLACT-RXN); 10: acetate-CoA ligase (ACETATE--COA-LIGASE-RXN); 11: aconitate hydratase (ACONITATEDEHYDR-RXN); 12: aconitate hydratase (ACONITATEHYDR-RXN); 13: 2,3,4-saturated fatty acyl-CoA synthetase (ACYLCOASYN-RXN); 14: alcohol dehydrogenase (ALCOHOL-DEHYDROG-RXN); 15: citrate-S-synthase (CITSYN-RXN); 16: D-xylulose reductase (D-XYLULOSE-REDUCTASE-RXN); 17: enoyl-CoA hydratase (ENOYL-COA-HYDRAT-RXN); 18: fructose-bisphosphate aldolase (F16ALDOLASE-RXN); 19: fructose-bisphosphatase (F16BDEPHOS-RXN); 20: fumarate hydratase (FUMHYDR-RXN); 21: glyceraldehyde-3-phosphate dehydrogenase (phosphorylating) (GAPOXNPHOSPHN-RXN); 22: glucose-6-phosphate dehydrogenase (GLU6PDEHYDROG-RXN); 23: glucokinase (GLUCOKIN-RXN); 24: glycerol-1-phosphatase (GLYCEROL-1-PHOSPHATASE-RXN); 25: isocitrate lyase (ISOCIT-CLEAV-RXN); 26: isocitrate dehydrogenase (NADP+) (ISOCITDEH-RXN); 27: isocitrate dehydrogenase (NAD+) (ISOCITRATE-DEHYDROGENASE-NAD+-RXN); 28: acetyl-CoA-C-acyltransferase (KETOACYLCOATHIOL-RXN); 29: malate dehydrogenase (MALATE-DEH-RXN); 30: malate dehydrogenase (oxaloacetate-decarboxylating/malic enzyme) (NADP+) (MALIC-NADP-RXN); 31: malate synthase (MALSYN-RXN); 32: 3-hydroxyacyl-CoA dehydrogenase) (OHACYL-COA-DEHYDROG-RXN); 33: phosphoenolpyruvate carboxylase (PEPCARBOX-RXN); 34: phosphoenol pyruvate carboxykinase (ATP) (PEPCARBOXYKIN-RXN); 35: pyruvate kinase (PEPDEPHOS-RXN); 36: pyruvate, water dikinase (PEPSYNTH-RXN); 37: glucose-6-phosphate isomerase (PGLUCISOM-RXN); 38: phosphoglycerate kinase (PHOSGLYPHOS-RXN); 39: pyruvate carboxylase (PYRUVATE-CARBOXYLASE-RXN); 40: ribose-5-phosphate isomerase (RIB5PISOM-RXN); 41: ribulose-phosphate 3-epimerase (RIBULP3EPIM-RXN); 42: dihydrolipoyl dehydrogenase (RXN0-1132); 43: dihydrolipoyllysine-residue acetyltransferase (RXN0-1133); 44: pyruvate dehydrogenase (acetyl-transferring (RXN0-1134); 45: Acyl-CoA oxidase (RXN-11026); 46: pyruvate decarboxylase (RXN-6161); 47: acetaldehyde dehydrogenase (NAD+) (RXN66-3); 48: NADPH-dependent D-xylose reductase (RXN-8773); 49: succinate-CoA ligase (ADP-forming) )SUCCCOASYN-RXN); 50: succinate dehydrogenase (ubiquinone) (SUCCINATE-DEHYDROGENASE-UBIQUINONE-RXN); 51: transaldolase (TRANSALDOL-RXN); 52: triose-phosphate isomerase (TRIOSEPISOMERIZATION-RXN); 53: electron transport (vETC); 54: ethyl acetate synthesis (vEthylAcetate); 55: growth reaction (biomass formation) (vGrowth). 56: xylulokinase (XYLULOKIN-RXN).
