# Supplementary material for: Differential RNA-seq, Multi-Network Analysis and Metabolic Regulation Analysis of Kluyveromyces marxianus Reveals a Compartmentalised Response to Xylose
Source: PLoS One. 2016 Jun 17;11(6):e0156242. doi: 10.1371/journal.pone.0156242 (PMC4912071; doi:10.1371/journal.pone.0156242)
Supplement: S1 Network — (PDF) [file pone.0156242.s002.pdf]

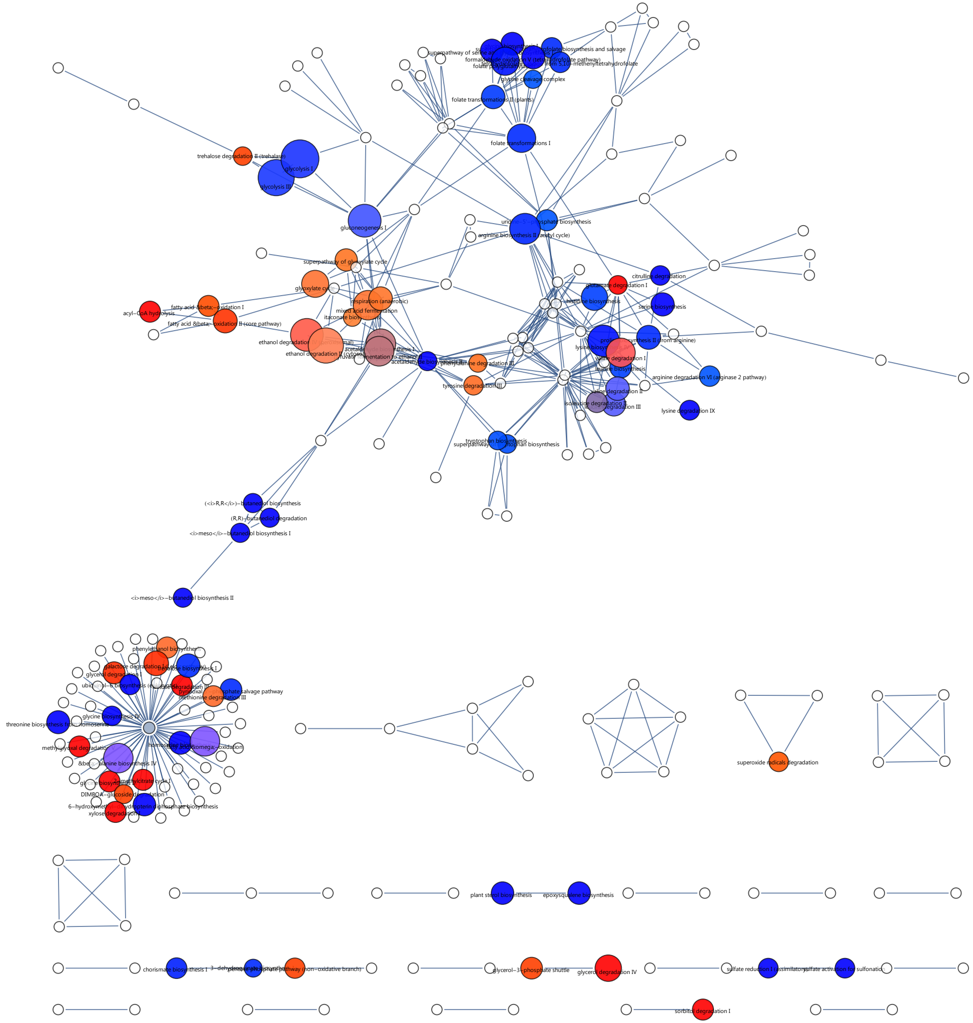
trehalose degradation II (trehalase)
glycolysis I
glycolysis III
gluconeogenesis I
formaldehyde oxidation V (tetrahydrofolate pathway)
glycine cleavage complex
folate transformations II (plants)
folate transformations I
arginine biosynthesis II (acetyl cycle)
superpathway of glyoxylate cycle
glyoxylate cycle
fatty acid β−oxidation I
acyl−CoA hydrolysis
fatty acid β−oxidation II (core pathway)
respiration (anaerobic)
mixed acid fermentation
ethanol degradation II (cytosol)
phenylalanine degradation III
tyrosine degradation III
tryptophan biosynthesis
citrulline degradation
glutamate degradation I
histidine biosynthesis
serine biosynthesis
valine degradation I
leucine biosynthesis
arginine degradation VI (arginase 2 pathway)
valine degradation II
lysine degradation IX
(<i>R,R</i>)−butanediol biosynthesis
(R,R)−butanediol degradation
<i>meso</i>−butanediol biosynthesis I
<i>meso</i>−butanediol biosynthesis II
methionine degradation III
glycine biosynthesis IV
methylglyoxal degradation
β−alanine biosynthesis IV
DIMBOA−glucoside degradation
superoxide radicals degradation
plant sterol biosynthesis
epoxysqualene biosynthesis
chorismate biosynthesis I
pentose phosphate pathway (non−oxidative branch)
glycerol−3−phosphate shuttle
glycerol degradation IV
sulfate reduction I (assimilatory)
sorbitol degradation I
