# Supplementary material for: Differential RNA-seq, Multi-Network Analysis and Metabolic Regulation Analysis of Kluyveromyces marxianus Reveals a Compartmentalised Response to Xylose
Source: PLoS One. 2016 Jun 17;11(6):e0156242. doi: 10.1371/journal.pone.0156242 (PMC4912071; doi:10.1371/journal.pone.0156242)
Supplement: S1 Text — (PDF) [file pone.0156242.s017.pdf]

Supplementary Text to 'Differential RNA-seq, Multi-Network Analysis and Metabolic Regulation Analysis of *Kluyveromyces marxianus* Reveals a Compartmentalized Response to Xylose'

Du Toit WP Schabort*, Precious K Letebele, Laurinda Steyn, Stephanus G Kilian and James C du Preez

Department of Microbial, Biochemical and Food Biotechnology, University of the Free State, Bloemfontein, South Africa

*For correspondence: schabortdwp@ufs.ac.za

Gene set enrichment using the full Gene Ontology

The enrichment scores of GO term gene sets are provided in supplementary file S2 Main Tables.

Sugar utilization

Some interesting aspects arose from the data. Firstly, a variety of carbohydrate utilization genes were differentially expressed on glucose vs xylose, mostly for utilization of the hexose galactose, and the pentoses xylose and arabinose. Even though these alternative sugars were not present in the medium, their utilization pathways were up-regulated, a phenomenon typical of alleviation of glucose repression. Secondly, the capacity for hexose utilization by hexokinase was constitutive and high. Thirdly, the initial steps in galactose, xylose and arabinose utilization were more strongly up-regulated as compared to later steps in each pathway.

Extracellular enzymes

Since *K. marxianus* seems to adopt an opportunistic metabolic gene set profile in response to the presence of xylose (or absence of glucose), the question arises whether extracellular hydrolases

possessing β-glucosidase activity, such as cellulases, would be activated, allowing *K. marxianus* to thrive on decaying plant matter. Cellulases are prevalent in fungi but not as prevalent in yeasts. The strain possesses fourteen genes annotated as having some form of β-glucosidase activity, most of which are secreted (supplementary Table 1 below). Beta-glucosidase (EC 3.2.1.21, cellobiase, gentiobiase) is a likely candidate that could function to hydrolyze cellobiose, the disaccharide found after hydrolysis by cellulose. It is 48.8-fold up-regulated and transcribed at a high level on xylose. It would be interesting to see whether this strain can grow on cellobiose, which is a trait uncommon to yeasts. Cellulose degradation is a complex process and requires the initial degradation of cellulose to cellobiose by cellulases. No cellulases were found in the genome, however. No genes were found to have xylanase activity or related terms either. Additional searches were performed for proteases, peptidases and lipases, which would be typical of opportunistic yeasts, especially pathogens. A number of proteases, peptidases and lipases were found, but none of them are clearly secreted proteins.

Supplementary Table 1. Enzymes with glucosidase and related activities.

ID	val(Glc)	val(Xyl)	log2(FC)	q	signt	Entry	Protein names	Gene names	EC number
g3519.t1	33.1	1601.0	5.6	0.001	yes	P07337	Beta-glucosidase (Cellobiase) (Gentiobiase)		3.2.1.21
g3265.t1	135.8	347.4	1.4	0.016	yes	Q04951	Probable family 17 glucosidase SCW10	SCW10 YMR305C YM9952.07C	3.2.1.-
g3867.t1	262.7	582.8	1.1	0.047	yes	Q12628	Glucan 1,3-beta-glucosidase (EC 3.2.1.58)	KLLA0C05324g	3.2.1.58
g4768.t1	841.6	1765.9	1.1	0.062	no	P53334	Probable family 17 glucosidase SCW4	SCW4 YGR279C	3.2.1.-
g3788.t1	922.9	1826.6	1.0	0.094	no	P15703	Glucan 1,3-beta-glucosidase	BGL2 SCW9 YGR282C	3.2.1.58
g4569.t1	146.2	285.3	1.0	0.090	no	Q06625	Glycogen debranching enzyme	GDB1 YPR184W	2.4.1.25; 3.2.1.33
g3018.t1	754.8	1235.7	0.7	0.262	no	P53616	Probable secreted beta-glucosidase SUN4	SUN4 SCW3 YNL066W N2411 YNL2411W	3.2.1.-
g2805.t1	25.3	39.4	0.6	0.430	no	P08019	Glucoamylase, intracellular sporulation-specific	SGA1 SGA YIL099W	3.2.1.3

g1329.t1	48.5	69.2	0.5	0.491	no	Q12168	Endo-1,3(4)-beta-glucanase 2 (Laminarinase-2)	ACF2 ENG2 PCA1 YLR144C L3180	3.2.1.6
g4202.t1	192.4	242.9	0.3	0.662	no	P32486	Beta-glucan synthesis-associated protein KRE6	KRE6 CWH48 YPR159W	
g2867.t1	136.4	148.0	0.1	0.904	no	P53189	Probable family 17 glucosidase SCW11	SCW11 YGL028C	3.2.1.-
g1495.t1	77.8	67.8	-0.2	0.829	no	P38138	Glucosidase 2 subunit alpha	ROT2 GLS2 YBR229C YBR1526	3.2.1.84
g2866.t1	51.4	42.4	-0.3	0.759	no	P53008	Mannosyl-oligosaccharide glucosidase (Glucosidase I)	CWH41 GLS1 YGL027C	3.2.1.106
g3564.t1	241.1	164.6	-0.6	0.441	no	P53753	Endo-1,3(4)-beta-glucanase 1 (Laminarinase-1)	DSE4 ENG1 YNR067C N3547	3.2.1.6

Sexual reproduction and invasive growth

Yeasts that reproduce sexually through mating have a pheromone sensing system whereby the haploid α-cells secrete a pheromone, mating factor α-1, which is sensed by the a-cells, a signal that suppresses DNA synthesis in the a-cells, thus synchronizing them with α-cells for conjugation**.** Mating factor α-1 (*MF(ALPHA)1*, *YPL187W*) was up-regulated 53-fold on xylose, whereas barrierpepsin (*BAR1*, *YIL015W*) was up-regulated 17-fold. Barrierpepsin likely cleaves mating factor α and fine-tunes the concentration of the pheromone for optimal conjugation [1]. Similarly, protein *SST2* (*YLR452C*), which also responds to α-pheromone to desensitize cells to α-pheromone, was 3.3-fold down-regulated [2]. A functional interaction between cells during conjugation is made by agglutinins in response to pheromones, of which α-agglutinin was up-regulated 5.7-fold. The flocculation proteins *FLO1* (*YAR050W*), *FLO11* (*YIR019C*), and *FLO9* (*YAL063C*) were also up-regulated. They form functional interactions by causing cell aggregation in liquid culture and are known to be involved in the formation of diploid pseudohyphae, which play a role in the adhesion of cells to substrates, invasive growth by haploid cells (see [3] for a review). It was shown that FLO11 is dominant in invasive growth whereas FLO1 is dominant in flocculation, while they are both under the control of the transcription factor encoded by the MSS1 gene [3]. In summary, both sexual reproduction was up-regulated as well as a variety of genes involved with morphological changes that may be beneficial for penetration into a solid substrate. We observed only

few pseudohyphae on xylose towards the stationary phase and none on glucose, indicating that the response is not manifested completely as a phenotype under these conditions, but likely further stimuli are needed to make a transition to invasive growth.

References

1. MacKay V. Welch SK, Insley MY, Manney TR, Holly J, Saari GC, et al. The *Saccharomyces cerevisiae* BAR1 gene encodes an exported protein with homology to pepsin. Proc. Natl. Acad. Sci. USA. 1988;85: 55-59.

2. Apanovitch DM, Slep KC, Sigler PB, Dohlman HG. Sst2 Is a GTPase-activating protein for Gpa1: Purification and characterization of a cognate RGS−Gα protein pair in yeast. Biochemistry. 1998;37(14): 4815-4822.

3. Bester MC, Pretorius IS, Bauer FF. The regulation of *Saccharomyces cerevisiae* FLO gene expression and Ca^{2+}-dependent flocculation by Flo8p and Mss11p. Curr. Genet. 2006;49: 375-383.
